# Educational Outreach with an Integrated Clinical Tool for Nurse-Led Non-communicable Chronic Disease Management in Primary Care in South Africa: A Pragmatic Cluster Randomised Controlled Trial

**DOI:** 10.1371/journal.pmed.1002178

**Published:** 2016-11-22

**Authors:** Lara R. Fairall, Naomi Folb, Venessa Timmerman, Carl Lombard, Krisela Steyn, Max O. Bachmann, Eric D. Bateman, Crick Lund, Ruth Cornick, Gill Faris, Thomas Gaziano, Daniella Georgeu-Pepper, Merrick Zwarenstein, Naomi S. Levitt

**Affiliations:** 1 Knowledge Translation Unit, University of Cape Town Lung Institute, Cape Town, South Africa; 2 Department of Medicine, University of Cape Town, Cape Town, South Africa; 3 Chronic Disease Initiative for Africa, Department of Medicine, University of Cape Town, Cape Town, South Africa; 4 Biostatistics Unit, Medical Research Council, Cape Town, South Africa; 5 Norwich Medical School, University of East Anglia, Norwich, United Kingdom; 6 Alan J Flisher Centre for Public Mental Health, Department of Psychiatry and Mental Health, University of Cape Town, Cape Town, South Africa; 7 Division of Cardiovascular Medicine, Brigham & Women’s Hospital, Boston, Massachusetts, United States; 8 Centre for Studies in Family Medicine, Department of Family Medicine, Schulich School of Medicine and Dentistry, Western University, London, Ontario, Canada; Harvard University, UNITED STATES

## Abstract

**Background:**

In many low-income countries, care for patients with non-communicable diseases (NCDs) and mental health conditions is provided by nurses. The benefits of nurse substitution and supplementation in NCD care in high-income settings are well recognised, but evidence from low- and middle-income countries is limited. Primary Care 101 (PC101) is a programme designed to support and expand nurses’ role in NCD care, comprising educational outreach to nurses and a clinical management tool with enhanced prescribing provisions. We evaluated the effect of the programme on primary care nurses’ capacity to manage NCDs.

**Methods and Findings:**

In a cluster randomised controlled trial design, 38 public sector primary care clinics in the Western Cape Province, South Africa, were randomised. Nurses in the intervention clinics were trained to use the PC101 management tool during educational outreach sessions delivered by health department trainers and were authorised to prescribe an expanded range of drugs for several NCDs. Control clinics continued use of the Practical Approach to Lung Health and HIV/AIDS in South Africa (PALSA PLUS) management tool and usual training. Patients attending these clinics with one or more of hypertension (3,227), diabetes (1,842), chronic respiratory disease (1,157) or who screened positive for depression (2,466), totalling 4,393 patients, were enrolled between 28 March 2011 and 10 November 2011. Primary outcomes were treatment intensification in the hypertension, diabetes, and chronic respiratory disease cohorts, defined as the proportion of patients in whom treatment was escalated during follow-up over 14 mo, and case detection in the depression cohort. Primary outcome data were analysed for 2,110 (97%) intervention and 2,170 (97%) control group patients. Treatment intensification rates in intervention clinics were not superior to those in the control clinics (hypertension: 44% in the intervention group versus 40% in the control group, risk ratio [RR] 1.08 [95% CI 0.94 to 1.24; *p* = 0.252]; diabetes: 57% versus 50%, RR 1.10 [0.97 to 1.24; *p* = 0.126]; chronic respiratory disease: 14% versus 12%, RR 1.08 [0.75 to 1.55; *p* = 0.674]), nor was case detection of depression (18% versus 24%, RR 0.76 [0.53 to 1.10; *p* = 0.142]). No adverse effects of the nurses’ expanded scope of practice were observed. Limitations of the study include dependence on self-reported diagnoses for inclusion in the patient cohorts, limited data on uptake of PC101 by users, reliance on process outcomes, and insufficient resources to measure important health outcomes, such as HbA1c, at follow-up.

**Conclusions:**

Educational outreach to primary care nurses to train them in the use of a management tool involving an expanded role in managing NCDs was feasible and safe but was not associated with treatment intensification or improved case detection for index diseases. This notwithstanding, the intervention, with adjustments to improve its effectiveness, has been adopted for implementation in primary care clinics throughout South Africa.

**Trial Registration:**

The trial is registered with Current Controlled Trials (ISRCTN20283604)

## Abstract 2

In a cluster-randomized trial done in South Africa, Lara Fairall and co-workers investigate the effectiveness of a clinical management tool for non-communicable diseases in primary care.

## Author Summary

**Why Was This Study Done?:**

- Non-communicable diseases (NCDs) are the leading cause of deaths worldwide, even in low- and middle-income countries (LMICs) that continue to battle to control communicable diseases like HIV and tuberculosis (TB).
- Effective and affordable treatments prevent complications from NCDs like heart attacks and strokes, but access is limited by the variable availability and limited capacity of primary care health workers to detect and effectively manage these conditions. In many LMICs, non-physicians such as nurses provide primary care for NCDs.
- Over the past 16 years, we have developed, evaluated, and refined integrated clinical management tools and training programmes that employ problem-based approaches to common symptoms like cough and priority health conditions including TB, HIV, asthma, and emphysema. We have shown them to be effective in improving the quality and outcomes of care for communicable diseases.
- We have expanded this programme to include almost all NCDs and mental health. This study evaluated the impact, both benefits and harms, of introducing the expanded programme, called Primary Care 101 (PC101), in terms of the quality of primary care for four common chronic diseases: hypertension, diabetes, chronic respiratory disease, and depression.

**What Did the Researchers Do and Find?:**

- We compared the care offered to patients with one of these four chronic diseases in 18 clinics in which primary care health workers were trained in the use of PC101 with that in 18 clinics where nurses continued to use the predecessor tool, which focused on communicable diseases.
- The trial had a pragmatic design, meaning it was conducted under usual conditions of health system operational constraints. Clinics in urban and rural areas serving people living in socio-economically deprived areas of South Africa were selected.
- We enrolled 4,393 patients with one or more of the NCDs of interest and followed them up for 14 mo after introduction of PC101 at the intervention clinics. The primary outcome of interest was intensification of treatment (or diagnosis, in the case of depression) for the four NCDs, analysed separately.
- The results confirmed very high rates of multimorbidity (patients having more than one condition at a time), under-diagnosis, under-treatment, and poor disease control.
- Introducing PC101 did not result in intensification of treatment for the four NCDs, but neither was there evidence of harm from the nurses’ expanded scope of practice.

**What Do These Findings Mean?:**

- The trial confirmed that multimorbidity and poor detection and control of NCDs and depression are common in this setting. Interventions are necessary to limit the impact of these conditions on people’s health and quality of life.
- PC101 offered a practical and acceptable tool to help expand the scope of practice of non-physician clinicians to include NCD care, but we were not able to show improvements in care, as we have previously done for communicable diseases.
- The study illustrates the limitations of trials designed to study the effects of complex system interventions in real life, where even small changes across many endpoints, as seen in our study, may be useful to decision-makers under pressure to respond constructively to the rise of multimorbidity and NCDs.
- PC101 has been adopted for country-wide implementation in primary care clinics in South Africa.

## Introduction

South Africa is facing a quadruple burden of disease: HIV and tuberculosis (TB); non-communicable diseases (NCDs), including mental health conditions; injury and violence; and maternal, neonatal, and childhood illnesses [1]. The past 15 years have seen concentrated efforts to strengthen the capacity of the public health system to treat HIV and TB. These investments seem at last to be paying off, with a rise in life expectancy, a decline in mortality [2], and fewer new HIV infections [3]. Yet the burden of NCDs and mental health remains unchecked; cardiovascular disease is now the second leading cause of death in South Africans after communicable diseases [4,5].

In South Africa, responsibility for the detection and treatment of NCDs lies at the primary care level, with nurses seeing nine out of ten patients, most of whom have more than one presenting condition [6]. However, the quality of NCD care is generally poor, characterised by under-diagnosis, under-treatment, and poor clinical control [1,7,8]. We have previously successfully piloted and trialled task-sharing interventions for communicable diseases, increasing the capacity of nurses to take on assessment and prescribing roles for HIV and TB previously restricted to doctors [9–15]. This programme has been scaled up throughout South Africa as part of the national government’s accelerated response to HIV and TB launched in 2010 [16]. A similar programme has been developed for use in other countries including Malawi, Botswana, Brazil, and Mexico [17]. We have since expanded this programme, now called Primary Care 101 (PC101), to include NCDs and mental health, hoping to leverage the health system reforms that accompanied the scale-up of antiretroviral therapy (ART) to improve the quality of primary care for other priority conditions.

These integrated programmes of care seek to overcome the limitations of vertical services that tend to neglect multimorbidity [18–23], and to expand the roles of nurses, increasing the number and distribution of health workers providing treatment for common NCD conditions.

While the benefits of nurse substitution and supplementation for a limited number of NCDs in high-income settings are well recognised [24], evidence from low- and middle-income countries (LMICs) is sparse and limited to a few pilot studies [25–28]. Fewer studies still have sought to improve care across several NCDs simultaneously. Meta-analyses of complex interventions in health systems confirm only small effect sizes (ranging from 0.4% to 6.3%) for carer behaviour (improved care), but given the size of the populations affected, these effect sizes are considered important, provided the interventions are introduced without harm. We report here the findings of the PC101 Trial, a pragmatic cluster randomised study evaluating the effectiveness of the PC101 intervention, which combines provision of an integrated management tool with educational outreach to nurses. The primary outcomes of interest were intensification of treatment for hypertension, diabetes, and chronic respiratory disease and case detection of depression in overlapping cohorts of patients with these conditions.

## Methods

Ethical approval for the trial was obtained from the University of Cape Town Human Research Ethics Committee (reference number 119/2010) and the Western Cape Department of Health.

### Study Design

This was a pragmatic, parallel-group cluster randomised controlled trial performed in the Eden and Overberg districts of the Western Cape Province. Clusters were public sector primary healthcare clinics randomised within six sub-district strata. Outcome measures in each of four cohorts were assessed in individual patients. Patient cohorts overlapped; patients with more than one condition of interest were included in each applicable cohort, and cohorts were powered and analysed separately. This study design, with multiple cohorts, each with its own primary outcome evaluated simultaneously, aimed to reflect the realities in primary care clinics that nurses are required to diagnose and manage a wide range of conditions, that NCDs are associated with multimorbidity, and that a focus on one condition may compromise the management of others [29]. The Western Cape Department of Health provided consent for the inclusion and randomisation of clinics, before randomisation was performed. Patients provided written consent for data collection after randomisation of clinics and prior to data collection.

### Participants

#### Clinics

The study was conducted in the predominantly rural districts of Eden and Overberg, where public sector clinics serve a population of around 800,000, mainly people with lower socio-economic status. Busy town clinics had fulltime doctors, but most clinics were nurse led, with doctors in attendance on a sessional basis (Table A in S1 Appendix).

Eligible clinics provided services, including for NCDs, at least five days a week and reported more than 10,000 attendances per year, so were likely able to contribute sufficient numbers of patients to the study. Of 124 clinics in the Eden district, 33 clinics in five sub-districts met these criteria. We supplemented this sample with five clinics from a sub-district in an adjacent district (Overberg), to increase the number of clinics available for randomisation and strengthen the study’s power.

The health districts in the study are representative of health services offered to more than 80% of the population of South Africa, comprising clinics both from medium-sized towns and rural areas [6].

#### Patients

The study population comprised patients with one or more of the following: hypertension, diabetes, chronic respiratory disease, or depression. Initial eligibility criteria were being 18 y or older, likely to reside in the area for the next year, and capable of actively engaging in an interviewer-administered questionnaire at the time of recruitment. Inclusion criteria for the four cohorts were as follows (Table 1): for the hypertension and diabetes cohorts, if patients reported being on medication for hypertension or diabetes, respectively; for the respiratory disease cohort, if they reported receiving medication for chronic airway disease (asthma or chronic obstructive pulmonary disease [COPD]) or reported a cough and/or difficult breathing for 2 wk or more prior to enrolment and were not on treatment for TB [30]; for the depression cohort, if they scored ten or more on the 10-item Center for Epidemiologic Studies Depression Scale (CESD-10) [31,32]. We selected this instrument because the 20-item version has been validated in a similar setting in South Africa [33], and the 10-item version in primary care populations elsewhere [34–36]. The shorter version was necessary to limit the length of the screening process for all four conditions. Patients were eligible even if at the time of enrolment they had no record of current treatment for their condition.

**Table 1:** Eligibility criteria, primary outcome definitions, and required sample size estimates for each cohort.

Cohort	Eligibility Criteria	Primary Outcome	Primary Outcome Definition	Required Sample Size Parameters^1^			
				Cluster Size	Intervention Proportion	Control Proportion	ICC
Hypertension	Self-reported antihypertensive medication^2^	Treatment intensification	(1) an increase in the number of antihypertensive medication classes and/or (2) an increase in dose of at least one antihypertensive and/or (3) a switch to an antihypertensive in another medication class and/or (4) a switch to an antihypertensive in the same medication class provided that the new dose is equivalent to a higher dose of the previous antihypertensive and/or (5) addition of aspirin and/or (6) the addition or increase in dose of a statin.	57	0.36	0.25	0.04 [37]^3^
Diabetes	Self-reported hypoglycaemic medication^4^	Treatment intensification	(1) the addition or increase in dose of metformin and/or (2) the addition or increase in dose of a sulphonylurea and/or (3) the addition or increase in dose of insulin and/or (4) the addition or increase in dose of an ACE inhibitor and/or (5) addition of aspirin and/or (6) the addition or increase in dose of a statin.	57	0.36	0.25 [38]^3^	0.04 [37]^3^
Chronic respiratory disease	Self-reported respiratory medication OR cough and/or difficult breathing >2 wk (and not on TB treatment)^5^	Treatment intensification	(1) the addition or increase in dose of an inhaled corticosteroid and/or (2) addition of a beta-agonist and/or (3) addition of ipratropium bromide and/or (4) addition of theophylline.	36	0.30	0.15	0.02 [15]^3^
Depression	CESD-10 ≥ 10	Case detection	(1) addition of antidepressant medication and/or (2) receipt of counselling by a mental health practitioner and/or (3) referral to mental health services.	60	0.10	0.04	0.04 [39]^3^

^1^All calculations are for two-sided tests and are powered at 90%. Sample sizes have been inflated by 20% to allow for loss to follow-up at 14 mo.

^2^Patients included in this cohort responded yes to the following question: “Are you taking medicine for high blood pressure (hypertension)?”

^3^These parameters were derived from earlier publications.

^4^Patients included in this cohort responded yes to the following question: “Are you taking medicine for diabetes (“sugar”)?”

^5^Patients included in this cohort responded yes to either of the following questions: “Are you taking medicine for asthma or chronic bronchitis or emphysema?” or “Do you have cough or difficult breathing which has lasted for more than two weeks?”

CESD-10, 10-item Center for Epidemiologic Studies Depression Scale; ICC, intraclass correlation coefficient.

In keeping with the study’s pragmatic design, enrolment was not restricted to patients with uncontrolled disease or to patients considered to be adherent to current treatment [40]. Although encouraging adherence was included in the management tool, it was not monitored.

#### Randomisation

Clinics within each of six health sub-district strata were randomised to avoid potential confounding resulting from geographically determined differences in management of clinical services. Two strata contained equal numbers of clinics, meaning that randomisation could be done in a 1:1 ratio. The four strata containing odd numbers of clinics were randomly allocated to have either one more or one fewer intervention clinic than control clinics, to achieve an equal number of clinics in each group (19 per group, 38 in total).

Randomisation was completed by the trial statistician using nQuery Advisor after recruitment of clinics, independently of the managers giving permission for the clinics to be included in the trial, and prior to patient recruitment and implementation of the intervention.

### Setting and Programme

#### Usual care for non-communicable and communicable diseases (control group)

South African primary care clinics provide free services for communicable disease and NCDs. Patients are seen by a clinician, usually a nurse, and stable patients with NCDs are seen at intervals of 3–6 mo, but are required to collect medications each month either from the clinic or from an off-site medication pick-up point. The clinical load borne by nurses is great; in 2008 the median number of patients per nurse seen each working day in the clinics studied was 25. Although all clinics were attended by doctors, in more than half this was on a part-time basis rather than daily (Table A in S1 Appendix). National regulations require that prescriptions be renewed and co-signed by a doctor at least every 6 mo, a process that is time-consuming, reducing opportunities for the doctor to review complex cases and mentor nurses. The selection of medications and level of prescribing provisions (nurse versus doctor) are governed by the South African national essential medicines list and standard treatment guidelines [41], which are revised by the National Department of Health every 5 y. Nurse prescribing provisions differ by province and, prior to the trial, were limited in the Western Cape to first-line medications such as thiazide diuretics for hypertension, metformin for diabetes, and low-dose inhaled corticosteroids for asthma. Prescription of antidepressants, which is governed by regulatory conditions for high schedule medications, is restricted to doctors.

Guidelines and policies for communicable diseases change more frequently than those for NCDs. Guidelines for both tend to be lengthy and text-heavy, at times containing confusing differences in recommendations for the same condition. To address this issue, we developed and implemented the Practical Approach to Lung Health and HIV/AIDS in South Africa (PALSA PLUS), a management tool that integrates guidelines and configures them concisely and simply in an algorithmic format that more closely aligns with presentations in primary care (symptoms and follow-up for chronic conditions) and ensures harmonisation of disease-specific guidelines. It also clarifies prescriber levels (nurse versus doctor) [13,17,42]. It was implemented in two provinces in 2006 (Western Cape and Free State), and in all nine provinces of South Africa between 2010 and 2011. Since 2007 it has included provision for nurse initiation and re-prescription of ART. This inclusion was based on the results of a large pragmatic randomised controlled trial performed in the Free State Province that showed that nurses were as effective as doctors in providing ART care [9,11,12], and on a second trial in the Western Cape and Gauteng Provinces that evaluated nurse re-prescription of ART [43]. This development was prompted by the urgent need to expand ART services in South Africa. Use of the latest (2011/2012) version of PALSA PLUS was the standard of care in control clinics during the PC101 Trial. Prior to the development of PC101, nurses were required to manage NCDs, but they received relatively little training and support, resources for NCD management were not user-friendly, and initiation or intensification of NCD medications was largely dependent on the availability of doctors. The introduction of PALSA PLUS was the first attempt to change this pattern; providing more user-friendly management tools that expanded nurses’ scope of practice and prescribing with increasing “diagonal” integration.

A second key component of the PALSA PLUS programme is training clinicians to use the management tool. This component employs an educational outreach model [44] in which facility trainers, typically nurse middle managers, are trained and equipped to deliver repeated short (1.5 h), onsite, interactive training sessions using carefully constructed case scenarios [42]. Clinicians are trained to use the tool as a care pathway in case management and to use it during each consultation. Follow-up “refresher” training accompanied distribution of the revised management tool each year. By 2011, around 70% of all nurses working in the trial districts had received initial training in PALSA PLUS, and training continued as usual in the control clinics.

An unanticipated change in usual care in the health districts under study was a shift in focus from communicable disease care to NCD care. Midway through the trial, the district health department launched a 3-mo campaign called Chronic Disease Season in all clinics to improve NCD recognition and care. Chronic Disease Season focused on hypertension and diabetes and involved both community and clinic health workers. The community-level interventions included several “health screening days” in which free blood pressure and finger-prick glucose measurements were offered at venues such as shopping centres and town halls. People with high values were referred to local clinics. In addition, around 10% of community health workers (33 in total) were equipped to provide basic education on lifestyle measures including diet and physical activity.

#### Intervention rationale

The PC101 intervention was a further development of the PALSA PLUS programme, aimed to include all common symptoms and conditions, including NCDs, among adults attending primary care services. This expanded scope was strongly motivated by input from primary care nurses and managers, who reported that coverage for NCDs in PALSA PLUS, particularly hypertension and diabetes, would greatly improve its usefulness. The implementation of PC101 aimed to use the same educational outreach approach as used for PALSA PLUS. This educational approach was shown in three pragmatic trials to be effective for the management of communicable diseases. Beneficial effects included reproducible and substantial improvements in TB case detection [9,13,15]; increases in appropriate prescribing, including inhaled corticosteroids for asthma [15], co-trimoxazole prophylaxis for HIV [13], and appropriate switching to second-line ART [9]; and appropriate referral of severe [15] and complex cases [9]. Changes in healthcare utilisation included fewer and shorter hospital admissions and a higher number of primary care visits [9,13,15]. The impact on health worker morale was also documented in parallel qualitative evaluations, with nurses reporting a sense of empowerment and emphasising the value of combining simplified diagnostic and treatment algorithms, onsite training, and expansions in prescribing provisions [12,42]. No harmful effects of the intervention were noted.

#### Intervention materials

The main intervention material was a 101-page evidence- and policy-informed algorithmic management tool. Based on PALSA PLUS, it was developed over a period of 5 y (2006–2011) with input from specialist clinicians, primary care doctors and nurses, allied health professionals, managers, and representatives of patient advocacy groups. The selection of content was based on the results of a cross-sectional survey in 18,000 consultations in primary care clinics across four provinces in South Africa of the most common reasons for attendance. The first half of PC101 covers 40 of the most common symptoms in adults attending primary care and prompts screening for the 20 chronic conditions included in the second half of the tool [6]. The selection of chronic conditions took a health services approach, including those that required regular planned follow-up in primary care. Included were communicable diseases (HIV, TB, sexually transmitted infections), NCDs (diabetes, hypertension, asthma, COPD, epilepsy), mental health conditions (depression, substance abuse, schizophrenia, dementia), and women’s health (contraception, antenatal care). Content was extracted from existing disease and policy guidelines and structured in a simple summative form: one page for “diagnosis” and one to two pages for follow-up “routine care” (organised under the headings of “assess”, “advise”, and “treat”) for each condition. Promotion of integrated care was a key objective. Extensive use was made of algorithms and checklists to optimise presentation of content, and provide actionable support that is readily applied during consultations. Content for diverse conditions was organised in a standard format; symptom pages prompted screening for multiple chronic conditions, and pages on the routine care of chronic conditions included screening for common comorbidities. In addition, care was taken to ensure that recommendations that were applicable to multiple pages of the tool, such as blood pressure thresholds for diagnosis, treatment, and lifestyle advice, were harmonised and consistently reflected. The management tool was provided as a ring-bound, high-quality, full-colour illustrated booklet to every clinician (nurse and doctor) responsible for primary care in the 19 intervention clinics. The tool is updated annually to reflect changes in evidence, policy, and feedback from clinicians and managers. For examples of updated content, see http://knowledgetranslation.co.za/programmes/pack-adult/.

The case scenarios used for training built on a set that had been extensively used during PALSA PLUS implementation. An illustration of a typical waiting room scene provided a cast of characters, each of whom was fleshed out in a case scenario (Table B in S1 Appendix). The cases were carefully constructed to build familiarity with use of the management tool, grow knowledge specifically related to NCDs and depression, and scaffold development of knowledge and skills [45], moving from straightforward clinical presentations toward greater complexity and multimorbidity. The cases formed the basis for the educational training sessions (Table B in S1 Appendix). A desk-blotter with a calendar illustrating key messages for priority conditions was provided to all staff in intervention clinics, to facilitate booking of follow-up appointments and to remind clinicians of essential elements of care.

#### Training

Six health department nurse trainers with experience in primary care and with responsibility for existing training initiatives within the study districts—including Integrated Management of Childhood Illness, PALSA PLUS, and ad hoc training in the TB programme—and with a support role for nurses were employed as facility trainers for the study. They were initially trained in PC101 during a 5-d live-in training course in May 2011. This course was led by an experienced adult education practitioner with a background in nursing (G. F.) and the family doctor who had led the expansion of the management tool (R. C.). The programme adopted a strong experiential focus, and gave as much attention to equipping the nurse trainers to be educators as it did to the expanded content of the management tool. It included multiple practice sessions during which the nurse trainers facilitated case-scenario-based training sessions with their peers, followed by critical feedback. It included exercises to help each trainer understand their own learning style [46] and to learn reflective practice. Facility trainers delivered eight short (1.5 h), on-site, interactive educational outreach sessions using the PC101 management tool and case scenarios to all clinical staff at intervention clinics over several weeks. In all, 155 face-to-face educational outreach sessions were held at the 19 intervention clinics, eight sessions in each clinic. Owing to clinical demands and absences due to night duties or annual sick or study leave, not all staff were able to attend every session. In total, 81 nurses (who each participated in a median of six sessions), five pharmacists, and four doctors were trained. The trainers received no payment from the research team. In addition to on-site training, nurse trainers provided support to staff through regular visits during which they would discuss difficult cases, review folders of patients whose care nurses had changed using PC101, or jointly see patients.

The nurse trainers themselves were supported through quarterly 1-d workshops, facilitated by G. F. These workshops included opportunities to report back on training at the clinic, troubleshoot difficulties in scheduling or completing educational outreach sessions, resolve queries related to the clinical content of the management tool, and practise facilitation skills. They also aimed to continue the community of practice that had been established during the initial live-in training.

#### Expanded prescribing provisions

Professional nurses who successfully completed the educational outreach were authorised by the district manager to prescribe an additional seven medications for NCDs previously restricted to doctors: enalapril and amlodipine for hypertension, glibenclamide and gliclazide for diabetes, simvastatin for increased cardiovascular risk, inhaled budesonide for asthma, and short courses of oral prednisone for exacerbations of COPD (Table C in S1 Appendix). These expansions were clearly reflected in the management tool, which colour-coded all medications to reflect whether they could be prescribed by a doctor or a nurse or only by a doctor, and were also communicated to clinic managers by way of a circular from the district managers. The expanded prescribing provisions initially resulted in some tensions between nurses, doctors, and pharmacists. These were resolved through a facilitated group session and informal communication within clinics, sometimes involving the nurse trainer. This intervention was the only modification to the training during the trial.

#### Intervention monitoring

The integrity of the intervention was assessed in several ways. Nurse trainers were observed during the initial live-in course and at quarterly follow-up workshops. Two nurse trainers were interviewed, and, in December 2011, focus group discussions were held with nurses in four intervention clinics. Nurses representing both rural and small town locations were enthusiastic about the management tool and recognised that it was a new way of strengthening care for NCDs. In particular they appreciated the format and the standardised framework for providing routine care, and the familiar features shared with PALSA PLUS. Consistent with our previous experience with PALSA PLUS, some variation in uptake of the management tool by nurses was reported. There was a tendency for nurses who formerly used PALSA PLUS to adopt PC101 and use it regularly, whereas nurses who had not used PALSA PLUS were less likely to begin to use the new management tool routinely [11,12]. Uptake by the trainers was considered excellent, and trainers completed planned sessions in all intervention clinics, some repeating sessions to ensure coverage of most staff.

### Data Collection

Fieldworkers recruited from local communities were trained to collect the trial data. They invited patients seated in the waiting rooms to be considered for the study and screened them using a structured questionnaire. Patients who met the eligibility criteria (Table 1) and provided informed consent were enrolled in the trial and completed the baseline questionnaire in Afrikaans, isiXhosa, or English, administered by the fieldworker using a handheld electronic device. Anthropometry (weight, height, waist circumference) and blood pressure were recorded [47]. Patients were asked to attend a follow-up interview 14 mo after their baseline interview. The lengthy period between interviews was intended to allow adequate opportunity for health workers to intervene in the care of trial patients, given that chronic disease patients are seldom reviewed at clinics more often than every 3–6 mo.

The questionnaire included questions on medical history, smoking status, mental health, health-related quality of life, and socio-economic status. The severity of respiratory symptoms among patients in the respiratory cohort was assessed using the symptom and activity domains of the St George’s Respiratory Questionnaire [48]. Patients who chose to complete the interview in isiXhosa were excluded from this section of the interview as there is no tested isiXhosa translation of this instrument. The presence of symptoms of depression was assessed with the CESD-10, administered to all patients enrolled in the study [32].

Depression treatment was defined as having received counselling, having been referred to psychiatric services, or being on an antidepressant at a therapeutic dose. Low-dose amitriptyline and imipramine are widely prescribed in South Africa for pain management or insomnia. We therefore defined antidepressant use at a therapeutic dose as prescription of amitriptyline or imipramine ≥50 mg daily and/or any other antidepressant. Counselling was defined as “talking with someone in a way that helps to find solutions to problems, or receive emotional support, and not just receiving advice on how to take medication.”

Fieldworkers extracted and photocopied patients’ prescription charts from their folders, clinic stores, and pharmacies for the year preceding the baseline interview. The medically qualified trial manager (N. F.) analysed all prescription charts and recorded prescriptions of chronic medication for each patient at the time of their interview. A data capturer entered the prescription data (medication, dose, and frequency) into a database, and the total daily dose for each medication was calculated. Prescription, interview, and laboratory data were imported and stored in a SQL server database, and a single longitudinal record constructed for every patient by the study database scientist (V. T.).

Reminder letters and cell phone text messages were sent to patients in the month preceding their scheduled follow-up interview. Patients who failed to attend this appointment were traced by phone or home visit. Patients received a gift voucher for a local grocery store with a value of ZAR100 (US$12.25) on completion of the follow-up interview, to compensate for travel costs and time. The follow-up questionnaire was similar to the baseline questionnaire, and fieldworkers repeated the anthropometry and blood pressure measurements. At follow-up, prescription data for the period since baseline were extracted, photocopied, analysed, and documented in the same way as at baseline.

Quality control measures included supervision of fieldworkers, electronic alert messages for fieldworkers if unusually high or low values were entered into the electronic questionnaire, monitoring of the data to identify unusual values or trends, and double entry of prescription data. At follow-up, prescription data were queried if they were missing, if the date of the prescription fell outside of a 1-mo window period based on the scheduled re-interview date, or if cohort-specific medications were excluded.

Blinding of the intervention was not possible at the clinic level due to the nature of the intervention.

### Outcome Measures

The primary outcome for hypertension, diabetes, and chronic respiratory disease was treatment intensification, reflected by an increase in dose or number of medications or change in medication class. This outcome was chosen after considering research identifying clinician inertia as a key reason for failure to control these conditions [49,50]; treatment intensification is associated with improved control [51–53]; was likely appropriate for the study population, where under-treatment was highly prevalent [1,7,8]; fitted well with the focus of the intervention on the clinical practices of nurses and the expansion of their prescribing with training; and could be applied across three of the four chronic conditions of interest. Definitions of treatment intensification by cohort are summarised in Table 1. For the depression cohort, case detection was selected as the primary outcome because depression is recognised to be under-diagnosed and under-treated in primary care [54].

Secondary outcome measures were as follows: disaggregation of primary outcomes by type of medication; cardiovascular disease risk and risk factors such as blood pressure, body mass index (BMI), and smoking status; health-related quality of life measured using the EuroQol-5D [55] and the World Health Organization Disability Assessment Schedule 2.0 (WHODAS 2.0) [56]; mortality; and healthcare utilisation. These last four outcomes were designed to detect evidence of harm resulting from shifting clinical responsibility from doctors to nurses, an often overlooked consideration in evaluations of task-shifting [57].

### Sample Size and Statistical Power

The study was powered to detect clinically important differences in primary outcomes within each cohort, accounting for the cluster randomisation design. With 38 clinics available for randomisation, we calculated the number of patients needed per clinic for each cohort to detect differences in primary outcomes of between 10% and 15%, with 90% power, 5% significance, and intraclass correlations of outcome based on previous studies, and assuming 20% loss to follow-up (Table 1). Baseline rates of treatment intensification were not available in South Africa, and so we used rates from studies completed in high-income settings [50,58].

HbA1c was measured as part of the pre-planned blood sampling strategy in a subgroup of clinics because resource limitations meant that we could not measure it in all diabetic patients in all 38 clinics. We estimated that HbA1c tests were needed from 30 diabetic patients in 10 clinics in each group (i.e., 600 diabetic patients from 20 clinics in total) in order to a show a difference of 0.5% (HbA1c of 8.8% in the control group versus HbA1c of 8.3% in the intervention group, assuming a standard deviation of 3.4%).

### Analysis

We compared baseline clinic and patient characteristics between treatment groups. All clinics and patients were analysed in the treatment group to which they were randomly assigned. Primary and secondary outcomes were analysed at the patient level, separately within each cohort. No adjustment was made for the multiple disease-specific primary outcomes. The cluster randomisation design was accounted for using robust cluster variance-covariance estimates. Intervention effects were estimated using binomial regression models with treatment as the main effect, adjusted for stratification, and are reported with 95% confidence intervals. Secondary analyses were further adjusted for potentially confounding baseline characteristics such as treatment status and disease control at baseline, smoking status, age, sex, and co-morbidity with one of the study diseases.

We carried out pre-specified subgroup analyses of the primary outcomes stratified by baseline level of disease control using binomial regression models including baseline disease control as a covariate. Baseline disease control of hypertension was defined as blood pressure < 140/90 (or, in patients with diabetes or a history of cardiovascular disease, <130/80), and for diabetes, as HbA1c < 7%. For depression, since the outcome was detection, “control” was defined as any patient receiving treatment for depression as follows: being on antidepressant medication at therapeutic dosage or having received counselling in the past year or having been referred to psychiatric services in the last year. No definition of disease control was applied to patients with chronic respiratory disease. Heterogeneity of the intervention effect was assessed by looking at the interaction between treatment and baseline disease control. In addition, we pre-specified secondary analyses of the primary outcomes disaggregated by component. For the primary outcomes, missing data were considered not to have occurred.

We used linear regression to compare changes between baseline and follow-up in blood pressure, waist circumference, weight, BMI, HbA1c, and health status measures between the treatment groups, adjusted for stratification. Similarly, we used ordinal logistic regression to compare readiness to quit smoking, and Poisson regression to compare rates of healthcare utilisation between the treatment groups. Stata version 13.0 statistical software was used for all analyses.

## Results

**Fig 1:**
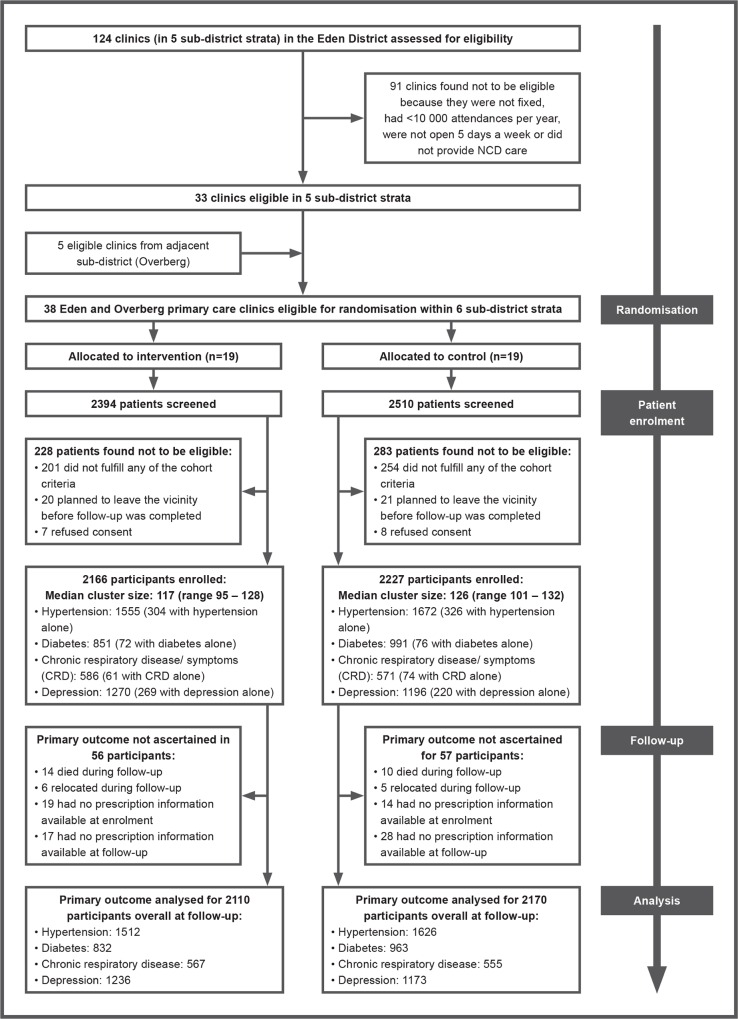
Trial profile. NCD, non-communicable disease.

Fig 1 shows the trial profile. All 38 randomised clinics completed the trial. In all, 4,904 patients were screened, of whom 4,393 patients met the eligibility criteria and were enrolled in the trial. Recruitment targets were exceeded for all cohorts except for diabetes, where recruitment fell short of targets. Enrolment of patients took place between 28 March 2011 and 10 November 2011 and was completed in intervention clinics before educational outreach sessions to nurses began. Follow-up data collection began on 21 May 2012 and ended on 13 December 2012.

In all, 1,927 patients in the intervention group were interviewed at follow-up (1,927/2,166; 89%), and 2,050 in the control group (2,050/2,227; 92%). Reasons for not being re-interviewed were similar between groups: death (63 in the intervention group versus 54 in the control group); relocation (42 in the intervention group versus 26 in the control group); too ill to be re-interviewed (two in the intervention group versus zero in the control group); and could not be traced (132 in the intervention group versus 97 in the control group). Prescription charts could be traced, and thus the primary outcome ascertained, for 206 patients who were not re-interviewed in the intervention group, and 151 in the control group, accounting for the very high rates of patients contributing data to the primary endpoint analysis (Fig 1).

Baseline patient characteristics are presented in Table 2 and detailed in a separate publication [47]. Baseline clinic characteristics are provided in Table A in S1 Appendix. Intervention and control clinics had similar numbers of nurses and doctors. Control clinics tended to be larger and, by chance, had more psychiatric services and on-site pharmacy facilities.

**Table 2:** Characteristics of patients allocated to an educational outreach programme (intervention group) or no new training (control group).

Characteristic	Intervention	Control
Patients recruited	2,166 (49)	2,227 (51)
Women	1,573 (73)	1,621 (73)
Age (years): median (IQR)	51 (42–61)	53 (44–62)
Language selected for the interview		
• Afrikaans	1,794 (83)	1,885 (85)
• isiXhosa	145 (7)	192 (9)
• English	227 (11)	150 (7)
Highest education level achieved		
• Tertiary education	40 (2)	35 (2)
• Secondary school education	923 (43)	930 (42)
• Primary school education	818 (38)	940 (42)
• No schooling	146 (7)	145 (7)
• Not obtained	239 (11)	177 (8)
Employed or self-employed	557 (26)	531 (24)
Receiving a social government grant	1,205 (56)	1,323 (59)
Housing density^1^: median (IQR)	2 (1–2), *n* = 1,426	2 (1–2), *n* = 1,505
Multimorbidity		
• Hypertension only	304 (14)	326 (15)
• Diabetes only	72 (3)	76 (3)
• Chronic respiratory disease only	61 (3)	74 (3)
• Depression only	269 (12)	220 (10)
• Two conditions	911 (42)	949 (43)
• Three or four conditions	549 (25)	582 (26)
Past medical history		
• Known cardiovascular disease (heart attack, angina, stroke)	605 (28)	505 (23)
• Previous tuberculosis	237 (11)	255 (12)
• History of hypertension	1,590 (73)	1,718 (77)
• History of diabetes	854 (39)	998 (45)
• History of depression	525 (24)	558 (25)
Smoking history		
• Current	652 (30)	731 (33)
• Past	464 (21)	550 (25)
• Never	1,022 (47)	921 (41)
• Not obtained	28 (1)	25 (1)
Pack-year history for current and ex-smokers: median (IQR)	7 (3–15), *n* = 869	7 (3–13), *n* = 1,064
Hospitalisation in 3-mo period preceding interview	134 (6)	136 (6)
BP ≥ 140/90 mm Hg^2^	1,055 (49)	1,216 (55)
BP ≥ 180/110 mm Hg^2^	166 (8)	193 (8)
Weight (kg): mean (SD)	77 (20), *n* = 2,111	77 (19), *n* = 2,179
BMI (kg/m^2^): mean (SD)	30 (8), *n* = 2,060	30 (8), *n* = 2,104
Obese (BMI ≥ 30 kg/m^2^)	972 (45)	1,008 (45)
Waist circumference (cm): mean (SD)	98 (16), *n* = 2,140	98 (16), *n* = 2,205
Waist circumference more than ideal^2^	1,316 (61)	1,381 (62)
10-y non-laboratory-based cardiovascular disease death risk (percent)^3^: mean (SD)	22 (20), *n* = 1,335	26 (21), *n* = 1,327
HbA1c (percent): mean (range), median (IQR)	9 (4–17), 8 (7–10), *n* = 310	9 (5–17), 9 (7–11), *n* = 394
HbA1c ≥ 7%	227 (73), *n* = 310	317 (81), *n* = 394

Values are *n* (percent) unless stated otherwise.

^1^Housing density: number of occupants/number of rooms.

^2^Waist circumference >88 cm for women, >104 cm for men.

^3^Ten-year risk of cardiovascular disease death (sudden cardiac or stroke death). Score calculated for patients with no known cardiovascular disease.

BMI, body mass index; BP, blood pressure; IQR, interquartile range; SD, standard deviation.

Baseline patient characteristics were generally well balanced between arms. Seventy-three percent of patients were women, and the median age was 52 y. There were high levels of unemployment and receipt of social welfare grants. Multimorbidity was common: 42% of patients had two conditions, and 26% more than two. The percentage of patients with a single condition of interest was as follows: hypertension, 20% (630 of 3,227); depression, 20% (489 of 2,466); diabetes, 8% (148 of 1,842); and chronic respiratory disease, 12% (135 of 1,157). A quarter of patients reported established cardiovascular disease. Eleven percent reported previous TB, and 2% reported being on ART. There were signs of under-treatment and under-diagnosis, with 18% of hypertensive patients reporting no or only one current antihypertensive medication, only 51% of diabetic patients receiving statins, only 50% of those with chronic respiratory disease or symptoms receiving any respiratory medication, and only 25% of those who screened positive for depression reporting some form of relevant treatment for the condition.

There was poor control of hypertension and diabetes despite treatment: blood pressure was ≥140/90 mm Hg in 59% of hypertensive patients, and HbA1c was ≥7% in 77% of those with diabetes in whom HbA1c was measured at baseline (704/1,842; 38%).

Treatment intensification in the hypertension and diabetes cohorts across both the intervention and control groups was common during the study period (Table 3), slightly favouring the intervention group (44% versus 40% for hypertension and 57% versus 50% for diabetes), although these differences were not significant when adjusted for stratification by sub-district and clustering. For hypertension, the risk ratio (RR) was 1.08 (95% CI 0.94 to 1.24; *p* = 0.252); for diabetes, the RR was 1.10 (95% CI 0.97 to 1.24; *p* = 0.126). Rates of treatment intensification in the chronic respiratory disease cohort were low (14% in the intervention group versus 12% in the control group) and not significantly different between groups (RR 1.08; 95% CI 0.75 to 1.55; *p* = 0.674). Fewer patients who screened positive for depression in the intervention group reported receiving treatment for depression at follow-up than their control group counterparts (18% versus 24%), but there was no difference between groups after adjustment for the trial’s design (RR 0.76; 95% CI 0.53 to 1.10; *p* = 0.142). Adjustment for baseline characteristics (Table 2) did not materially alter these results. The full regression models are presented in Table D in S1 Appendix.

**Table 3:** Primary outcomes for each disease cohort.

Disease Cohort	Outcome	Intervention, *n*/*N* (Percent)	Control, *n*/*N* (Percent)	Effect Estimate: Risk Ratio				ICC
				Crude Model		Adjusted Model		
				Estimate (95% CI)	*p-*Value	Estimate (95% CI)	*p-*Value	
Hypertension	Treatment intensification	685/1,555 (44)	673/1,672 (40)	1.08 (0.94 to 1.24)	0.252	1.10^1^ (0.96 to 1.27)	0.165	0.043
Diabetes	Treatment intensification	481/851 (57)	498/991 (50)	1.10 (0.97 to 1.24)	0.126	1.11^2^ (0.99 to 1.26)	0.083	0.030
Chronic respiratory disease	Treatment intensification	81/586 (14)	68/571 (12)	1.08 (0.75 to 1.55)	0.674	1.22^3^ (0.88 to 1.68)	0.228	0.011
Depression	Case detection	224/1,253 (18)	283/1,186 (24)	0.76 (0.53 to 1.10)	0.142	0.80^4^ (0.57 to 1.10)	0.167	0.077

^1^Adjusted for age, sex, body mass index, smoking status, diabetes, chronic respiratory disease, blood pressure control, maximal medical therapy at baseline, history of cardiovascular disease.

^**2**^Adjusted for sex, body mass index, smoking status, hypertension, history of cardiovascular disease at baseline.

^3^Adjusted for age, smoking status, diabetes, history of tuberculosis, whether or not receiving respiratory medication at baseline.

^4^Adjusted for sex, smoking status, hypertension, history of depression, 10-item Center for Epidemiologic Studies Depression Scale score at baseline, whether or not receiving antidepressant medication at baseline.

ICC, intraclass correlation coefficient.

Pre-specified subgroup analyses by baseline level of disease control (Table 4) showed that, in the diabetic cohort, the intervention was associated with treatment intensification only among patients with baseline HbA1c of 7%–10% (RR 1.30; 95% CI 1.16 to 1.47; *p-*value for interaction = 0.010). In the other cohorts, there were no significant differences in effectiveness between subgroups. However, treatment intensification tended to be more common, in both arms, in subgroups with poorer control at baseline. The non-significant difference in depression treatment, which favoured the control group, was mostly among those already receiving treatment for depression at baseline.

**Table 4:** Subgroup analyses: primary outcomes stratified by level of disease control at baseline.

Baseline Subgroup (Pre-specified)	Intervention, *n*/*N* (Percent)	Control, *n*/*N* (Percent)	Effect Estimate: Risk Ratio		WALD *p-*Value^1^
			Estimate (95% CI)	*p-*Value	
**Hypertension**					0.444
BP uncontrolled^2^	546/1,127 (49)	545/1,268 (43)	1.12 (0.97 to 1.28)	0.113	
BP controlled^2^	139/426 (33)	128/399 (32)	1.01 (0.76 to 1.33)	0.954	
**Diabetes**					0.010
HbA1c < 7%	34/83 (41)	29/77 (38)	1.08 (0.77 to 1.52)	0.638	
HbA1c 7%–10%	97/140 (69)	93/170 (55)	1.30 (1.16 to 1.47)	<0.001	
HbA1c > 10%	62/87 (71)	107/147 (73)	0.97 (0.81 to 1.16)	0.703	
**Chronic respiratory disease: symptom score subgroup**					0.532
SGRQ symptom score ≤ median	20/189 (11)	19/228 (8)	1.17 (0.66 to 2.07)	0.581	
SGRQ symptom core > median	37/221 (17)	35/195 (18)	0.95 (0.65 to 1.39)	0.802	
**Chronic respiratory disease: activity score subgroup**					0.693
SGRQ activity score ≤ median	36/256 (14)	34/273 (13)	1.07 (0.7 to 1.65)	0.744	
SGRQ activity score > median	40/271 (15)	31/254 (12)	1.21 (0.77 to 1.92)	0.412	
**Depression**					0.632
Receiving any treatment for depression^3^	76/278 (27)	127/336 (38)	0.74 (0.54 to 1.02)	0.063	
Not receiving any treatment for depression^3^	148/990 (15)	156/860 (18)	0.84 (0.49 to 1.42)	0.510	

^1^ *p-*Values for arm-subgroup interaction.

^2^BP uncontrolled defined as ≥130/80 mm Hg for patients with diabetes or a history of cardiovascular disease, and ≥140/90 mm Hg for all other patients.

^3^Receiving treatment for depression defined as being on antidepressant medication at therapeutic dosage or having received counselling in the past year or having been referred to psychiatric services in the last year.

BMI, body mass index; BP, blood pressure; SGRQ, St George’s Respiratory Questionnaire.

Disaggregated primary outcomes are presented in Table E in S1 Appendix. Notable findings include apparently significantly higher rates of aspirin initiation among patients with hypertension and diabetes attending intervention clinics, even though aspirin prescribing was restricted to doctors. Angiotensin-converting enzyme (ACE) inhibitor use was significantly higher among intervention group patients with known cardiovascular disease, as was sulphonylurea use among intervention group diabetic patients with BMI ≥ 30 kg/m^2^. In the depression cohort, the higher rate of depression treatment in the control arm was because more control group patients reported receiving counselling (15% in the intervention arm versus 22% in the control arm) and referral to psychiatric services (5% in the interventional arm versus 9% in the control arm). There was no significant difference between groups in the use of antidepressants, which was very low (<5%).

Table 5 reports differences in cardiovascular risk factors between baseline and follow-up. There were no differences between groups in terms of blood pressure, waist circumference, BMI, or HbA1c. Smoking quit rates were high overall, but similar between groups. However, readiness to quit smoking was significantly higher in the intervention group (odds ratio 1.73; 95% CI 1.17 to 2.57).

**Table 5:** Effect on cardiovascular disease risk and risk factors; all four cohorts pooled.

Risk/Risk Factor	Measurement at Follow-Up		Change between Baseline and Follow-Up					
	Intervention^1^	Control^1^	Intervention^1^	Control^1^	Effect Estimate		*p-*Value	ICC
					Type	Estimate (95% CI)		
CVD risk^2^	22.1 (20.0), *n* = 1,550	24.9 (20.6), *n* = 1,417	−0.4 (8), *n* = 1,365	−1.1 (8), *n* = 1,303	Diff in means	0.54 (−0.51 to 1.59)	0.310	0.038
SBP (mm Hg)	134 (23.0), *n* = 1,927	135 (21.7), *n* = 2,049	1.2 (21.8), *n* = 1,925	−1.1 (21.7), *n* = 2,044	Diff in means	2.00 (−0.87 to 4.87)	0.172	0.038
DBP (mm Hg)	88 (13.2), *n* = 1,927	87 (12.7), *n* = 2,049	0.0 (13.5), *n* = 1,925	−1.8 (13.4), *n* = 2,044	Diff in means	1.58 (−0.56 to 3.72)	0.148	0.058
Proportion with uncontrolled BP^3^	1,267/2,166 (58%)	1,325/2,227 (60%)	N/A	N/A	Risk ratio	1.02^4^ (0.96 to 1.09)	0.464	0.024
Waist circumference (cm)	98.3 (16.7), *n* = 1,886	99.6 (16.8), *n* = 1,998	0.3 (8.4), *n* = 1,867	0.8 (8.8), *n* = 1,981	Diff in means	−0.53 (−2.30 to 1.25)	0.563	0.131
Weight (kg)	77.2 (19.7), *n* = 1,872	77.2 (19.2), *n* = 1,992	−0.1 (6.5), *n* = 1,866	−0.3 (6.5), *n* = 1,985	Diff in means	0.15 (−0.52 to 0.82)	0.665	0.024
BMI (kg/m^2^)	30.1 (7.6), *n* = 1,866	30.5 (7.5), *n* = 1,981	0.0 (2.5), *n* = 1,863	−0.1 (2.6), *n* = 1,979	Diff in means	0.06 (−0.21 to 0.32)	0.672	0.024
HbA1c (percent)	9.1 (2.6), *n* = 285	9.5 (2.6), *n* = 333	0.0 (2.4), *n* = 161	−0.2 (2.1), *n* = 218	Diff in means	0.21 (−0.43 to 0.85)	0.508	0.055
Proportion who smoke	480/2,166 (22%)	577/2,227 (26%)	N/A	N/A	Risk ratio	0.88^5^ (0.74 to 1.06)	0.178	0.037
Proportion who quit smoking	167/574 (29%)	194/668 (29%)	N/A	N/A	Risk ratio	1.01 (0.71 to 1.42)	0.971	0.049
Number of units smoked per day	6.8 (6.1), *n* = 479	6.6 (5.1), *n* = 578	−0.7 (5.7), *n* = 406	−0.6 (5.7), *n* = 578	Diff in means	−0.08 (−1.07 to 0.91)	0.869	0.047
Readiness to quit smoking					Odds ratio	1.73 (1.17 to 2.57)	0.006	0.104
• Thinking of quitting in next 30 d	73/480 (15%)	66/577 (11%)	N/A	N/A				
• Thinking of quitting in next 6 mo	318/480 (66%)	337/577 (58%)	N/A	N/A				
• Not thinking of quitting	89/480 (19%)	174/577 (30%)	N/A	N/A				

^1^Mean (standard deviation) or *n/N* (percent).

^2^Ten-year risk of cardiovascular disease death (sudden cardiac or stroke death). Score calculated for patients with no known cardiovascular disease.

^3^Uncontrolled BP defined as ≥130/80 mm Hg for patients with diabetes or a history of cardiovascular disease, and ≥140/90 mm Hg for all other patients.

^4^Adjusted for uncontrolled BP at baseline, age, and sex.

^5^Adjusted for insulin at baseline, uncontrolled BP at baseline, BMI, sex, hypertension, and history of cardiovascular disease.

BMI, body mass index; BP, blood pressure; CVD, cardiovascular disease; Diff, difference; DBP, diastolic blood pressure; ICC, intraclass correlation coefficient; N/A, not applicable; SBP, systolic blood pressure.

There were no differences between groups in health outcomes measured with the EuroQol-5D [55], CESD-10 [32], or World Health Organization Disability Assessment Schedule 2.0 [56] (Table 6). Mortality did not differ between groups (Table 6). Healthcare utilisation, as measured by clinic visits and hospital admissions during the 3 mo before the follow-up visit, was similar between groups, but there was a statistically non-significant higher number of hospital admissions in the intervention group (Table 7).

**Table 6:** Effect on quality of life, depression, and mortality.

Outcome	Measurement at Follow-Up		Change between Baseline and Follow-Up					
	Intervention^1^	Control^1^	Intervention^1^	Control^1^	Effect Estimate		*p-*Value	ICC
					Type	Estimate (95% CI)		
EuroQol 5D index score^2^	0.8 (0.3), *n* = 1,927	0.8 (0.2), *n* = 2,050	0.0 (0.3), *n* = 1,924	0.0 (0.3), *n* = 2,045	Diff in means	0.00 (−0.05 to 0.06)	0.855	0.078
EuroQol 5D visual analogue scale^3^	75.1 (20.3), *n* = 1,927	74.0 (19.0), *n* = 2,050	12.1 (29.8), *n* = 1,924	6.4 (26.9), *n* = 2,045	Diff in means	6.06 (−3.25 to 15.36)	0.202	0.290
10-item Center for Epidemiologic Studies Depression Scale^4^	8.0 (6.3), *n* = 1,927	7.4 (6.1), *n* = 2,050	−3.1 (6.8), *n* = 1,926	−3.1 (7.3), *n* = 2,050	Diff in means	−0.12 (−1.72 to 1.48)	0.882	0.111
World Health Organization Disability Assessment Schedule 2.0^5^	17.1 (7.0), *n* = 1,740	17.6 (6.3), *n* = 1,933	N/A	N/A	Diff in means	−0.09 (−1.27 to 1.09)	0.878	0.113
Mortality	64/2,166 (3%)	64/2,227 (3%)	N/A	N/A	Risk ratio	1.11 (0.79 to 1.56)	0.564	0.003

^1^Mean (standard deviation) or *n/N* (percent).

^2^The EuroQol-5D index score is a weighted total between 0 and 1, where 0 = death and 1 = perfect health.

^3^The EuroQol-5D visual analogue scale is a score between 0 and 100 where 0 = worst imaginable state of health and 100 = best imaginable state of health.

^4^The 10-item Center for Epidemiologic Studies Depression Scale is scored from 0 to 30, with higher scores representing greater degrees of depressed mood.

^5^The World Health Organization Disability Assessment Schedule 2.0 is scored from 12 to 60, with higher scores representing greater degrees of disability.

Diff, difference; ICC, intraclass correlation coefficient; N/A, not applicable.

**Table 7:** Effect on healthcare utilisation.

Outcome	Intervention, Mean (SD)	Control, Mean (SD)	Effect Estimate			ICC
			Type	Estimate (95% CI)	*p-*Value	
Number of hospital admissions in 3 mo before follow-up interview	0.1 (0.4), *n* = 1,927	0.1 (0.3), *n* = 2,050	IRR	1.25 (0.91 to 1.71)	0.162	0.004
Number of inpatient days in 3 mo before follow-up interview	0.4 (2.8), *n* = 1,927	0.3 (2.4), *n* = 2,050	IRR	1.43 (0.83 to 2.48)	0.201	0.003
Number of clinic visits in 3 mo before follow-up interview	2.5 (1.7), *n* = 1,456	2.5 (1.4), *n* = 1,665	IRR	1.02 (0.93 to 1.13)	0.678	0.070

ICC, intraclass correlation coefficient; IRR, incidence rate ratio.

## Discussion

This paper reports our evaluation of the clinical effectiveness of a complex health systems intervention, based on task-shifting by adding nurse-led NCD and depression care to a proven effective, and scalable, integrated care model for nurse-led care of communicable diseases, in the context of limited availability of physicians to treat a high burden of multimorbid and poorly controlled NCDs in a middle-income country.

The primary analyses found no statistically significant effects of the intervention on the primary outcomes for any of the four disease cohorts. These cohorts were analysed separately, equivalent to four parallel trials; adjustment for having four primary outcomes instead of one would only have decreased statistical differences. Health status outcomes also did not differ between the intervention and control groups. But neither was there evidence of harm for any of these endpoints, or in terms of reduced well-being or excess hospitalisations or deaths. In addition, the intervention was not associated with higher healthcare utilisation at the primary care or hospital level. A pre-planned subgroup analysis by baseline level of diabetes control showed a benefit of the intervention in the subgroup of patients with moderately uncontrolled diabetes (HbA1c 7%–10% at baseline), but the two other pre-specified subgroup analyses (for hypertension and depression by baseline level of disease control) did not show a significant difference between groups.

While no primary outcomes showed a significant benefit of the intervention, the upper confidence limits included the possibility of meaningful clinical improvements, and the direction of results in three of the four primary endpoints in the study was consistent and positive. Also, the pre-specified secondary analysis of patients with diabetes and uncontrolled HbA1c measurements at baseline demonstrated a positive effect. After disaggregation of the disease groups, other significant findings were higher rates of aspirin initiation among patients with hypertension and diabetes, higher use of ACE inhibitors in patients with known cardiovascular disease, and more prescriptions of sulphonylureas in patients with diabetes and a high BMI (Table E in S1 Appendix).

The non-significant findings for the primary outcomes contrast with positive findings in our three previous pragmatic randomised controlled trials using a similar integrated management tool and the same training approach, focused on a narrower range of mainly communicable conditions [9–15,30,42,59]. These trials showed modest, but consistent, improvements across a range of process indicators and health and healthcare utilisation outcomes.

There are several potential reasons for the non-significant findings on the primary outcomes of our study. One is the level of uptake of PC101 into daily clinical practice. Owing to limited research funding, a complete and suitably detailed process evaluation of the uptake of PC101 into clinical practice was not possible. However, limited focus group discussions and observations in clinics by members of the research team confirmed heterogeneous uptake of PC101 within and between clinics, as might be expected in a pragmatic trial intervention. Overall low levels of uptake would seem unlikely, given the enthusiastic response and high uptake of the method by clinic staff reported in our previous implementation studies with the PALSA PLUS management tool [12,42]. Other factors should be considered, such as training. The addition of NCD care to the training programme may have proved a step too far—the content of the PC101 management tool was twice as substantial as that of the PALSA PLUS tool—and potentially overwhelming for nurses who were still learning to implement nurse-initiated and -managed antiretroviral treatment when the trial started. Furthermore, NCDs have long been managed by nurses in primary care clinics throughout South Africa, albeit with minimal training or intervention. As seen in the baseline characteristics, poor NCD care may have become entrenched, and markers of poor disease control routinely ignored [60]. The challenge of “undoing” these clinical habits and effecting a change in clinical behaviour is well described and may take repeated training sessions to achieve. Although training was provided throughout the trial, the comprehensive nature of PC101 made it difficult to cover the curriculum for NCDs sufficiently within the time frame of the study. Owing to limited research funding, but consistent with a pragmatic trial design, formal assessments of adequacy of training and uptake (use) of PC101 were not performed.

A further potential reason for the failure to show differences between groups was the effect of a co-intervention, the concurrent Chronic Disease Season campaign, instituted by the clinic managers in both control and intervention clinics. The impact of this unforeseen development is seen in the higher rates of treatment intensification for hypertension and diabetes (the focus of the campaign) than for chronic respiratory disease or depressive symptoms in both the intervention and control clinics. Whereas only 13% of patients with chronic respiratory disease and 3% of those with depression had medication intensified at follow-up, nearly half of those with hypertension and diabetes had intensified treatment (42% and 53%, respectively). These rates of intensification of antihypertensive and diabetic medications are similar to or slightly higher than those reported in high-income country settings [50,58].

Another consideration concerns methodology. We recruited all patients with the diseases of interest rather than only those requiring treatment intensification, and failed to assess adherence and exclude patients who did not adhere to previously prescribed medications and who might therefore have been less likely to have been prescribed additional treatment. However, the eligibility criteria were adopted on the assumption that decision-makers wanted evidence of effectiveness of the intervention across broad groups of patients, rather than for subgroups, and that, as lack of disease control was highly prevalent at baseline, the majority of patients would qualify for treatment intensification.

Other limitations of the study design include dependence on self-reported diagnoses for inclusion in the patient cohorts, reliance on process outcomes, and insufficient resources to measure important health outcomes, such as HbA1c, at follow-up. Also, the duration and timing of the follow-up data collection might not have been optimal for a study of chronic diseases, where follow-up visits being only every 3–6 mo limited opportunities for treatment intensification. This is illustrated by the low number of clinic visits during the follow-up period, a mean of around 2.5 per patient over a period of 14 mo (Table 7).

The main strength of the study was that it was a pragmatic trial, implemented under routine circumstances in a real-world setting with the intervention delivered by usual health department trainers, with minimal research-related distortions of care delivery. Observing this real-world implementation appears to have given relevant policy-makers sufficient confidence to make a decision on the suitability of the intervention for their health systems. Other strengths of the study include the cluster randomised design (appropriate to reduce the risk of contamination in an intervention directed at groups of nurses working in clinics), high follow-up rates for both patient interviews and prescription data, the inclusion of four different chronic diseases in a context characterised by high rates of multimorbidity, and identification and follow-up of patient participants by fieldworkers independent of clinical care.

So what are the implications of the trial for decision-makers in South Africa and other LMICs who are faced with overstretched health services and the need to address NCDs and mental health? In October 2013, even before the trial results were finalised, decision-makers were increasingly enthusiastic about the PC101 intervention, and both the Western Cape Department of Health and the National Department of Health in South Africa elected to commence implementation. Later dissemination of the trial findings on the effectiveness of this intervention to these local and national policy-makers did not change this decision. The decision, we were told, was much more influenced by demand from frontline clinicians and managers for what was perceived to be a highly feasible and acceptable approach to expanding skills for NCDs. Further factors that may have influenced decision-makers were the benefits of the new mode of clinician training reported in our prior studies [9,13,15], an independent report supporting the integrated Chronic Care Model as a feasible component of health system reform in South Africa [61], and the findings of a non-randomised evaluation of PC101 performed in 42 primary care clinics in three additional health districts [62]. The PC101 management tool is correctly seen as a means of overcoming the “silo” approach to individual disease management in which recommendations for different conditions may vary and even conflict and, more importantly, ensures that NCDs and mental health are not overlooked because of prioritisation of communicable diseases. For us, as researchers who look to rigorous research methods to guide health system development, this has been a powerful lesson in understanding that evidence of effectiveness is only one element under consideration by decision-makers [63]. Given clinicians’ strong attraction to the ease of integrating PC101 into clinic practice and the positive system effects of our intervention mentioned above, it might have been more useful to focus our primary analysis on lack of harm. For example, the study was not powered to test for differences in healthcare utilisation and reasons for referrals and hospitalisations. Thus, it is not possible to evaluate the significance of the small imbalance in numbers of hospital admissions between the intervention group and the control group, since an increase in hospitalisations reflecting more appropriate referrals from primary care may be interpreted as favourable rather than as a treatment failure. Specifically designed trials are required.

We now consider that it is our responsibility as health system researchers to invest in improving the effectiveness of this intervention. There are patterns in the data from the trial that provide reassurance that the intervention is not harmful and that, with further optimisation, might demonstrate improvements in effectiveness. Several adjustments have been made to the programme that is being scaled up with the aim of increasing its impact on skills, clinician confidence, and quality of care. The PC101 content has been broken down into four training modules (communicable diseases, NCDs, mental health, and women’s health) to allow staff to become familiar with one area at a time and embed changes into their clinical practice before moving to the next. We now also explicitly aim PC101 training at doctors, through dedicated workshops for professionals who would otherwise miss regular onsite training due to the sessional nature of their work. Implementation workshops, with an extra day aimed at meeting the needs of facility and middle managers, are included in the training of nurse trainers, and appointment of clinical governance teams within sub-districts allows local troubleshooting of barriers to implementation and inclusion of non-clinicians in the day-to-day running of the programme. A further cluster randomised trial in the North West province of South Africa (ClinicalTrials.gov NCT02407691) is currently evaluating the effect of the mental health module when combined with the provision of manualised depression counselling by lay health workers delivered to ART patients with co-morbid depression. A second study is evaluating this mental health module in patients with hypertension and co-morbid depression [64]. This expansion of human resources to include lay health workers is based on our experience from the PC101 trial that nurse training alone is insufficient to close the gap in depression care when there is limited access to treatment in the form of counselling services or antidepressant prescriptions (prescribing currently restricted to doctors).

Although it will not be possible to conduct another randomised controlled trial of the adapted PC101 implementation as it is scaled up, we plan to conduct such trials for future national and international adaptations of this programme [17]. Ease of implementability appears to be a major feature for policy-makers, and we will include proxies, such as acceptability to frontline clinicians, as outcome measures in future trials.

In conclusion, this pragmatic cluster randomised trial of the effects of an integrated management tool implemented using educational outreach to nurses showed no effect on treatment intensification in patients with NCDs or on case detection of depression. But neither was there evidence of harm. Despite this lack of positive clinical outcomes, decision-makers were disposed to view PC101 as a coherent, feasible, and acceptable extension of a programme of integrated care previously shown to be effective in the South African health system, and health authorities have committed to a national rollout of an improved version of the PC101 programme. The disjuncture between the clinical outcomes of our study and the policy choice exposes the different responsibilities of researchers and decision-makers in a health system. For us, as intervention developers, this focuses our attention on longer term improvements to strengthen components of the programme in order to achieve clinical impact on care for NCDs, while, as evaluators, we see the need for ongoing audit and further randomised pragmatic controlled trials to evaluate the effectiveness of these improvements.

Health systems research and development is an interactive and deliberative process. Perhaps the greatest contribution of this study lies in the relationships developed between our team and health system decision-makers, during a series of five large randomised evaluations of health systems interventions that responded to decision-maker-defined health systems needs over 16 years [17]. To this process we have each brought our different skills and perspectives, and together have developed, and are scaling up, an iteratively improved, evidence-informed approach to nurse-led primary care that strengthens human resources and health systems, and brings better care to South Africans, as well as models that can be applied in other low- and middle-income country settings.

## Supporting Information

S1 Appendix (DOCX)

S1 Data Dataset. (XLSX)

S1 Text Trial protocol. (DOCX)

S2 Text CONSORT checklist. (DOC)

S3 Text Template for Intervention Description and Replication (TIDieR) checklist. (DOCX)

S4 Text Baseline questionnaire. (PDF)

S5 Text Patient information sheet and consent form. (PDF)

## Abbreviations

ART: antiretroviral therapy
BMI: body mass index
CESD-10: 10-item Center for Epidemiologic Studies Depression Scale
COPD: chronic obstructive pulmonary disease
LMICs: low- and middle-income countries
NCD: non-communicable disease
PALSA PLUS: Practical Approach to Lung Health and HIV/AIDS in South Africa
PC101: Primary Care 101
RR: risk ratio
TB: tuberculosis
