# Supplementary material for: Educational Outreach with an Integrated Clinical Tool for Nurse-Led Non-communicable Chronic Disease Management in Primary Care in South Africa: A Pragmatic Cluster Randomised Controlled Trial
Source: PLoS Med. 2016 Nov 22;13(11):e1002178. doi: 10.1371/journal.pmed.1002178 (PMC5119726; doi:10.1371/journal.pmed.1002178)
Supplement: S1 Appendix — (DOCX) [file pmed.1002178.s001.docx]

**S1 Appendix**

**Table A** Characteristics of clinics allocated to an educational outreach programme (Primary Care 101) or no new training (control group). Values are numbers (percentages) unless stated otherwise.

| **Clinics** | **Intervention** | **Control** |
| --- | --- | --- |
| Number of clinics | 19 | 19 |
| Headcount^1^: median (IQR) | 19304 (16341-28064) | 30882 (20091-41053) |
| Setting: |  |  |
| Urban (%) | 11 (58) | 11 (58) |
| Peri-urban (%) | 4 (21) | 3 (16) |
| Rural (%) | 4 (21) | 5 (26) |
| Number of nurses per clinic: median (IQR) | 4 (3-5) | 6 (3-7) |
| Patient to nurse ratio (headcount/number of nurses): median (IQR) | 1:5552 (4329-9329) | 1:5835 (4412-8009) |
| Psychiatric qualified nurse available: |  |  |
| Weekly (%) | 6 (32) | 7 (37) |
| Monthly (%) | 10 (53) | 12 (63) |
| None (%) | 3 (16) | 0 (0) |
| Doctor support: |  |  |
| Daily (%) | 8 (42) | 7 (37) |
| Sessional (%) | 11 (58) | 12 (63) |
| Pharmacy on-site (%) | 6 (32) | 10 (53) |

^1^ Headcount: number of attendances of patients >5 years in 2008

**Table B:** Primary Care 101 Training Cases

| **Scenario** | **Patient name** | **Symptoms/ chronic diseases covered** | **Core learning** |
| --- | --- | --- | --- |
| 1 | Godfrey | Weight loss – diabetes screen | - Expansion of familiar page - Link with chronic condition screen |
| 2 | Patricia | Headache – stress | - Expansion of familiar page - Approach to stress |
| 3 | Auntie Gertie | Asthma: routine care | - Introduce routine care approach of ‘Assess, Advise, Treat’ |
| 4 | Godfrey | Tuberculosis (TB): routine care | - Approach to routine care for complicated chronic condition - Becoming familiar with new pages for TB |
| 5 | Stanley | Body pain – HIV | - Becoming familiar with new symptom page - Approach to routine care for complicated chronic condition - Becoming familiar with new pages for HIV |
| 6 | Thobeka | Back pain – cardiovascular disease (CVD) risk | - Using common symptom as trigger to screen for important chronic condition - Approach to assessing and managing CVD risk |
| 7 | Xolani | Face problems – stroke | - Diagnosing stroke - Approach to routine stroke care |
| 8 | Sipho | Chest pain – ischaemic heart disease (IHD) | - Managing the client needing urgent attention - Approach to routine IHD care |
| 9 | Jane | Fatigue – depression | - Identifying the client with depression - Diagnosing depression - Approach to routine depression care |
| 10 | Adelaide | Abdominal pain – substance abuse | - Identifying the client with substance abuse - Diagnosing substance abuse - Approach to routine substance abuse care |
| 11 | Faizel | Fits - epilepsy | - Managing the client needing urgent attention - Approach to routine epilepsy care |
| 12 | Melissa | HIV - pregnancy | - Look for other chronic condition in client with known chronic condition - Play with alternate scenarios for routine HIV care |
| 13 | Caroline | Diabetes - hypertension | - Using routine care approach with new chronic condition - Approach to routine diabetes care - Diagnosing hypertension in diabetes |
| 14 | Boeta | Joint symptom – gout, CVD risk, substance abuse | - Identifying the client with gout - Diagnosing gout - Approach to routine gout care - Identifying other chronic conditions in the client with 1 chronic condition and approach to routine care of client with several chronic conditions |

**Table C:** Expanded treatment patterns

| **Indication** | **Available for Practice Nurse (PN) prescription prior to Primary Care 101 (PC101) training** | **Available for PN prescription post PC101 training** |
| --- | --- | --- |
| Hypertension | Hydrochlorothiazide (HCTZ) | HCTZ *and*  Enalapril (maximum dose 10mg daily) *or*  Amlodipine (maximum dose 5mg daily) |
| Diabetes | Metformin | Metformin *and*  Glibenclamide (2.5mg daily to maximum 5mg twice daily (bd)) *or*  Gliclazide (40mg daily to maximum 80mg bd *and*  Simvastatin (maximum dose 10mg daily) *and*  Enalapril (maximum dose 10mg daily) if proteinuria |
| Cardiovascular disease or cardiovascular risk >20% | Aspirin | Simvastatin (maximum dose 10mg daily) |
| Asthma | Salbutamol inhaler  Budesonide for Clinical Nurse Practitioners (CNPs) | Salbutamol inhaler *and*  Budesonide (maximum dose 200mcg bd) *and*  Prednisone 40mg daily for 7 days (maximum 2 courses per year) for acute exacerbations |
| COPD | Salbutamol inhaler | Salbutamol inhaler *and*  Prednisone 40mg daily for 7 days (maximum 2 courses per year) for acute exacerbations |

**Table D:** Analysis of primary outcomes: risk ratios of the associations of arm and patient level characteristics

**Table D Hypertension (N=3227)**

| **Factor** | **n/N (%)** | **Risk ratio (95% CI)** | **P value** |
| --- | --- | --- | --- |
| ***Arm of trial*** |  |  |  |
| Control | 673/1672 (40) | 1 [reference] |  |
| Intervention | 685/1555 (44) | 1.10 (0.96 to 1.27) | 0.165 |
| ***Stratum*** |  |  |  |
| 1: Bitou and Knysna | 359/903 (40) | 1 [reference] |  |
| 2: Hessequa and Kannaland | 190/452 (42) | 1.02 (0.77 to 1.35) | 0.888 |
| 3: Eden DMA and Oudtshoorn | 223/615 (36) | 0.90 (0.67 to 1.21) | 0.485 |
| 4: George | 222/440 (51) | 1.19 (0.93 to 1.51) | 0.165 |
| 5: Mossel Bay | 206/395 (52) | 1.27 (1.05 to 1.53) | 0.012 |
| 6: Overberg | 158/422 (37) | 0.90 (0.72 to 1.13) | 0.371 |
| ***Patient characteristics*** |  |  |  |
| Age at enrolment^1^ |  | 1.00 (1.00 to 1.01) | 0.012 |
| ≤ 40 years | 135/379 (36) |  |  |
| 41-50 years | 320/780 (41) |  |  |
| 51-60 years | 458/1041 (44) |  |  |
| ≥ 61 years | 445/1027 (43) |  |  |
| Sex |  |  |  |
| Female | 1030/2425 (43) | 1 [reference] |  |
| Male | 328/802 (41) | 0.97 (0.87 to 1.08) | 0.595 |
| BMI^2^ |  |  |  |
| <=30 kg/m^2^ | 556/1438 (39) | 1 [reference] |  |
| >30 kg/m^2^ | 735/1628 (45) | 1.15 (1.05 to 1.25 | 0.001 |
| Smoking status |  |  |  |
| Never | 660/1519 (44) | 1 [reference] |  |
| Ex | 313/790 (40) | 0.95 (0.82 to 1.11) | 0.512 |
| Current | 374/885 (42) | 1.07 (0.97 to 1.17) | 0.160 |
| Diabetes |  |  |  |
| Not in diabetic cohort | 631/1687 (37) | 1 [reference] |  |
| In diabetic cohort | 727/1540 (47) | 1.16 (1.06 to 1.27) | 0.001 |
| Chronic respiratory disease (CRD) |  |  |  |
| Not in CRD cohort | 1061/2491 (43) | 1 [reference] |  |
| In CRD cohort | 297/736 (40) | 0.97 (0.88 to 1.07) | 0.525 |
| BP control^2^ |  |  |  |
| Controlled | 267/825 (32) | 1 [reference] |  |
| Not controlled | 1091/2395 (46) | 1.40 (1.20 to 1.63) | 0.000 |
| MMT at baseline^3^ |  |  |  |
| Not on MMT^3^ | 1167/2721 (43) | 1 [reference] |  |
| On MMT^3^ | 191/482 (40) | 0.87 (0.76 to 0.99) | 0.031 |
| History of CVD^2^ |  |  |  |
| No history of CVD^2^ | 1008/2378 (42) | 1 [reference] |  |
| History of CVD^2^ | 350/849 (41) | 0.96 (0.87 to 1.06) | 0.448 |

^1^Age at enrolment: presented as a categorical variable for descriptive purposes only

^2^BMI=body mass index; BP=blood pressure; CVD=cardiovascular disease

^3^MMT=maximal medical therapy: defined for hypertension as being on ≥ 3 antihypertensive drugs at optimal dosage

**Table D Diabetes n=1842**

| **Factor** | **n/N (%)** | **Risk ratio (95% CI)** | **P value** |
| --- | --- | --- | --- |
| ***Arm of trial: conditional on BMI***^1^ |  |  |  |
| BMI ≤ 30^1^ |  |  |  |
| Control | 202/404 (50) | 1 [reference] |  |
| Intervention | 158/327 (48) | 0.97 (0.81 to 1.15) | 0.717 |
| BMI >30^1^ |  |  |  |
| Control | 273/532 (51) | 1 [reference] |  |
| Intervention | 297/479 (62) | 1.20 (1.05 to 1.37) | 0.009 |
| ***Stratum*** |  |  |  |
| 1: Bitou and Knysna | 260/504 (52) | 1 [reference] |  |
| 2: Hessequa and Kannaland | 139/272 (51) | 0.99 (0.78 to 1.26) | 0.960 |
| 3: Eden DMA and Oudtshoorn | 137/327 (42) | 0.84 (0.59 to 1.18) | 0.308 |
| 4: George | 186/295 (63) | 1.21 (0.96 to 1.53) | 0.115 |
| 5: Mossel Bay | 127/199 (64) | 1.36 (1.09 to 1.71) | 0.006 |
| 6: Overberg | 130/245 (53) | 1.07 (0.86 to 1.32) | 0.549 |
| ***Patient characteristics*** |  |  |  |
| Sex |  |  |  |
| Female | 755/1382 (55) | 1 [reference] |  |
| Male | 224/460 (49) | 0.93 (0.82 to 1.04) | 0.200 |
| Smoking status |  |  |  |
| Never | 526/939 (56) | 1 [reference] |  |
| Ex | 240/473 (51) | 0.94 (0.84 to 1.04) | 0.226 |
| Current | 208/415 (50) | 0.95 (0.83 to 1.09) | 0.490 |
| History of CVD^1^ |  |  |  |
| No history of CVD^1^ | 777/1419 (55) | 1 [reference] |  |
| History of CVD^1^ | 202/423 (48) | 0.88 (0.80 to 0.98) | 0.019 |
| Hypertension (HPT) |  |  |  |
| Not in HPT cohort | 149/302 (49) | 1 [reference] |  |
| In HPT cohort | 830/1540 (54) | 1.08 (0.95 to 1.23) | 0.219 |

^1^BMI=body mass index; CVD=cardiovascular disease

**Table D Chronic Respiratory Disease (CRD) N=1157**

| **Factor** | **n/N (%)** | **Risk ratio (95% CI)** | **P value** |
| --- | --- | --- | --- |
| ***Arm of trial*** |  |  |  |
| Control | 68/571 (12) | 1 [reference] |  |
| Intervention | 81/586 (14) | 1.22 (0.88 to 1.68) | 0.228 |
| ***Stratum*** |  |  |  |
| 1: Bitou and Knysna | 31/303 (10) | 1 [reference] |  |
| 2: Hessequa and Kannaland | 22/166 (13) | 0.97 (0.50 to 1.89) | 0.930 |
| 3: Eden DMA and Oudtshoorn | 22/233 (9) | 0.81 (0.45 to 1.46) | 0.488 |
| 4: George | 33/155 (21) | 1.21 (0.79 to 1.87) | 0.379 |
| 5: Mossel Bay | 23/142 (16) | 1.20 (0.70 to 2.05) | 0.499 |
| 6: Overberg | 18/158 (11) | 0.84 (0.50 to 1.42) | 0.517 |
| ***Patient characteristics*** |  |  |  |
| Age |  |  |  |
| <=40 | 16/213 (8) | 1 [reference] |  |
| 41-60 | 96/692 (14) | 1.11 (0.75 to 1.64) | 0.618 |
| 61-80 | 36/242 (15) | 1.11 (0.72 to 1.71) | 0.652 |
| 81+ | 1/10 (10) | 0.80 (0.10 to 6.34) | 0.833 |
| Smoking status |  |  |  |
| Never | 39/394 (10) | 1 [reference] |  |
| Ex | 54/290 (19) | 1.49 (0.98 to 2.28) | 0.062 |
| Current | 55/454 (12) | 1.12 (0.74 to 1.69) | 0.601 |
| Diabetes |  |  |  |
| Not in diabetic cohort | 110/837 (13) | 1 [reference] |  |
| In diabetic cohort | 39/320 (12) | 0.80 (0.59 to 1.09) | 0.161 |
| History of tuberculosis |  |  |  |
| No history of tuberculosis at baseline | 117/943 (12) | 1 [reference] |  |
| History of tuberculosis at baseline | 31/211 (15) | 1.00 (0.67 to 1.49) | 0.994 |
| Chronic Respiratory Disease (CRD) medication |  |  |  |
| Not on CRD drugs at baseline | 27/567 (5) | 1 [reference] |  |
| On CRD drugs but not MMT^1^ | 81/231 (35) | 6.75 (4.11 to 11.09) | 0.000 |
| On CRD drugs at baseline and MMT^1^ | 41/346 (12) | 2.38 (1.34 to 4.24) | 0.003 |

^1^MMT=maximal medical therapy: defined for chronic respiratory disease as being on inhaled corticosteroid at a dose of ≥ 800mcg daily

**Table D Depression N=2439 (2466 in depression cohort but 27 patients excluded from depression primary analysis)**

| **Factor** | **n/N (%)** | **Risk ratio (95% CI)** | **P value** |
| --- | --- | --- | --- |
| ***Arm of trial*** |  |  |  |
| Control | 283/1186 (24) | 1 [reference] |  |
| Intervention | 224/1253 (18) | 0.80 (0.57 to 1.10) | 0.167 |
| ***Stratum*** |  |  |  |
| 1: Bitou and Knysna | 120/739 (16) | 1 [reference] |  |
| 2: Hessequa and Kannaland | 51/341 (15) | 0.82 (0.45 to 1.52) | 0.533 |
| 3: Eden DMA and Oudtshoorn | 108/458 (24) | 1.27 (0.62 to 2.59) | 0.507 |
| 4: George | 83/328 (25) | 1.13 (0.67 to 1.92) | 0.651 |
| 5: Mossel Bay | 69/273 (25) | 1.14 (0.65 to 2.03) | 0.644 |
| 6: Overberg | 76/300 (25) | 1.23 (0.70 to 2.16) | 0.464 |
| ***Patient characteristics*** |  |  |  |
| Sex |  |  |  |
| Female | 413/1859 (22) | 1 [reference] |  |
| Male | 94/580 (16) | 0.76 (0.62 to 0.94) | 0.011 |
| Smoking status |  |  |  |
| Never | 200/1011 (20) | 1 [reference] |  |
| Ex | 107/544 (20) | 0.96 (0.73 to 1.26) | 0.772 |
| Current | 193/848 (23) | 0.97 (0.78 to 1.20) | 0.764 |
| Hypertension (HPT) |  |  |  |
| Not in HPT cohort | 218/854 (26) | 1 [reference] |  |
| In HPT cohort | 289/1585 (18) | 0.72 (0.60 to 0.86) | 0.000 |
| History of depression |  |  |  |
| No history of depression | 233/1577 (15) | 1 [reference] |  |
| History of depression | 274/858 (32) | 1.73 (1.48 to 2.02) | 0.000 |
| CESD-10 score at baseline^1,2^ |  | 1.01 (0.99 to 1.04) | 0.197 |
| 10-15 | 257/1459 (18) |  |  |
| 16-20 | 145/672 (22) |  |  |
| 21-25 | 84/272 (31) |  |  |
| 26-30 | 21/63 (33) |  |  |
| Antidepressants at baseline at a therapeutic dose |  |  |  |
| Not receiving antidepressants | 384/2133 (18) | 1 [reference] |  |
| Receiving antidepressants | 119/292 (40) | 1.46 (1.12 to 1.89) | 0.004 |

^1^CESD-10= 10-item Centre for Epidemiologic Studies Scale

^2^ Presented as a categorical variable for descriptive purposes only

**Table E:** Primary outcomes disaggregated by components

**Table E Hypertension Cohort**

| **Outcome** | **Intervention** | **Control** | **Effect estimate** | | **P** | **Regression model** | **Adjusted for** |
| --- | --- | --- | --- | --- | --- | --- | --- |
|  | **n/N (%)** | **n/N (%)** | **Type** | **Estimate (95% CI)** |  |  |  |
| ***Disaggregation of primary outcome*** | | | | | | | |
| Treatment intensification of antihypertensive medication | 559/ 1555 (36) | 564/ 1672 (34) | RR | 1.07 (0.92 to 1.24) | 0.375 | Binomial | MMT, BP control, sex, diabetes, CVD^1,2^ |
| Addition of aspirin | 120/ 1555 (8) | 98/ 1672 (6) | RR | 1.44 (1.02 to 2.03) | 0.037 | Binomial | MMT, BP control, sex, diabetes, CVD^1,2^ |
| Addition or increase in the dose of a statin | 205/ 1555 (13) | 182/ 1672 (11) | RR | 1.27 (0.87 to 1.86) | 0.218 | Binomial | MMT, BP control, sex, diabetes, CVD^1,2^ |

^1^MMT=maximal medical therapy: defined for hypertension as being on ≥ 3 antihypertensive drugs at optimal dosage

^2^BP=blood pressure; CVD=cardiovascular disease

**Table E Diabetes Cohort**

| **Outcome** | **Intervention** | **Control** | **Effect estimate** | | **P** | **Regression model** | **Adjusted for** |
| --- | --- | --- | --- | --- | --- | --- | --- |
|  | **n/N (%)** | **n/N (%)** | **Type** | **Estimate (95% CI)** |  |  |  |
| ***Disaggregation of primary outcome*** | | | | | | | |
| Addition or increase in the dose of metformin | 156/ 851  (18) | 157/ 991  (16) | RR | 1.11 (0.83 to 1.48) | 0.472 | Binomial |  |
| Addition or increase in the dose of sulphonylurea | 127/ 851 (15) | 108/ 991  (11) | RR | 1.30 (0.98 to 1.73) | 0.074 | Binomial |  |
| Addition or increase in the dose of sulphonylurea if BMI ≤ 30 | 32/327 (10) | 47/404 (12) | RR | 0.87 (0.52 to 1.47) | 0.613 | Binomial | MMT, age, interaction between arm and BMI, sex, HPT, history of CVD^1,2^ |
| Addition or increase in the dose of sulphonylurea if BMI >30 | 85/479 (18) | 55/532 (10) | RR | 1.68 (1.23 to 2.30) | 0.001 | Binomial | MMT, age, interaction between arm and BMI, sex, HPT, history of CVD^1,2^ |
| Insulin | 189/851 (22) | 179/991 (18) | RR | 1.18 (0.92 to 1.51) | 0.194 | Binomial |  |
| Addition or increase in the dose of an ACE inhibitor | 96/ 851  (11) | 91/ 991  (9) | RR | 1.23 (0.88 to 1.72) | 0.223 | Binomial |  |
| Addition or increase in the dose of an ACE inhibitor if no history of CVD | 78/645 (12) | 81/774 (11) | RR | 1.13 (0.76 to 1.66) | 0.544 | Binomial | MMT, age, BMI, sex, HPT, interaction between arm and history of CVD^1,2^ |
| Addition or increase in the dose of an ACE inhibitor if history of CVD | 18/206 (9) | 10/217 (5) | RR | 2.76 (1.17 to 6.49) | 0.020 | Binomial | MMT, age, BMI, sex, HPT, interaction between arm and history of CVD^1,2^ |
| Addition of aspirin | 77/ 851  (9) | 60/ 991  (6) | RR | 1.73 (1.13 to 2.63) | 0.011 | Binomial |  |
|  |  |  |  | 1.70 (1.08 to 2.66) | 0.021 |  | MMT, BMI, age, sex, HPT, CVD^1,2^ |
| Addition or increase in the dose of a statin | 156/851  (18) | 154/ 991  (16) | RR | 1.19 (0.80 to 1.78) | 0.395 | Binomial |  |
|  |  |  |  | 1.15 (0.76 to 1.75) | 0.505 |  | MMT, age, BMI, sex, HPT, history of CVD^1,2^ |

^1^MMT=maximal medical therapy: defined for diabetes as being on insulin

^2^BMI=body mass index; HPT=hypertension; CVD=cardiovascular disease

**Table E Chronic Respiratory Disease Cohort**

| **Outcome** | **Intervention** | **Control** | **Effect estimate** | | **P** | **Regression model** | **Adjusted for** |
| --- | --- | --- | --- | --- | --- | --- | --- |
|  | **n/N (%)** | **n/N (%)** | **Type** | **Estimate (95% CI)** |  |  |  |
| ***Disaggregation of primary outcome*** | | | | | | | |
| Addition or increase in dose of inhaled corticosteroid | 55/586 (9) | 42/571 (7) | RR | 1.12 (0.72 to 1.73) | 0.608 | Binomial |  |
| Increase in dose or addition of inhaled corticosteroid |  |  | RR | 1.05 (0.65 to 1.67) | 0.854 |  | MMT, CRD drugs, SGRQACT, age, smoking, sex, HPT cohort^1,2^ |
| Addition of beta agonist | 13/586 (2) | 13/571 (2) | RR | 0.90 (0.51 to 1.56) | 0.697 | Binomial |  |
| Addition of beta agonist |  |  | RR | 0.78 (0.39 to 1.56) | 0.482 | Binomial | MMT, CRD drugs, SGRQACT, age, smoking, sex, HPT cohort^1,2^ |
| Addition of ipratropium bromide | 13/586 (2) | 11/571 (2) | RR | 1.22 (0.66 to 2.23) | 0.526 | Binomial |  |
| Addition of ipratropium bromide |  |  | RR | 1.27 (0.66 to 2.44) | 0.479 | Binomial | MMT, CRD drugs, SGRQACT, age, smoking, sex, HPT cohort^1,2^ |
| Addition of theophylline | 19/586 (3) | 20/571 (4) | RR | 0.97 (0.48 to 1.99) | 0.943 | Binomial |  |
| Addition of theophylline |  |  | RR | 1.21 (0.68 to 2.15) | 0.517 | Binomial | MMT, CRD drugs, SGRQACT, age, smoking, sex, HPT cohort^1,2^ |

^1^MMT=maximal medical therapy: defined for chronic respiratory disease as being on inhaled corticosteroid at a dose of ≥ 800mcg daily

^2^CRD=chronic respiratory disease; SGRQACT=St Georges Respiratory Questionnaire activity Domain; HPT=hypertension

**Table E Depression Cohort**

| **Outcome** | **Intervention** | **Control** | **Effect estimate** | | **P** | **Regression model** | **Adjusted for** |
| --- | --- | --- | --- | --- | --- | --- | --- |
|  | **n/N (%)** | **n/N (%)** | **Type** | **Estimate (95% CI)** |  |  |  |
| ***Disaggregation of primary outcome*** | | | | | | | |
| Addition or increase in antidepressant in therapeutic dosages | 41/ 1270  (3) | 36/ 1196  (3) | RR | 1.07 (0.69 to 1.65) | 0.773 | Binomial |  |
|  |  |  |  | 1.15 (0.77 to 1.73) | 0.500 |  | Psychiatric sister, age, history of depression, hypertension, sex, baseline antidepressant, diabetes |
| Received counselling | 194/ 1270 (15) | 264/ 1196  (22) | RR | 0.74 (0.49 to 1.12) | 0.153 | Binomial |  |
|  |  |  | RR | 0.01 (0.01 to 0.02) | 0.000 | Binomial | Age, HPT, diabetes, sex, smoking, counselling at baseline^1^ |
| Referral to mental health services | 67/ 1270  (5) | 108/ 1196  (9) | RR | 0.62 (0.41 to 0.93) | 0.022 | Binomial |  |
|  |  |  |  | 0.64 (0.45 to 0.92) | 0.015 |  | Psychiatric referral at baseline, sex, diabetes, smoking |

^1^HPT=hypertension
