# Supplementary material for: Educational Outreach with an Integrated Clinical Tool for Nurse-Led Non-communicable Chronic Disease Management in Primary Care in South Africa: A Pragmatic Cluster Randomised Controlled Trial
Source: PLoS Med. 2016 Nov 22;13(11):e1002178. doi: 10.1371/journal.pmed.1002178 (PMC5119726; doi:10.1371/journal.pmed.1002178)
Supplement: S1 Text — (DOCX) [file pmed.1002178.s003.docx]

Development and Evaluation of Tools to Manage Chronic Non-Communicable Diseases

Proposal to the Human Research Ethics Committee, University of Cape Town

Revised March 2012

Document History

| Date | Description | Detail |
| --- | --- | --- |
| March 2010 | Original submission to UCT Human Research Ethics Committee | Funded NHLBI proposal configured in UCT Ethics format |
| December 2010 | Revision submitted to UCT Human Research Ethics Committee | Concerns raised by first review addressed  Revised sample sizes as number of facilities available for trial now 37  Self-Reporting Questionnaire 20 (SRQ- 20) replaced by Centre for Epidemiologic Studies Depression Scale (CESD-10) |
| January 2011 | Revision submitted to UCT Human Research Ethics Committee | Patient information sheet revised following December 2010 piloting to improve clarity.  Brief patient information leaflet added. |
| March 2012 | Revision submitted to UCT Human Research Ethics Committee | Referral of patients with undiagnosed hypertension at follow up.  Referral of patients with end of life thoughts at follow up.  Addition of secondary outcomes and secondary analyses.  No passive dissemination of the PC101 guideline. |

## Contents

[Investigators 3](#_Toc255243334)

[Synopsis 4](#_Toc255243335)

[Abbreviations and acronyms 7](#_Toc255243336)

[Background 9](#_Toc255243337)

[Development and validation of a total cardiovascular risk assessment tool for developing countries 13](#_Toc255243339)

[Integrated Care Guideline Trial 16](#_Toc255243340)

[Economic Evaluation Model 21](#_Toc255243350)

[Recruitment and enrollment 25](#_Toc255243360)

[Research procedures and data collection 27](#_Toc255243361)

[Data safety and monitoring plan 28](#_Toc255243362)

[Data analysis 29](#_Toc255243363)

[Description of risks and benefits 30](#_Toc255243364)

[Potential risks and protection from risk 30](#_Toc255243365)

[Significance and potential benefit of research to participants and others 30](#_Toc255243366)

[Informed consent 31](#_Toc255243367)

[Privacy and confidentiality 31](#_Toc255243368)

[Reimbursement 31](#_Toc255243369)

[References 32](#_Toc255243370)

[Appendix 1: List of participating facilities 38](#_Toc255243374)

[Appendix 2: Referral letter for very high blood pressure 39](#_Toc255243375)

[Appendix 3: Referral letter for undiagnosed hypertension](#_Toc255243376) 40

[Appendix 4: Referral letter for participants visibly distressed emotionally and/or with suicidal ideation](#_Toc255243377) 41

Appendix 5: Referral letter for high triglyceride…………………………………………………………………………43

Appendix 6: Patient information sheet/ consent form………………………………………………………………….44

## Investigators

**Principal Investigators**

- Prof Dinky Levitt, Division of Diabetic Medicine and Endocrinology, Department of Medicine, University of Cape Town (overall proposal)
- Prof Tom Gaziano, Harvard Initiative for Global Health’s Cardiovascular Disease Working Group, Cardiovascular Medicine, Brigham & Women's Hospital, Boston (validation study and economic model)
- Dr Lara Fairall, Knowledge Translation Unit, University of Cape Town Lung Institute (trial)

**Co-investigators**

- Prof Krisela Steyn, Department of Medicine, University of Cape Town
- Prof Eric Bateman, Department of Medicine and Lung Institute, University of Cape Town
- Prof Alan Bryer, Department of Medicine, University of Cape Town
- Prof Debbie Bradshaw, Burden of Disease Research Unit, Medical Research Council
- Prof Carl Lombard, Biostatistics Unit, Medical Research Council
- Prof Max Bachmann, School of Medicine, Health Policy and Practice, University of East Anglia, Norwich, UK
- Prof Merrick Zwarenstein, Centre for Health Services Sciences, Sunnybrook Research Institute, Toronto, Canada
- Dr Beverly Draper, Knowledge Translation Unit, University of Cape Town Lung Institute
- Dr Ruth Cornick, Knowledge Translation Unit, University of Cape Town Lung Institute
- Prof Crick Lund, Department of Psychiatry and Mental Health, University of Cape Town
- Dr Naomi Folb, Knowledge Translation Unit, University of Cape Town Lung Institute
- Ms Serena van Haght, Chronic Disease Initiative in Africa, Department of Medicine, University of Cape Town Lung Institute
- Dr Simon Lewin, Norwegian Knowledge Centre for Health Services
- Dr Chris Colvin, Centre for Infectious Disease Epidemiology and Research, School of Public Health and Family Medicine, University of Cape Town
- Mr Ed Wilson, Health Economics Group, Faculty of Health, University of East Anglia, Norwich, UK

## Synopsis

The research programme described here is concerned with the identification and optimal management of chronic diseases and their risk factors in underserved communities. It comprises three inter-related component projects:

1. A validation study of a non-laboratory based tool to predict risk of future cardiovascular events (strokes, heart attacks, death)
2. A trial testing the effectiveness of a novel training approach to the management of chronic diseases by primary care nurses and doctors
3. Development of an economic model that can estimate the cost-effectiveness of interventions for chronic cardiovascular diseases

The reason these studies are presented together is because their research procedures and data collection are shared. Participants will be recruited from among adults attending 38 clinics in the Eden and Overberg districts of the Western Cape. The majority of patients seeking care from these public sector clinics are poor and from the underserved sectors of the South African population. Of this group of possible study participants the majority will either be people of African descent (speaking predominantly IsiXhosa) or people of mixed race ancestry (speaking predominantly Afrikaans).

Although there are differences in inclusion and exclusion criteria across the component studies, we anticipate a large proportion of participants will contribute data to more than one study. Participants will either be enrolled in the validation study or the trial, or both. Once validated, the non-laboratory based tool will be included in future versions of the guideline. Estimates of the guideline’s effectiveness, and information on patients’ health states and costs of care will be used in the economic model.

**Purpose/ design**

1. Validation study

Design: A cross-sectional study followed by a mortality outcome study

Purpose: To calibrate and validate a non-laboratory based screening tool for cardiovascular disease (CVD) risk prediction in South Africa

1. Trial

Design: Pragmatic (real-world) cluster (clinic) randomized controlled trial

Purpose: To test whether a training programme based on a concise 101-page guideline (Primary Care 101) improves the quality of care for chronic diseases over and above usual training and support programmes for these diseases. The trial will test whether the approach, previously shown to be effective for respiratory diseases including tuberculosis and HIV/AIDS, will remain effective when expanded to include the primary care management of other chronic diseases (hypertension, diabetes, depression).

1. Economic model

Design: Economic modeling study

Purpose: To develop a decision analytic model of cardiovascular disease (CVD) that would allow us to predict CVD events accurately to be used in a cost-effectiveness analysis comparing the use of different screening and intervention strategies.

**Inclusion and exclusion criteria**

These are summarised in the table. Participation is restricted to adults ≥18 years old, and to those capable of actively engaging in an interviewer-administered questionnaire at the time of recruitment, and 12-15 months later. As a result, potential participants who would be considered vulnerable by virtue of their age (too young), cognitive function or severe co-morbid illness are excluded.

**Participant (patient) revised inclusion and exclusion criteria**

| **Study** | **Validation study** | **Trial** |
| --- | --- | --- |
| Inclusion criteria | - Age ≥ 35 years *and* - Written consent to participate in the study - (Residence in the clinic vicinity for at least the previous year removed as an inclusion criterion). | - Age ≥ 18 years *and* - Planning to reside in the area for the next year *and* - Written consent to participate in the study *and* - Self-reported diabetes on treatment *or* - Self-reported hypertension on treatment *or* - Self-reported asthma/ chronic bronchitis/ emphysema on treatment *or* - Cough/difficult breathing > 2 weeks (and not on treatment for tuberculosis in the past 3 months) *or* - The Center for Epidemiologic Studies Depression Scale (CES-D 10 Scale) score of 10 or more |
| Exclusion criteria | - Prior cardiovascular event (e.g. stroke, myocardial infarct) - Acute and/or terminal condition precluding participation such as AIDS or cancer - Psychiatric diagnoses precluding participation such as schizophrenia, dementia and other cognitive impairment measured by self-reported or medical history. - Unavailable or no South African identity number | - Acute and/or terminal condition precluding participation such as AIDS or cancer - Psychiatric diagnoses precluding participation such as schizophrenia, dementia and other cognitive impairment measured by self-reported or medical history. |

**Recruitment, enrollment and data collection**

Trained fieldworkers will invite patients 18 years or older in the waiting room to participate in the study *after* their clinical consultation, and provide them with an information sheet, allowing them time to consider and discuss possible participation. Interested patients will be screened after consultation with the nurse/doctor, and in privacy, in an area of the clinic temporarily allocated to research staff.

Eligible participants who provide consent will be asked to undergo the following research procedures: measurement of their blood pressure, waist circumference, hip circumference, height, weight and an interview to determine: care received at the clinic, symptom severity, impact on their quality of life, healthcare utilisation, and the costs incurred by illness. Participants will also be asked to provide their South African identity number and any hospital folder numbers if available, to permit linkage with the national mortality register and hospitalisation databases respectively, thereby permitting long-term (5 years or more) follow-up.

In around 1/3 of participants, blood will be drawn to determine blood lipids (fats) and a marker of control of diabetes (HbA1C). Participants who qualified for the trial will be asked to return to the clinic 12-15 months later for a follow-up interview. An incentive to the value of R100 in the form of a voucher for a local grocery shop, will be provided to those participants who return for the follow-up interview. Participants who incur significant travel costs to return to the clinic for the follow-up interview will be reimbursed accordingly.

**Potential harms and measures to protect from harm**

The risks of these studies to the participants are minimal. The usual care will in no way be compromised if a patient chooses not to participate. Research procedures will take place after patients have received care at the clinic. The trial is not a Phase III trial where we are testing new treatments for chronic diseases but rather whether outcomes can be improved through the provision of a guideline-based training programme targeting nurses and doctors. The guideline is based on the best available evidence and is consistent with national recommendations. Care will be provided by experienced health practitioners. The Western Cape Department of Health will use the results of the trial to guide further implementation.

The following participants will be referred back to clinical staff for further assessment on the day of their interview (at baseline and follow up):

1. Participants with very high blood pressure (≥180/110) as they are at increased risk for stroke or heart attack.
2. Participants who become visibly emotionally distressed when answering the mental health component of the questionnaire

The following participants will be referred back to clinical staff for further assessment on the day of their interview at follow up only:

1. Participants with undiagnosed hypertension (blood pressure ≥140/90)
2. Participants who answer ‘yes’ to the following question, which has been added to the follow up questionnaire: Has the thought of ending your life been on your mind’.

An independent safety officer will review all blood results, and if safety thresholds are exceeded, contact clinic staff to ensure the participant is recalled, and action taken.

There is also minor risk of discomfort, bruising and bleeding during or after blood is drawn. This will be minimised by using only registered health practitioners to perform this procedure.

Confidentiality of data will be ensured by using computers for data entry. Once entered data may not be reviewed, and there are no paper questionnaires that could be lost or left in view of others. Uploaded questionnaires will be stored on a secure server. Access to identifiers will be restricted to a few key study staff for the purposes of data linkage with other datasets, and quality assurance. Only anonymised extracts will be prepared for analysis by study statisticians and researchers.

## Abbreviations and acronyms

| ACE | Angiotensin Converting Enzyme |
| --- | --- |
| Afro-E | East African |
| AHQ | Adult Health Questionnaire |
| AIDS | Acquired Immune Deficiency Syndrome |
| AMMP | Adult Morbidity and Mortality Project |
| ART | Antiretroviral Treatment |
| ATP | Adult Treatment Panel |
| BMI | Body Mass Index |
| BOD | Burden of Disease |
| BP | Blood Pressure |
| C/E | Cost Effective |
| CABG | Coronary Artery Bypass Grafting |
| CAD | Coronary Artery Disease |
| CD | Chronic Diseases |
| CDIA | Chronic Disease Initiative in Africa |
| CDL | Chronic Diseases of Lifestyle |
| CEA | Cost Effectiveness Analysis |
| CHC | Community Health Centre |
| CHD | Coronary Heart Disease |
| CHDPM | Coronary Heart Disease Policy Model |
| CHW | Community Health Worker |
| CITI | Collaborative IRB Training Initiative |
| CoE | Centre of Excellence |
| COPD | Chronic Obstructive Pulmonary Disease |
| CVD | Cardiovascular Disease |
| CVPD | Cardiovascular Pulmonary Disease |
| DALY(s) | Disability Adjusted Life Year |
| DANIDA | Danish International Development Agency |
| DCP2 | Disease Con troll Priorities Project in Developing Countries |
| DFID | Department for International Development |
| DHS | Demographic and Health Survey |
| DHS | District Health System |
| DM | Department of Medicine |
| DoH | Department of Health |
| ECG | Electrocardiograph |
| GDP | Gross Domestic Product |
| GEE | Generalised Estimating Equations |
| GRLS | Generalised Record Linkage System |
| GRME | Clinical Practice Guidelines and Research Methods and Ethics |
| HbA1C | Glycosylated Haemoglobin A1C |
| HDL | High Density Lipoprotein |
| HDLC | High Density Lipoprotein Cholesterol |
| HIV | Human Immuno-Deficiency Virus |
| Hr | Hour |
| ICC | Intracluster Correlation Co-efficient |
| ICCC | Innovative Care for Chronic Conditions |
| ID | Identification |
| IDRC | International Development Research Centre |
| IMAI | Integrated Management of Adult and Adolescent Illness |
| IMCI | Integrated Management of Childhood Illness |
| Inc | Incorporated |
| IRB | Institutional Review Board |
| ISH | International Society for Hypertension |
| ISPOR | International Society for Pharmacoeconomics and Operations Research |
| JNC | Joint National Committee |
| KTU | Knowledge Translation Unit |
| LDL | Low Density Lipoprotein |
| LMIC | Low and Middle Income Country |
| MI | Myocardial Infarction |
| MRC | Medical Research Council |
| NCEP | National Cholesterol Education Program |
| NEPAD | New Partnership for Africa’s Development Health Desk |
| NHBPEP | National High Blood Pressure Education Program |
| NHEFS | NHANES Follow-up Study Cohort |
| NHLBI | National Heart Lung Blood Institute |
| NIH | National Institutes of Health |
| non-CVD | Non Cardiovascular Disease |
| PAL | Practical Approach to Lung Health |
| PALSA | Practical Approach to Lung Health in South Africa |
| PALSA PLUS | Practical Approach to Lung Health and HIV/AIDS in South Africa |
| PHC | Primary Health Care |
| PHDS | Program in Health Decision Science |
| PI | Principal Investigator |
| Prof | Professor |
| PTCA | Percutaneous Transluminal Coronary Angioplasty |
| QALY(s) | Quality Adjusted Life Year |
| RCT | Randomised Controlled Trial |
| Ref | Reference |
| ROC | Receiver Operator Characteristic |
| SA | South Africa |
| SADHS | South Africa Demographic and Health Survey |
| SAMA | South Africa Medical Association |
| SANAS | South African Accreditation System |
| SASH | South African Stress and Health Survey |
| SOPH | School of Public Health |
| SRQ 20 | Self Reporting Questionnaire |
| STI(s) | Sexually Transmitted Infection(s) |
| STRETCH | Streamlining Tasks and Roles to Expand Treatment and Care for HIV |
| SU | University of Stellenbosch |
| TC | Total Cholesterol |
| TDA | Tanzania Diabetes Association |
| UCT | University of Cape Town |
| USA | United States of America |
| UWC | University of the Western Cape |
| WCDOH | Western Cape Department of Health |
| WHO | World Health Organisation |

## Background

**Burden of Disease**

**Sub-Saharan Africa**

Over the last few decades, the public health challenges have increased in Sub-Saharan African countries as the epidemiological transition has progressed. In addition to the ongoing burden of pre-transitional diseases related to poverty, the epidemic of HIV/AIDS has escalated to become a major cause of death, particularly in younger adults, while injuries and violence remain endemic. Chronic diseases (CD) especially cardiovascular disease, diabetes, chronic respiratory disease and certain cancers, have also steadily emerged, along with mental health conditions, as major threats to health across the spectrum of Sub-Saharan Africa’s diverse racial, ethnic and social class groupings.

**South Africa**

In the 2004 update of the Global Burden of Diseases it was estimated that chronic non-communicable diseases account for 28% of the total disability adjusted life years. Chronic conditions such as cardiovascular diseases, diabetes mellitus, respiratory diseases and cancer accounted for 12% of the total disease burden, while common neuropsychiatric disorders accounted for 6% of the total burden (WHO 2006, Mayosi 2009). In the short term, despite the high death rates caused by AIDS in South Africa, actuarial projections suggest that deaths from all CD will also increase from 565 deaths per day in 2000 to 666 deaths per day in 2010. (Bradshaw, 2006). In a recent publication entitled ‘ A race against time’ Leeder *et al* has reported that in 2000, CVD mortality rates in South Africans of working age (35-64 years) were already higher than in people of similar age in the USA and Portugal (Leeder, 2004). They further predicted that the CVD mortality in South Africa for this age group would increase by 41% between 2000 and 2030. Given that nearly half of the deaths caused by CVD occur before the age of 65 years, four times the rate of the USA, these premature deaths will clearly have a major negative economic impact on the country. The distribution of chronic non-communicable disease mortality is affected by socio-economic class and clearly illustrated in data from Cape Town where the poor communities of Khayelitsha had higher CD mortality rates than those of wealthier communities in the northern and southern subdistricts (Groenewald 2008).

The African component of the INTERHEART study confirmed that although the risk factors responsible for myocardial infarction in South Africa were similar to those in other countries, the population attributable fractions differed from countries outside of Africa. In general the risk factors had a higher impact in South Africans than in other regions of the world (Steyn, 2005). CD risk factors are common in all ethnic groups in South Africa. An estimated 7 million smoke, 6.3 million have hypertension, 1.5 million have type 2 diabetes and 5 million have raised blood cholesterol (Levitt, 1993, Levitt 1999, Steyn, 2006i). In addition to high smoking rates South Africans also have high rates of physical inactivity and consume unhealthy diets with a large proportion of the burden of CD attributable to these elements (Norman 2007). Recent surveys suggest that prevalence of diabetes and hypertension are increasing in the relatively recently urbanised sectors of society (unpublished data, D Levitt, UCT).

Data on the burden of chronic respiratory disease in South Africa is relatively sparse. The most reliable overview of respiratory symptoms and risk factors for respiratory diseases in South Africa has been provided by two Demographic Household Surveys commissioned by the National Department of Health, the first in 1998 (Ehrlich 2005). Symptoms of chronic bronchitis were lower than that reported from many countries in Europe, but as in other developing countries, the association with cigarette smoking was less pronounced, with exposures to dust and fumes in industry (Hnizdo, 1992), indoor pollution from biomass fuelled fires (Grobbelaar & Bateman, 1991), previous tuberculosis (Churchyard *et al* 2001) and pneumonia emerging as significant additional risk factors (Ehrlich *et al,* 2004). However, a community-based study of COPD in Cape Town found the prevalence of COPD (Stage 2 and above) to be 19.1% in persons aged 40 years and older, the highest of the 12 centres from different continents sampled. Of equal concern were the high recorded rate among women (16.7%) and the high proportion of severe cases (Buist 2007).

The latest review of the burden of diseases in South Africa ranks neuropsychiatric conditions as the third largest contributor to DALY estimates in the country (Bradshaw 2007). The prevalence of mental disorders was determined for the first time in a national representative sample in 2002 during the South African Stress and Health Survey (SASH) (Williams 2008). The study reported that 16.5% of South African adults suffered from a common mental disorder in the year prior to the study with 8.1% of participants found to have anxiety disorder and 4.9% a mood disorder. In the SASH sample, psychological distress was more common among those with low socio-economic status and low social capital, after adjusting for demographic characteristics and life events (Myer 2008). Mental disorders can predispose patients to many common CD, which in turn can contribute to the development of additional mental disorders (Prince 2007). For example, mental disorders are associated with CVD risk factors: smoking is consistently associated with depression and anxiety disorders; and hypertension has been independently predicted by both depression and anxiety in 7-16 year follow-up studies. Depression is also an independent risk factor for stroke, and depression has been shown to independently increase the risk for Type 2 diabetes (Prince 2007).

**Treatment Status of Patients with Chronic Diseases**

Multiple studies have shown that the common risk factors and the associated elements of an unhealthy lifestyle are often undiagnosed and inadequately treated, resulting in high levels of uncontrolled hypertension, diabetes and hyperlipidaemia (Steyn 2008, Steyn 2006ii). In a recent study, levels of hypertension control were worse in patients who attended the public sector community health centres than in those who received care in the private sector (Steyn 2008i). Notably, the poor levels of hypertension control have also been associated with high levels of target organ damage (Peer 2008). Steyn and Levitt studied the quality of care provided for patients with hypertension and diabetes who attended primary care services in the public sector at 18 community health centres in Cape Town. Their findings confirmed those of community-based surveys. Of the 923 patients with hypertension, only 33% had controlled BP (BP≤140/90 mmHg) while 42% of the 455 with diabetes had non-fasting glucose levels below 11.1 mmol/l. In addition, patients’ knowledge about their condition was poor and prescriptions for drugs could not be found in the medical records of 22.6% of patients with diabetes and 11.4% of patients with hypertension (Steyn, 2008ii).

Similarly it was found that less than one half of cases with chronic respiratory diseases had been diagnosed and fewer than one third were receiving treatment (Jithoo 2007). Consequently exacerbations of COPD are very commonly encountered and morbidity is high. In a Burden of Asthma publication published in 2003 by the Global Initiative for Asthma (GINA), South Africa ranked 4^th^ of almost one hundred countries for asthma case fatality rates (Masoli and Beasley 2004), and although in a study of asthma deaths in Cape Town for the period 1995-2000, this rate appeared to be declining (Zar 2001), the majority of deaths were shown to be in poorer communities and remained unacceptably high (Poyser 2002).

The high prevalence rate of common mental conditions found in the SASH study provided the realisation that these mental conditions are seldom diagnosed and also inadequately treated. The SASH study found that only 28% of those with severe and moderately severe mental disorders received treatment. Treatment was mostly provided by the general medical sector, with few receiving treatment from mental health service providers (Williams 2008).

**3.2 Current Health Care in South Africa**

The majority of the population of South Africa is poor and dependent upon the public sector heath services for their medical needs. The large and well developed private health care sector provides services to less than 20% of the population and accounts for almost 60% of total health expenditure. Since the advent of democracy in 1994, South Africa has embarked on a systematic process of health sector reform, moving away from a hospital centred curative based health system to the Primary Health Care (PHC) approach. The District Health System (DHS) was subsequently formally established in the National Health Act of 2003 (Health Act).

An assessment of national policies and resources in South Africa for the prevention and treatment of CD suggests that although these important building blocks for care are in place there is considerable gap between policy and implementation. At a policy level both the national and provincial departments have heeded the message contained in the WHO Global Health Report of 2005 that makes the case for “increased and urgent action to prevent and control chronic diseases”, and have responded by identifying chronic diseases as an additional priority within the already overburdened primary care portfolio. National guidelines for all priority CD have been developed and recommended for implementation in provinces, and a policy of integrated care for CD within clinics has been proposed, with both the Integrated Management of Adult Illnesses (IMAI) and PALSA PLUS (Practical Approach to Lung Health and HIV/AIDS in South Africa) receiving support in different provinces. Finally, and most significant have been the provision of essential drugs for the management of chronic diseases as national policy.

However practical realities militate against effective implementation of these policies. The crushing burden of the triple “epidemic” of tuberculosis, HIV/AIDS and trauma, and human resource constraints in the national and provincial health departments fuelled by ineffective leadership have resulted in demoralisation, resignations and worsening staff shortages, particularly in primary care clinics. The denial of AIDS by top national leadership; the long delay in addressing the HIV/AIDS epidemic effectively; widespread stigma regarding mental illness; poor working conditions; and insufficient training of nurses are just some of the reasons that must be cited as contributory causes of this situation. Consequently primary care facilities, more abundant in South Africa than in 1994, are generally inadequately staffed, overburdened and suffer shortages in the regular supply of medications, and in some instances, of equipment. Priority is therefore given to managing tuberculosis, HIV/AIDS, trauma and acute diseases, and at best, acute presentations of chronic diseases, such as psychotic episodes in schizophrenia (Steyn 2008i, Petersen 2009).

There is an urgent need to reform primary care provision for CD at clinic level by equipping and empowering frontline clinicians (both nurses and doctors) with the necessary communication, knowledge and practical skills and tools in the diagnosis and management of CD.

**3.3 Planning in Health Services for Chronic Disease Care.**

The convergence of several developments in South Africa, and in particular the Western Cape, has created the opportunity for building an integrated model of healthcare delivery for CD involving collaboration between community, health services and local institutions (Figure 1). First, the National Health Act requires that communities become involved in the programme of care offered by the local health service. Second, the Western Cape Provincial Government has responded to this requirement through a unique commitment to an enhanced community health worker programme in chronic diseases. The Western Cape DoH has clearly articulated this approach in its strategic plan called the Healthcare 2010 Plan ([www.capegateway.gov.za](http://www.capegateway.gov.za)). Third, members of UCT and other regional institutions, who have developed and are implementing an integrated care model for nurses on chronic respiratory and infectious diseases, have been asked to expand this model to include chronic diseases. The commitment of the Western Cape Government to put in place integrated care guidelines for chronic disease management in 2011 gives us a unique opportunity to develop a research plan evaluating the impact of such a decision that will have widespread implications in the rest of South Africa and potentially other partner countries in the region such as Tanzania.

**3.4 Integrated Primary Care Model for Chronic Diseases proposed by the Chronic Disease Initiative in Africa (CDIA)**

The CDIA is a recently launched chronic disease initiative of Universities in the Western Cape, the MRC, Tanzanian and US collaborators. The CDIA proposes an integrated model for primary care for CD based on the World Health Organisation recommendations outlined in the publication ‘Innovative Care for Chronic Conditions (ICCC) (WHO 2002) (Figure 1). The model aims to strengthen and broaden the health care team by providing tools that improve the effectiveness of care and by mobilising community support and patient involvement in their own care. This protocol addresses three aspects of the integrated primary care model.

The first aspect is to calibrate and validate a feasible cost-effective tool for identifying patients at high risk for cardiovascular disease (CVD) without laboratory testing. The second aspect will be a pragmatic trial testing the effectiveness of our integrated care guideline training program for health care providers in the primary care setting. Finally, a cardiovascular disease prevention policy model will be developed that can be used to evaluate the cost-effectiveness of the integrated care guideline programme through changes in process measures and/or risk factors. Further, the model will be available to forecast future CVD burdens and evaluate the cost-effectiveness of future interventions that prevent CVD or reduce its risk factors.

**Figure 1: Integrated Primary Care Model for Chronic Diseases**

Patients

Clinic-based Staff

Community

CHW

CHW

Managers

Screening in the community with non-lab tool

Training to lead therapeutic groups and support individual patients

Guideline-based training in diagnosis, drug management, investigation, referral, initial advice, monitoring. Training in motivational interviewing and using appreciative inquiry

*Chronic disease detection*

*Chronic disease management*

Audit and feedback of aggregate data.

## Development and validation of a total cardiovascular risk assessment tool for developing countries

**Research question or hypothesis**

Calibrate and validate a non-laboratory based screening tool for cardiovascular disease (CVD) risk prediction in South Africa.

**Background and Significance**

The overall goal of this program of work is to develop a cost-effective screening strategy for those at high risk for cardiovascular disease (CVD) in low-income countries. Given the large global burden of CVD and very limited resources in developing countries, finding low-cost prevention strategies is a top priority. Screening to identify those at higher risk in order to target specific behavioural or drug interventions is a well-established primary prevention strategy.

During the 1970s numerous efforts were made to create models for predicting CVD risk using the associated risk factors by multivariate regression techniques. The most significant of these efforts was an equation derived from the Framingham cohort of both men and women aged 35 to 70 in the US. (Anderson, 1991) This logistic regression model used cholesterol, blood pressure, smoking history, an electrocardiogram and diabetic status to predict the 10-year absolute risk of a first time CVD event —fatal and nonfatal stroke, cardiac arrest, angina, and fatal and nonfatal myocardial infarction (MI). The current version eliminates the ECG. The WHO recently released guidelines for the prevention of CVD (WHO, 2007) that includes two risk charts for CVD. The first set of WHO charts use the same risk factors used in the Framingham risk equation but without HDL cholesterol. The second set of charts removes cholesterol and retains the remaining risk factors. However, the charts have not been validated in any of the WHO regions or countries—developed or developing. Further, given that the WHO charts were not based on a cohort with CVD outcomes, the investigators were not able to evaluate the predictive discrimination or calibration of either of their charts or make any comparisons between them.

Preliminary work in developed country cohorts has tested the hypothesis whether a non lab-based screening tool for cardiovascular disease could predict risk for CVD nearly as well as one that did require blood testing. We have found that in well characterized cohorts in the United States a simple screening tool that does not require lab testing can predict future CVD events as well as one that requires lab testing (Gaziano, 2008). This is important because a lack of financial, human and laboratory resources make lab testing impractical and costly in low resource settings. We compared two Cox models. The first is the “lab-based” model which requires blood testing and the second, which we designate as the “non lab-based” model, requires only history and physical exam measures. In both we compared how well either model could predict first time fatal and non-fatal CVD events in the NHANES I Epidemiologic Follow up Study (NHEFS) cohort. Both predictive models performed well in discriminating risk. In the women, the “lab-based” model performed well in predicting events with a C-statistic (95% confidence intervals) of 0.829 (0.813-0.845) The C-statistic of the “non lab-based” model was 0.831 (0.816-0.847). The test for significant difference between the two c-statistics is a chi-square result of 2.476 (p=0.116). Similar results were found for men. (Figure 2)

The next stage is to show that a screening mechanism using the same risk factors that does not require lab testing can also predict well in a different population. We also wish to test what is the most cost-effective and practical strategy for screening for CVD in South Africa. Therefore, the purpose of this proposal is continuing with the development of a simple risk prediction tool for CVD and to assess its cost-effectiveness in South Africa. Hopefully, the strategies can then be incorporated into national or international guidelines for the management of cardiovascular disease.

**Figure 2. ROC curves for lab and non lab-based CVD prediction tools.**

Women Men

**Design and Outcomes**

**Proposed Model Calibration Approach**

In this protocol, we seek to use various calibration techniques to adjust cardiovascular model inputs to result in model-predicted outcomes that fit the observed mortality data in South Africa. The calibration process will begin by evaluating the disease model populated with the transition probabilities previously used in South African modelling analyses, which were based on the Framingham risk equations (Gaziano *et al*, 2005) and compared to non-lab based measure. These inputs will be used to assess the initial fit of the model-predicted outcomes. During the calibration process, these model inputs will be adjusted so that the model outputs better reflect the observed endpoints from the regional/national database.

We will use several techniques to calibrate the parameters of interest and comment on the accuracy and efficiency of each approach. The technique of interest will be the Nelder-Mead/Downhill Simplex algorithm, which has been broadly used in engineering and physical science optimization problems, but less so in disease and cost-effectiveness models (Byatt *et al*, 2003). The Nelder-Mead algorithm attempts to minimize a non-linear function and is based on the simplex algorithm, which is compatible for calibrating complex disease models to observable clinical endpoints (Nelder and Mead, 1965). An advantage of this approach is that the Nelder-Mead algorithm has previously been found to be a relatively efficient method to calibrate a cervical cancer disease model compared to other calibration techniques (Taylor et al., 2007). However, to assess the robustness of the model results with respect to calibration methodology, we will employ two alternate calibration approaches and compare the results of each technique. The first of these options is to intuitively, or manually, adjust individual inputs without any formal algorithm. This approach is conceptually straightforward, but is subject to the biases and limitations of the analyst who conducts the manual calibration. The second alternative approach is to randomly vary the inputs of interest and evaluate the model results produced by each parameter set to the observed clinical endpoints. Since this approach relies on random sampling from probability distributions, it is less subjective than the manual approach. However, the random search algorithm is not efficient and can result in the evaluation of millions of parameter sets for complex models (Kim et al., 2007, Taylor et al., 2007).

To calibrate the risk score previously developed in the NHEFS cohort, we propose to use the South African Demographic Health Survey of 1998 (or Western Cape data) and the CVD morbidity and death rates from Central Statistics South Africa from 2003 and 2008 to recalibrate our lab-based screening tool for five and ten year predictions. We included the following risk factors in our analysis to compare a lab-based tool to a non-lab based tool, using the value closest to the entry date to the cohort for each patient: age (in single years); gender (men v women); smoking status (current smoker, non-smoker—including former smoker); systolic blood pressure (continuous); ratio of low density lipoprotein cholesterol to high density lipoprotein levels (continuous); body mass index (continuous); current prescription of at least one antihypertensive (yes or no), and diabetes status (yes or no). All data for this analysis is based on de-identified data sets. We will also use the DHS 98 and the relevant census data for validation of the WHO risk charts, using information on age, gender, smoking, blood pressure, and reported diabetes status.

The 1998 South Africa Demographic and Health Survey (SADHS) is the first survey of its kind to be carried out in South Africa since the 1994 democratic national elections. This national cross-sectional study collected information on adult health conditions; sexual, reproductive and women’s health; maternal and child health; adult, maternal, child and infant mortality; fertility and contraceptive use. Preparations for the study started in 1995 and the fieldwork was carried out between late January and September 1998.

They were also asked about recent contact with the health-care system, insurance status, family medical history, personal medical history, other medication use, smoking, occupational health, and lifestyle/habits. Information regarding other demographic factors such as age, education level and population group was also recorded.

The fieldworkers assessed anthropometric measurements, blood pressure, pulse rate, and peak expiratory flow rate on each participant at their home. Systolic and diastolic blood pressure and pulse measurements were taken 3 times with the patient seated and the left arm at the level of the heart after the participant was seated for 5 minutes. An Omron M1 electronic blood pressure manometer was used. In addition information on smoking status, diabetes status, medications for hypertension, and body-mass-index were collected and will be used in the calibration model. Population based means for these values with standard errors will be used to model death rates from CVD.

To assess calibration (the degree of similarity between observed and predicted risks) we will calculate the mean predicted risk of cardiovascular disease at 10 years and the observed risk at 5 years obtained using the 5 year Kaplan-Meier estimate. We then compared the ratio of the predicted to the observed cardiovascular disease risk for patients in the validation cohort in each tenth of predicted risk.

**Risk Prediction Validation**

The calibration of the CVD risk score is important for national projections of disease incident and projections of future mortality based on changes in the various risk factors. However, for its reliable use in predicting individual risk, validation in prospective cohorts is ideal. CVD prediction charts for the different regions of the world that do not require lab testing have been suggested (Mendis *et al*, 2007; Lim *et al*, 2007) by the WHO but they have never been compared with any of the standard risk prediction rules or validated in any cohort. We will attempt to validate both the non-lab based prediction tool from our work in the NHEFS cohort as well as the WHO risk prediction charts.

To develop and validate a new score we will use the cohort of approximately 2000 patients described in the main programmatic trial outline below in a separate protocol who are over the age of 35 and without prior history of CVD. Our endpoint for censoring as described will be all cause mortality and CVD mortality encompassing the appropriated ICD codes for ischemic heart disease, stroke and hypertensive heart disease. Participants will also be asked to provide their South African identity number. This will be used to link with the national mortality register at 5 and 10 years from the start of the study. Unfortunately, data collection methods and resources do not allow for us to confirm non-fatal CVD events. We hope to eventually use this pilot data for use in a larger cohort to evaluate both morbidity and mortality data for a more refined risk scoring mechanism.

Therefore we propose to use the information from the cohort of patients enrolled in the cluster randomized control trial (described below) to validate the risk score. The initial evaluation will allow us to develop a risk score for total mortality. In the NHEFS cohort above, a risk prediction tool using non-lab based values had remarkable discrimination in predicting those at high risk for overall mortality with a C-statistic of 0.85 and 0.86 for women and men respectively. We will then test for predictive accuracy in predicting CVD mortality even though we recognize that there is likely to be some misclassification due to the incomplete death certification in South Africa.

We included the following risk factors in our analysis to compare a lab-based tool to a non-lab based tool, using the value closest to the entry date to the cohort for each patient: age (in single years); gender (men v women); smoking status (current smoker, non-smoker—including former smoker); systolic blood pressure (continuous); ratio of low density lipoprotein cholesterol to high density lipoprotein levels (continuous); body mass index (continuous); current prescription of at least one antihypertensive (yes or no), and diabetes status (yes or no). Having calibrated the model from the national data we can then apply this data from this specific validation cohort. We calculated the 5 year estimated cardiovascular disease risk for each patient in the validation dataset. For development and validation of the new risk score we will use Cox proportional hazards regression to assess the “lab-based” model and compare it with the “non lab-based” model.

## Integrated Care Guideline Trial

### Full title

Effectiveness of an integrated care guideline training programme on the processes and outcomes of non-communicable chronic diseases in primary care in South Africa: a pragmatic cluster randomised controlled trial.

### Purpose of the study

To evaluate the effectiveness of an integrated guideline-based training programme for primary healthcare nurses and doctors on processes and outcomes of non-communicable chronic diseases, compared with current training and support for chronic diseases.

The corresponding hypothesis is that equipping nurse middle managers as outreach trainers to train primary care staff in integrated adult case management based on a simplified short (101 page), syndromic guideline, can simultaneously improve the quality of care for chronic diseases of lifestyle, chronic respiratory disease and mental health, in resource-constrained non-physician led primary care services.

### Background

The intervention being evaluated in this study is based on PALSA PLUS, or the Practical Approach to Lung Health and HIV/AIDS in South Africa. PALSA PLUS uniquely combines educational outreach, or brief onsite interactive education, with symptom-based or syndromic guidelines (English 2008). Evidence of effectiveness for both components exists (O’Brien 2007, Grimshaw 2003, Hayes 1995, Wawer 1999, Armstrong Schellenberg 2004, El Arifeen 2004). Syndromic approaches have been popularised as tools for health workers in LMICs through the WHO’s programmes for STIs (WHO 1991) and childhood illness (Gove 1997), but have been implemented largely using offsite intensive training approaches with minimal follow-up in the field. This model disadvantages programme rollouts in that it is disruptive to clinical services and limits sustainability and coverage.

Originally drawn from the WHO’s Practical Approach to Lung Health (WHO 2005), PALSA PLUS had been expanded over the past 10 years to include treatment of opportunistic infections in HIV, antiretroviral treatment (initially by doctors and later by non-physicians), STIs and antenatal care. By equipping nurse middle managers as outreach trainers, the programme has become embedded in the healthcare system, and has been able to be implemented at scale. Since mid-2006 over 10,000 healthcare workers in more than a thousand facilities across South African provinces have been reached, and implementation has now started in Malawi.

At each stage of its development, the programme has been subject to rigorous evaluation with pragmatic randomised controlled trials (Fairall 2005, Fairall 2008, Zwarenstein 2010, Schull 2009). Pragmatic trials evaluate the effects of health service interventions under the human, financial and logistic constraints of typical, real world situations (Zwarenstein 2008b, Tunis 2004, Schwartz 1967). The current proposal extends the approach and will evaluate whether the intervention, previously shown to be effective for respiratory diseases including tuberculosis and HIV/AIDS (Fairall 2005, Zwarenstein 2010), will remain effective when expanded to include the primary care management of other major categories of chronic diseases. Specifically, we wish to evaluate whether its effectiveness will be diluted by this broader objective. If successful, this project will provide scientific support for the validity of transversal programmes of care for chronic diseases in primary care in resource-limited settings, with a particular focus in its application to cardiovascular and pulmonary diseases, and its ability to strengthen primary care services.

### Study design

The study is a pragmatic cluster randomised controlled trial with clinics randomised to two parallel arms, and outcomes assessed on individual patients. Clinics will be randomised within health sub-district strata. Stratification is necessary to control for differences in the ratio of infectious to chronic diseases within communities within the Western Cape. Randomisation will be carried out by the trial statistician before the intervention is implemented or patients recruited.

### Characteristics of the study population

Clinics

Public-sector primary care clinics in the Eden and Overberg districts of the Western Cape province which provide care to underserved communities are characterised by high burdens of both infectious and chronic diseases. The 38 largest fixed facilities will be included in the trial (Appendix 1). These clinics service around or more than 10 000 attendances per year, and are staffed by nurse practitioners, doctors and community health workers, all of whom participate in care delivery for chronic diseases. The Eden and Overberg districts have been selected ahead of others, because doctors are in short supply, and services are provided mainly by nurses. This will allow the trial’s results to be generalisable to similar LMIC settings where non-physicians provide care.

Patients

Inclusion criteria:

- Age ≥ 18 years; and
- Planning to reside in the area for the next year; and
- Written consent to participate in the study

Four cohorts are defined. Patients may fulfil inclusion criteria for more than one cohort. Inclusion criteria based on target chronic disease:

| **Target condition** | **Inclusion criteria** |
| --- | --- |
| Hypertension | Self-reported hypertension requiring medication |
| Diabetes | Self-reported diabetes requiring medication |
| Chronic respiratory disease | Self-reported asthma/ chronic bronchitis/ emphysema requiring medication OR  Cough and/or difficult breathing >2 weeks (and not on treatment for tuberculosis in the past 3 months) |
| Depression | The Center for Epidemiologic Studies Depression Scale (CES-D 10 Scale) score of 10 or more (Andresen 1994) |

Exclusion criteria:

- Inability to meet the above criteria
- Acute and/or terminal condition precluding participation such as AIDS or cancer.
- Psychiatric diagnoses precluding participation such as schizophrenia, dementia and other cognitive impairment measured by self report or medical history.
- For chronic respiratory disease: patients who have received tuberculosis treatment in the preceding 3 months will be excluded.

### Endpoints and sample size calculations

The primary endpoint selected for diabetes, hypertension and chronic respiratory disease is ***treatment intensification***, based on research that identifies clinician inertia as a key reason for failure to control these non-communicable chronic diseases (Van Bruggen 2009, Ho 2008, Rodondi 2006, Turchin 2008, Wang 2007). Definition of treatment intensification is dependent on the target condition (Table 1). For depression, ***case detection*** was selected as the primary outcome because the condition is grossly under diagnosed in primary care – as many as 1 in 5 people attending primary care services are suffering from a mental health condition, mostly depression, yet only 5% of those with the condition have been diagnosed.

Background rates and expected effect sizes for diabetes and hypertension were obtained from prior local and international research studies (Charlton 2008, Nau 2004, Grant 2007). Intracluster correlation co-efficients were drawn from published estimates from a number of cluster randomised controlled trials in each disease area (O’Connor, Fairall 2005, Morrell 2009, Rahman 2008). All sample size calculations are for two-sided tests and are powered at 85%. The sample sizes have been inflated by 20% to allow for losses to follow-up at the follow-up interview planned for 12-15 months after recruitment.

We anticipate substantial co-morbidity. Patients with more than one target condition will be eligible for more than one cohort. Based on reported levels on co-morbidity, we estimate that we will need to recruit 121 patients per clinic to realise the sample size for all cohorts (4598 patients in total).

Planned secondary endpoints include disaggregation of primary endpoints, measurement of other processes of care including screening for complications (e.g. dilated eye exams), monitoring control (e.g. annual HbA1C), symptoms (St Georges Respiratory Questionnaire), changes in risk factors for chronic diseases (e.g. systolic blood pressure, waist circumference), productivity, healthcare utilisation, cardiovascular events (e.g. stroke), mortality, newly commenced on simvastatin, and new diagnosis of hypertension , diabetes, chronic respiratory disease, TB and depression.

Due to the considerable burden imposed by venesection, sample size estimates have also been completed for one of the secondary outcomes, HbA1C in diabetic participants. In order to show a difference of 0.5% (8.8% in the control group vs. 8.3% in the intervention group) we need to complete venesection on 30 diabetic patients in 10 clinics in each group (i.e. 600 diabetics from 20 clinics in total). This contains the logistical burden and cost associated with including HbA1C as a secondary outcome. In these calculations, we assumed a standard deviation in HbA1C of 1.2% from the ACCORD trial (Williamson 2007) and an intracluster correlation co-efficient of 0.05.

**Table 1: Primary outcomes and revised sample sizes**

| **Target condition** | **Primary outcome** | **Control** | **Int** | **ICC^1^** | **Required cluster size** | **Cumulative required per clinic** |
| --- | --- | --- | --- | --- | --- | --- |
| Diabetes | Treatment intensification:  Increase in dose of oral hypoglycaemic/ insulin *or*  Addition of new oral hypoglycaemic / insulin *or*  Addition/ increase in dose of ACE inhibitor *or*  Addition of aspirin *or*  Addition/ increase in dose of statin | 0.25 | 0.36 | 0.04 | 60 | 60 |
| Hypertension | Treatment intensification:  Increase in dose of antihypertensive medication *or*  Addition of new antihypertensive *or*  Addition of aspirin *or*  Addition/ increase in dose of statin | 0.25 | 0.36 | 0.04 | 60 | 90^2^ |
| Chronic respiratory disease | Treatment intensification:  Addition of beta-agonist *or*  Addition of ipratropium bromide *or*  Addition of oral theophylline *or*  Addition/ increase in dose of inhaled corticosteroid | 0.15 | 0.25 | 0.02 | 27 | 90^3^ |
| Depression | Case detection:  Started on antidepressant medication *or*  Referred for counselling *or*  Referred to psychiatric services | 0.04 | 0.10 | 0.04 | 60 | 105^4^ |

^1^ ICC: intracluster correlation coefficient

^2^ Assumes 50% of those with diabetes also have hypertension (Steyn 2008 i)

^3^ Assumes 33% of those with either diabetes or hypertension will have a chronic respiratory disease (Ho 2008)

^4^ Assumes 50% of those with diabetes and/or hypertension and/or chronic respiratory disease will score 10 or more on the CESD-10 (Prince 2007).

### Interventions in intervention and control groups

Usual training and support for chronic diseases will continue in both groups. PALSA PLUS training has been implemented throughout the Eden and Overberg districts with over 70% of staff trained by March 2009. This training focuses on infectious disease priorities, but includes chronic respiratory disease because this is required for the integrated case management of tuberculosis suspects. Maintenance of PALSA PLUS training will continue throughout the trial in the control group. This involves infrequent (2 per year) follow-up visits to clinics already trained, distribution and engagement with an annual update of the guideline, and support for nurse trainers including quarterly meetings and monthly newsletters.

A new group of nurse managers will be equipped as outreach trainers for the trial. These managers will assume responsibility for ongoing PALSA PLUS maintenance at intervention facilities, and for the training of staff in the expanded chronic disease content.

### Patient recruitment and data collection

This is shared with the validation project and economic evaluation and described in detail under: Recruitment and enrolment (page 25), Research Procedures and Data Collection (Page 26). Patients will be enrolled just before training is due to start in intervention clinics in a sub-district. Patients will be re-interviewed once, 12-15 months after training started in intervention facilities in a sub-district. This is necessary to allow adequate opportunity for health workers to intervene in their care, given that chronic disease patients undergo clinical review 3-6 monthly.

**Figure 3: Trial design**

*Randomization*

*Recruitment*

*Compare*

19

clinics

2299

patients

(121

per clinic)

**Intervention**

Usual care

•

Integrated care

guideline

•

Outreach training

programme

**Control**

•

Usual care

19

clinics

2299

patients

(121

per clinic)

Follow

-

up:

Clinics

Patients

Follow

-

up:

Clinics

Patients

38 Eden and Overberg clinics

*(stratified by*

*health sub*

*-*

*district)*

INTERVENTION

*Training starts*

PATIENT

INTERVIEWS

Baseline (pre

-

intervention)

Follow

-

up

12

-

16

months post recruitment

Initial:

8

-

12 sessions

over 4 months

Maintenance:

Quarterly

Ongoing

###

### Data analysis

Outcomes will be analysed on an intention-to-treat basis. For the primary outcome, a logistic regression model will be used to evaluate the effect of the intervention with stratification as a co-factor. The model parameters will be estimated by the generalised estimating equations (GEE) approach which takes into account the clustering of patients within clinics. Odds ratios will be presented with 95% confidence intervals.

Secondary analyses include: (a) analysis of the primary outcome but restricted to the subgroup of patients in whom control at baseline has been determined to be inadequate (e.g. treatment intensification among hypertensives with BP >140/90 at enrolment, treatment intensification among diabetics with HbA1C >7% at enrolment, treatment intensification among patients with chronic respiratory disease and frequent and/or severe symptoms as measured by the St George’s Respiratory Questionnaire (symptoms and activity components), treatment intensification among patients not receiving treatment for depression at enrolment); (b) extent to which the intervention effect is modified by co-morbidities by comparing intervention effectiveness across co-morbidity subgroups (1, 2, 3 or 4 or more chronic diagnoses) and the use of joint modeling techniques; (c) extent to which the intervention effect is modified by control and adherence, measured using the Revised 10-item Hill-Bone Blood Pressure Compliance Scale for South Africa. This last analysis will be restricted to patients with hypertension.

## Economic Evaluation Model

The purpose of this project is to develop a CVD model which can be used to estimate the health and economic consequences of cardiovascular prevention interventions in the South African population and in high-risk subpopulations. The model will incorporate risk factors and natural history data on ischemic heart disease (angina, myocardial infarction, and sudden cardiac death) and stroke, and will draw upon primary data from the 1998 Demographic Health Study, Statistics South Africa as well as data from the published literature and publicly available databases. With this model, it will be possible to perform cost-effectiveness analyses of CVD management and prevention interventions which incorporate long-term health and economic consequences of CVD prevention, including gains in life expectancy and savings of health care costs.

Such a model will also permit policy makers at the national level to forecast the national impacts of preventive interventions on future CVD incidence rates, CVD mortality, total mortality, and total medical care costs. Such forecasts can help to set priorities for research and development of preventive strategies, by identifying those areas where effective prevention would deliver the most “bang for the buck”.

### Background and Significance

Limited resources for both health care and prevention force choices to be made under strict budgetary limits. Not every promising CVD prevention program can be implemented to the desired extent. Public health departments operating with fixed budgets are forced to set priorities among preventive services, and among primary prevention, early detection, and clinical treatment. Furthermore, it is unrealistic to expect most prevention or intervention studies to follow subjects long enough or in sufficient numbers to reveal the lifetime costs and consequences in terms of survival, quality of life, and lifetime medical costs, let alone disease incidence. As a result, simulation models are needed in order to project the implications, at both the individual and societal levels, of changes in risk factors. Such computer models would build upon available data on the associations between CVD risk factors and CVD incidence, the prevalence of risk factors in the population, the natural and treated history of disease, and the effects of CVD on survival, quality of life, and medical care cost.

The purpose of the cost effectiveness analysis (CEA) in health is to compare the relative value of different options in improving the quality or length of life. The result of such an analysis is usually presented as a ratio (cost/health outcome). The denominator is some improvement in health such as years of life gained or heart attacks prevented. The numerator reflects the costs to achieve the health gain. No specific consensus on acceptable or appropriate cost per health unit gained has been made. However, the Commission on Macroeconomics and Health (WHO, 2001) has suggested that acceptable cost-effectiveness ratios might be around 2- 3 times the GDP per capita. Nonetheless, even without consensus on a threshold for tolerable costs, CEA allows for choices to be made that maximize a health benefit for a given expenditure.

### Preliminary studies

Recently, the South African Hypertension Society adopted and revised its new guidelines [based on the JNC VI and VII guidelines]. As South Africa is a multi-ethnic country where epidemiologic and cost data were available, we therefore felt this provided a unique and timely opportunity to conduct a cost-effectiveness analysis comparing the two approaches to help inform the potential consequences of the various guideline choices for hypertension. The study compared six strategies for different thresholds for the initiation of drug treatment—two different blood pressure levels (160/95 and 140/90 mm Hg taken from the past and the newly proposed South African guidelines respectively), and four different levels of absolute CVD risk over 10 years (40%, 30%, 20% and 15% calculated using the Framingham risk function). All six of these strategies were also compared to one of no primary prevention treatment.

The results of this analysis which we previously showed as preliminary were published in *Circulation* (Gaziano, 2005). The four “absolute risk” strategies had the four lowest incremental cost-effectiveness ratios. The strategy of initiating antihypertensive therapy for those individuals with a predicted 10-year CVD risk > 40% had an incremental cost-effective (C/E) ratio of $700 per quality-adjusted year of life gained compared to no treatment. The “absolute risk of CVD > 30%”, “absolute risk of CVD > 20%” and “absolute risk of CVD > 15%” had C/E ratios of $1600, $4900, and $11,000 per QALY respectively. On the other hand both the 1995 South African guidelines and the new guidelines were more costly and resulted in fewer gains in quality adjusted life-expectancy than the “absolute risk of CVD > 15%” strategy, and were thus dominated by the less costly absolute risk treatment strategies in the cost-effectiveness analysis.

Table 2 compares the projections of morbidity and mortality from our model with the estimates from the available literature on South Africa. In general, the model was able to accurately project the number of deaths due to ischemic heart disease and stroke, the incidence of stroke and the overall direct cost of CVD.

| **Table 2. CVD Model Predicted and Actual Outcomes in South Africa** | | |
| --- | --- | --- |
| Outcome | Model Prediction | Published Reports (range) |
| Annual Death due to IHD | 12% | 5-13% |
| Annual Death due to Stroke | 14% | 8-16% |
| Stroke rate (per 100 000) | 323 | 100-500 |
| Annual Direct Cost of CVD ($US millions) | 690 | 620-810 |

### Overview

The end product of this research will be a set of specifications for a CVD Prevention Policy Model, incorporating CVD risk factors, ischemic heart disease, and stroke. The model design will be population-based for the South African population, which means that it will be able to project numbers of cases prevented, detected, and treated, as well as national CVD mortality, morbidity, and cost, attributable to interventions. It will also be designed to facilitate cost-effectiveness analyses of preventive interventions for which data on risk-factor modification and program cost have been obtained.

**Base case**

Since the goal of our study is to evaluate primary prevention strategies, the simulation will begin with a population over the age of 35 and free of CVD. The base case for comparison in the primary cost-effectiveness analysis will be conducted from a societal perspective data and will look at standard care in South Africa for CVD. The US Panel on Cost-effectiveness in Health and Medicine wrote a textbook on cost-effectiveness methodology. The panel’s recommendations are a consensus of experts on how to conduct and report a CEA using consistent methodologies (Gold, 1996). These recommendations were also adopted by the Disease Control Priorities Project in Developing Countries (DCP2) textbook outlining cost-effective strategies for disease control in developing countries (Jamison, 2006). This textbook was co-sponsored by the WHO, the World Bank, and the Fogarty International Center and funded by the Bill and Melinda Gates Foundation. We will adhere to those recommendations in our analyses.

**Study design**

State-transition simulation models, also called Markov models, will be developed to assess the cost-effectiveness of the integrated care guidelines for CVD to compare to the base case. The model will be programmed in Excel. We will begin with the model for South Africa where we already have some experience and data for input. Once this model is completed, we will be able to build a similar model for Tanzania changing the inputs derived from the epidemiological data in Tanzania. Effects will be measured in life years saved, QALYs and DALYs. Incremental cost-effectiveness (C/E) ratios will be calculated for each of the 3 strategies compared to the base case under consideration. All costs and benefits are discounted at 3% per year consistent with current guidelines.

**Model Structure**

**Disease Model**

A decision analytic Markov model comparing the two different strategies of care for the care of patients with risk factors for or advanced CVD: standard care and guideline based case management will be built. A Markov model simulates transitions between distinct health states that would occur over a lifetime in a cohort of patients undergoing a selected intervention strategy. Mortality and morbidity data derived from published literature will be used to derive the model probabilities, and costs from the study and project events will tabulated and the outcome for each strategy will be quantified in terms of costs per quality-adjusted life year (QALY) gained.

**Community Population**

The model will incorporate the baseline demographics and estimates from the clinical sites to determine the mean age and risk factor profile for the study populations at baseline. Methodology to collect this information is described below. Using this information we can than predict the likelihood of future vascular events including stroke, fatal and non fatal MI, and the need for hospitalization. For those with prior CVD we will further subdivide the population into the various groups of patients entering into the study: angina, MI, or stroke. Since these three groups have different probabilities of future events we will model them separately and aggregate their costs and benefits over the whole population in proportion to their makeup in the study population.

### Model Structure

The programme will use a decision analytic Markov model built in Excel to model the course of the study population beyond the time of the trial until their death. The time horizon would be for the remaining life of the cohort assuming that the case management system would remain in place. Different assumptions, however, could be made regarding how long differences between the two intervention arms would persist if the case management intervention were withdrawn or if changes in risk factors would reverse if the intensity of the intervention were diminished. These different assumptions will be addressed in sensitivity analyses.

Each year the study population faces a risk of having a cardiovascular disease event (myocardial infarction, cardiac arrest, angina, or stroke), requiring an intervention (angioplasty or surgery), or dying from a CVD or non-CVD related cause as outlined in the figure below. It is recognized that the invasive interventions are used at a much lower rate than in the developed countries. We will then use the study information regarding improvements in the various risk factors such as reductions in blood pressure, changes in the lipid profile, use of medications such as aspirin, and smoking rates to model relative risk differences in the likelihood of events over time based on changes in these risk factors or treatments.

###### Determination of Costs

Costs will include the costs of the intervention itself derived from the intervention as outlined in greater detail below as well as estimated costs of future hospitalizations and induced costs related to the intervention. Members of the UCT Health Economics Unit will be encouraged to participate in the collection of the input costs for the model. They have been working to cost out various items of care at the Vanguard clinical site along with members of the UCT Department of Medicine on a project for management where Dr. Gaziano is serving as an advisor and we will aim to collaborate and extend some of that work for this project.

Based on similar economic evaluations, the major costs of the intervention (excluding the research components) include the following:

1. Design and Development costs: these include primarily labour costs spent by quality improvement and clinical personnel finalizing the guideline protocols. These costs will have to be amortized over the life of the presumed intervention.
2. Implementation and Education Costs: these include training time for the managers and nurses to learn to use guidelines and risk tools, as well as training of clinical and office personnel who interact with case management personnel. These costs will need to be amortized over the course of the likely retention period of the individual practitioner.
3. Clinical Operating Costs: these include the ongoing costs to conduct the intervention, including time of the manager, as well as overhead and supply costs.
4. Information Technology Support Costs: these include ongoing support services such as data monitoring and backup, network support services, updates, and modifications.
5. Patient Utilization Costs: these include increases in testing, medication use, visits, and referrals that result from the intervention, calculated directly from the results of the intervention (i.e., change in utilization in the intervention arm compared with the change in the usual care arm). These may be offset by efficiencies gained by transferring some patient care activities from physicians to lower cost personnel such as a nurse practitioner.
6. Direct non health care costs including transportation to the clinic, assistance from caretakers and the costs of the patient seeking care will be included if significant

**Determination of Effectiveness**

The model will determine patient benefits by quantifying the decrease in cardiac risk factors (e.g., LDL and blood pressure, and smoking rates) and increase in cardiac medication use (e.g., blood pressure medications, aspirin, ACE-Inhibitors to be consistent with the drug formularies) from the intervention and then estimating the benefits of these changes from the medical literature, including trials of medications and studies of risk factor modification. We will use studies of other multi-factorial interventions and observational modelling studies to determine whether the benefits of carrying out multiple prevention strategies results in an additive, less than additive, or synergistic changes in cardiac risk. Confidence intervals on the relative risks of these benefits will be reported for sensitivity analyses around the key parameters and for a probabilistic sensitivity analysis.

The model will assume that prevented cardiac events will be divided among cardiac death, coronary interventions (PTCA and CABG) and other hospitalizations for CAD in a distribution similar to that in current practice. Changes in quality of life will be taken from the literature on the utilities of different health states following non-fatal cardiac events. The model will then estimate the increase in quality-adjusted life-years (QALYs) generated by the intervention.

**Data Collection**

In each model the adult population will be defined according to age, sex, tobacco status, and BMI in the low cost strategy and age, sex, tobacco status, cholesterol, and diabetes status in the high cost screening strategy. Growing on the experience from evaluating the prevalence, distribution, and medications patterns of hypertension in South Africa, (Steyn *et al*, 2001) we will use the Demographic and Health Survey (DHS) of South Africa to determine the smoking rates, body mass index, and family history of CVD. Estimates on diabetes status and cholesterol levels will be pulled from smaller representative studies (Levitt *et al* 1993, 1999) or our new pilot data since this information was not collected in the DHS. We will use the Statistics South Africa’s database available in the public domain to determine the population by age deciles in South Africa. Utility weights will come from EQ-5D results from the above integrated care guideline as well as other local and international sources.

**Costs**

Resource use costs will include direct and non-direct health care costs, including informal caregiver time, and patient time, consistent with current recommendations for cost-effectiveness analyses. Direct health care costs primarily considered will be screening costs (e.g. clinic visits), costs of the medication, health care worker time, and costs of treating CVD events (both acutely and chronically). Direct non health care costs including transportation to the clinic, assistance from caretakers and the costs of the patient seeking care will be included if significant.

Screening costs will include the cost of the visits and the costs of the screening blood tests. The cost per visit will be according to the “scale of benefits” in South Africa. These are the levels that are reimbursed by third party payers and are considerably less than those recommended charges by the South African Medical Association (SAMA) and medication costs will be derived from local drug formulary information. Side effect costs and additional laboratory testing will be assessed. Treatment costs for the morbid sequelae of the risk factors—angina, myocardial infarction, sudden cardiac death, and cerebrovascular accident—are not directly itemized for each diagnosis in South Africa. Estimates from a bottom up approached used in the DCP2 analyses will be used.

**Sensitivity Analysis**

Univariate sensitivity analyses will be conducted on all input parameters including costs, prevalence estimates, and transition profanities, quality-of-life values, the relative risk reduction from treatment, and the discount rate. A probabilistic multivariate sensitivity analysis will then be performed with a Monte Carlo simulation of 1000 iterations.

## Recruitment and enrollment

Patient recruitment, enrolment and data collection will be conducted jointly for the validation study and trial. Patient inclusion and exclusion criteria differ between the validation study and trial, and have been described under the sections detailing the respective studies. Criteria are summarised in Table 3. We expect a large proportion of patients will qualify for both the validation study and trial. Others will be eligible for one of these studies, and some for neither.

**Table 3: Inclusion/ exclusion criteria for patients participating in validation study and trial**

| **Study** | **Validation study** | **Trial** |
| --- | --- | --- |
| Inclusion criteria | - Age ≥ 35 years *and* - Written consent to participate in the study | - Age ≥ 18 years *and* - Planning to reside in the area for the next year *and* - Written consent to participate in the study *and* - Self-reported diabetes on treatment *or* - Self-reported hypertension on treatment *or* - Self-reported asthma/ chronic bronchitis/ emphysema on treatment *or* - Cough/difficult breathing > 2 weeks (and not on treatment for tuberculosis in the past 3 months) *or* - The Center for Epidemiologic Studies Depression Scale (CES-D 10 Scale) score of 10 or more(Radloff 1977, Andresen 1994, Cheung 2007) |
| Exclusion criteria | - Prior cardiovascular event (e.g. stroke, myocardial infarct) - Acute and/or terminal condition precluding participation such as AIDS or cancer - Psychiatric diagnoses precluding participation such as schizophrenia, dementia and other cognitive impairment measured by self-reported or medical history. - Unavailable or no South African identity number | - Acute and/or terminal condition precluding participation such as AIDS or cancer - Psychiatric diagnoses precluding participation such as schizophrenia, dementia and other cognitive impairment measured by self-reported or medical history. |

Participants will be recruited at the 38 Eden and Overberg public sector community health centres (CHC) and clinics selected for inclusion in the trial. The majority of patients seeking care from these public sector clinics are poor and from the underserved sectors of the South African population. Of this group of possible study participants the majority will either be people of African descent (speaking predominantly IsiXhosa) or people of mixed race ancestry (speaking predominantly Afrikaans). Most people will also have some proficiency in English. Participants will be recruited from patients attending ambulatory outpatient services, and patients requiring emergency care, urgent referral or terminal care will be excluded.

Trained fieldworkers will be responsible for recruitment, enrolment and data collection procedures, under the supervision of the trial co-ordinator, a nursing sister with 20 years’ fieldwork experience in community and clinic-based surveys. In each clinic waiting room patients 18 years or older attending ambulatory services will be invited to be considered for participation in the study, and if willing, will be asked to meet a fieldworker after their consultation with the nurse or doctor and collection of medication from the dispensary. Trained fieldworkers will screen these patients using a structured questionnaire, and eligible patients will be invited to participate further and informed consent will be obtained. Screening and enrolment procedures are summarised in Figure 4.

**Figure 4: Overview of screening procedures and enrolment for validation study and trial**

##

## Research procedures and data collection

Participants who are eligible and provide consent for the validation study will be asked a few questions to establish smoking status, treatment for hypertension and diagnosis of diabetes, and will undergo the following measurements: blood pressure, height, weight, hip and waist circumference. Blood pressure will be taken using an electronic device, and will follow the American Heart Association protocol, where multiple measurements (three) are taken over the period of the interview a minimum of two minutes apart. The average of these readings will be used (Pickering 2005). Participants in the validation study will also undergo venesection and blood will be drawn for triglycerides and HDLC/LDLC ratio. Trial participants with diabetes requiring medication will undergo an HbA1C. Venesection will be performed by the usual nurses responsible for phlebotomy at the clinic, but bloods will be transported to and analysed by a private pathology service. The need for these exploratory assessments and analyses is illustrated by the finding that in community surveys in the target areas, the HDLC to TC ratio was found to be higher than in Caucasian populations (Mollentze 1995, Steyn 1989, Oelofse 1996). This suggests that TC levels will not serve as a satisfactory proxy measure for cardiovascular risk associated with apo B-related lipoprotein particles such as LDLC. We therefore propose to explore whether HDLC/LDLC ratios might serve as a better indicator of lipid-related risk for cardiovascular disease in this population.

Participants eligible for the trial will undergo a structured interview administered by a trained fieldworker. The same questionnaire will be applied irrespective of the target condition reported by the participant, as data collected are applicable across all cohorts.

Data collected will include:

- Patient contact details to facilitate administration of follow-up interviews
- Demographic data (date of birth, sex)
- National identity numbers to permit linkage with the national mortality register, which is estimated to record more than 90% of all deaths in South Africa (Statistics South Africa 2005)
- Hospitalisation folder numbers, if available, to permit linkage with hospitalisation databases
- Measurement of cardiovascular risk parameters (e.g. blood pressure, waist circumference)
- Processes of care received at the clinic: e.g. foot and eye examinations, counselling re weight loss and smoking cessation, referrals
- Medication received at the clinic (name, dose, duration)
- Dates prescriptions were filled at the clinic (to establish proportion covered by filled medication), as a proxy of adherence
- Health related quality of life (EuroQol 5D) to establish local utilities for a range of health states in those with chronic diseases for use in the economic model. The EuroQol has been widely used and validated in South Africa, and approved translations are available for the languages required for this study.
- Symptom severity: St Georges Respiratory Questionnaire (Jones 1991)
- Smoking history
- Health care utilisation (clinic visits, hospitalisations, use of private providers)
- Income and changes due to current illness
- WHO’s Disability Assessment Schedule II (Posl 2007)
- Expenditure on health care utilisation

The questionnaire is currently in development, and will be piloted prior to implementation in the trial. Once approved by the HREC it will be translated into Xhosa and Afrikaans in accordance with internationally accepted practices (Guillemin 1993). Certificates of translation will be submitted to the HREC.

Interviews will be conducted using computers, which we have previously used in similar field work data collection exercises in South Africa (Seebregts 2008). Based on previous experience with a similar questionnaire, interviews administered using such computers will last between 20 minutes and one hour, depending on the number of medications to be documented, and the extent of healthcare utilisation reported by the respondent.

Recruitment will occur during the month prior to the commencement of educational outreach and is estimated to last up to two weeks in each clinic. Patients will be re-interviewed once by appointment: approximately 12 months after the outreach training started (12 to 16 months after recruitment). Recruitment will start in March 2011and will be staggered across the 38 trial clinics .

The trial co-ordinator will recruit fieldworkers from local community networks, and preference will be given to those with experience in community based surveys. Excellent knowledge of the community will be a pre-requisite to help find participants who do not return to the clinic for follow-up interviews. Fieldworkers will undergo a three to five day training covering: enrolment procedures and consent, measurement of blood pressure and other parameters, use of the handheld computer and general clinic staff liaison and interviewing skills.

## Data safety and monitoring plan

Day-to-day safety monitoring will be the responsibility of the study manager. In this she will be assisted by an independent safety officer who will review the blood results, and the fieldworkers performing blood pressure measurements and completing interviews. In the event that the pre-determined safety thresholds are breeched she will alert the study manager, who will contact the clinic staff to ensure that appropriate interventions are put in place. These thresholds and actions are summarised in the table below. Fieldworkers will be responsible for identifying patients with dangerously high blood pressure, and for referring them back to the clinician on duty for emergency care. Fieldworkers will also refer participants who become visibly emotionally distressed in response to the mental health components of the questionnaire. Referral letters to clinic staff have been piloted, and are included in Appendix 4. All safety monitoring will be uniformly implemented across intervention and control clinics so as not to bias the trial.

**Table 4. Safety monitoring of participants**

| **Parameter** | **Risk** | **Responsible for identifying dangerously raised values and acting on these** | **Threshold for intervention** | **Intervention** |
| --- | --- | --- | --- | --- |
| Blood pressure | Acute cardiovascular event e.g. stroke | Fieldworkers | Systolic blood pressure ≥ 180mmHg  Diastolic blood pressure ≥ 110mmHg | Refer back to clinician for review using structured referral letter |
| Psychological distress | Suicide | Fieldworkers | Visibly emotionally distressed during interview with limited recovery by end or volunteered suicidal thoughts during interview. | Refer back to clinician for review using structured referral letter |
| Triglycerides | Pancreatitis | Independent safety officer | ≥15mmol/l | Independent safety officer to alert study manager who will notify clinic staff and direct appropriate intervention |

An independent Data and Safety Monitoring Board is not considered necessary because this is not a Phase III trial, and all interventions contained in the guideline are known to be beneficial in the management of chronic diseases. Furthermore, all treatments are currently available at intervention and control clinics. We are only randomising an intervention that seeks to optimise their provision and established care for chronic diseases at these clinics.

The trial will be registered with the South African Clinical Trials Register and www.controlled-trials.com.

## Data analysis

The University of Cape Town Lung Institute will take primary responsibility for storing the data in electronic format. Electronic data will be uploaded to a password-protected sub-directory of a secure server. This network server is backed up daily and will meet the requirements of security and confidentiality of the data. The research data sets will not contain any personal or identification information. A link to identification information will be through a unique study number assigned to participants. Only the data architect and study manager will have access to named datasets, for linkage and quality control purposes.

All protocols, questionnaires, other data sheets, correspondence relating the ethical clearance for the relevant studies and hard copies of study information will be kept in locked cupboards at the project office at the University of Cape Town.

Blood will be analyzed at a private laboratory, Pathcare, accredited with SANAS (South African National Accreditation System) according to ISO 15189 standards. This laboratory has always adhered to international guidelines with regard to:

- - - 1. Control of specimen processing and results distribution,
      2. Documentation of standard operating procedures,
      3. Maintenance of equipment,
      4. Adherence to accepted laboratory safety procedures,
      5. Rapid turn-around times, and
      6. Monitoring and verification of results generated.

The Laboratory also participates in an external quality assurance program, the international Bio-Rad (previously Murex) Clinical Chemistry Quality Assessment Program. Relevant performance ratings for the analyses being used can be provided if required.

Efforts to ensure the accuracy and reliability of the data include: training and supervision of fieldworkers, including site visits during recruitment and patient interviews; implementation of automated skips, required fields, and range checks on the handheld interview programme; and routine quality reports on the database.

Data analysis plans are specific to each of the three studies and have been described in the relevant sections.

Three sets of mortality data are available to study the mortality patterns in South Africa.

1. Statistics South Africa provide unidentified public domain data with underlying cause of death based on vital registration within about 2 years of the event. The lowest level of geographic information is province.

2. Identified (ID number) data is available from the population register within about 3 months of the event with limited information on whether cause is natural or external. This data is restricted to deceased who are registered.

3. The Western Cape Provincial Government has extended a local level mortality surveillance system from 2 health districts to cover the province as of 2008. This will provide underlying cause of death as well as health sub-district and will be identifiable with ID. It will also include deceased who are not registered.

All mortality data will be stored by the Medical Research Council in keeping with existing agreements with the Department of Home Affairs and the Western Cape Department of Health.

## Description of risks and benefits

### Potential risks and protection from risk

The risks of these studies to the participants are minimal. The trial is not a Phase III trial where we are testing new treatments for chronic diseases. Rather we are evaluating whether chronic disease care and outcomes can be improved through the provision of an integrated guideline-based training program targeting nurses and doctors, and there is equipoise as to whether patients in either arm would be at greater risk. The guideline is based on the best available evidence and is consistent with national recommendations. Care will be provided by experienced health practitioners. The effects of the proposed research will be to randomly allocate implementation, and to provide training, managerial support and evaluation to ensure optimal implementation.

Participants may be newly identified as being at risk for an acute cardiovascular event, pancreatitis or even suicide through study procedures, namely blood pressure, triglyceride measurement and administration of the mental health components of the questionnaire. Management of such participants is outlined in Table 4.

There is also minor risk of discomfort, bruising and bleeding during or after phlebotomy, but this will be minimised by using only registered health practitioners to perform this procedure. Participants will be seated and standard infection control procedures will be enforced. All samples will be handled using strict criteria for the management of bodily fluids, including use of special sharps containers, special hazardous waste disposal units, alcohol and bleach cleansing of surfaces, use of latex gloves by all personnel, and occlusive covering of all puncture sites.

The usual care of participants will in no way be compromised, or jeopardized if a patient refuses to participate. Research procedures will take place after patients have received care at the clinic.

### Significance and potential benefit of research to participants and others

The trial is opportunistic and is proposed alongside initial incremental implementation of the integrated guideline-based training program in the Western Cape. Potential benefits are therefore related to the provision of training, managerial support and evaluation to ensure optimal implementation in all 520 primary care facilities in the province.

In resource-restricted settings like Southern Africa, new programmes are introduced at an opportunity cost to other priorities. This brings with it an ethical imperative to commit to open minded development of interventions, followed by experimental field testing that draws reliable conclusions about effectiveness and allows for ineffective or even harmful interventions to be dropped without delay. Introducing the integrated guideline-based training programme within the context of a pragmatic randomised controlled trial means that two ethical imperatives at programme level are met. Firstly the need to ensure that rising demand for treatment is met - equity; and secondly the Department of Health mandate to implement innovation in the presence of careful evaluation to ensure maintenance and improvement of quality - responsible stewardship over resources and protection of the population from harm.

If the pragmatic trial shows that the programme is effective, it suggests that implementation in other resource-restricted settings in South Africa and in the region will yield positive results. Results of the trial could be used to inform decision-making at a national and regional level. Within the Western Cape, the plan is to integrate the programme, if effective, with a component targeting community health workers, currently under development.

##

## Informed consent

Trained fieldworkers will be responsible for the consent process. Patients 18 years or older will be invited to participate in the studies in the waiting room, and a plain language information sheet will be provided, allowing them time to consider and discuss possible participation. Interested patients will be screened after consultation with the clinician, and in privacy, in an area of the clinic temporarily allocated to research staff.

Consent will be conducted in the language of the participant, and fieldworkers will be recruited accordingly. The screening process includes the identification of participants with impaired capacity to consent, whether because of acute illness, a psychiatric condition or severe cognitive impairment. These patients will be excluded from the study, as the study requires that participants actively engage in an interviewing process at the time of recruitment, and 12-15 months later.

Potential participants will be warned that only highly abnormal blood results will be brought to the clinic’s attention. This is because routine monitoring of HbA1C and lipids comprises a key guideline recommendation, and thus component of the training intervention. If all results were routinely returned, abnormal values may prompt clinicians to alter treatment, and confound measurement of the training’s effectiveness. Participants will be asked their permission to access hospitalisation, laboratory and death records through linkage using their South African identity number, folder numbers, and other identifiers.

A copy of the English version of the patient information sheet/ consent form is provided in Appendix 6.

Permission to perform the trial is being sought from the Western Cape Department of Health. The Department will also be asked to provide consent for patient-level data on hospitalisations and laboratory tests, drawn from routine databases, to contribute to the analysis.

## Privacy and confidentiality

To protect confidentiality, a unique number will be assigned to each participant. The identity number will not be linked to names or telephone numbers, except in a log which will be kept locked in the trial co-ordinator office. Identifiers will be destroyed after the data are entered into the computer and the data collection process is complete. Thereafter the only list with patient identifiers will be securely stored at the study offices.

## Reimbursement

Trial participants will be entitled to a food voucher worth one hundred rand (R100) which they will receive on the day of the follow up visit to the clinic to compensate for their participation in the study. Participating patients will also be reimbursed transport expenses in the form of a standard fee. It is hoped that the voucher will serve to incentivise participants to return to the clinic for the follow-up interview. This is necessary because of the long period between enrollment and follow-up (12-15 months) needed to detect differences in chronic disease care, characterised by infrequent (3-6 monthly) clinical review. Participants who do not return to the clinic for their interview will be followed-up at home, and receive their voucher once the interview has been completed. Should the fieldwork team determine that the participant has died at follow-up, the voucher will be passed onto their family.

## References

1. [Anderson KM, Odell PM, Wilson PW, Kannel WB.](http://www.ncbi.nlm.nih.gov/pubmed/1985385?ordinalpos=15&itool=EntrezSystem2.PEntrez.Pubmed.Pubmed_ResultsPanel.Pubmed_DefaultReportPanel.Pubmed_RVDocSum) Cardiovascular disease risk profiles. *Am Heart Jl*, 1991. **121**(1 Pt 2): 293-8.
2. Andresen EM, Malmgren JA, Carter WB, Patrick DL. Screening for depression in well older adults: evaluation of a short form of the CES-D (centre for Epidemiologic Studies Depression Scale). *American Journal Of Preventive Medicine*. 1994; 10(2): 77-84.
3. Armstrong Schellenberg JRM, Adam T, Mshinda H, Masanja H, Kabadi G, Mukasa O, John T, Charles S, Nathan R, Wilczynska K, Mgalula L, Mbuya C, Mswia R, Manzi F, de Savigny D, Schellenberg D, Victora C. Effectiveness and cost of facility-based Integrated Management of Child Illness (IMCI) in Tanzania. *Lancet.* 2004;**364**: 1583-1594.
4. Awad AI, Elyayeb IB, Baraka OZ. Changing antibiotic prescribing practices in health centres of Khartoum State, Sudan. *Eur J Clin Pharmacol.* 2005;**62**(2): 135-42.
5. [Bateman ED](http://www.ncbi.nlm.nih.gov/sites/entrez?Db=pubmed&Cmd=Search&Term=%22Bateman%20ED%22%5BAuthor%5D&itool=EntrezSystem2.PEntrez.Pubmed.Pubmed_ResultsPanel.Pubmed_DiscoveryPanel.Pubmed_RVAbstractPlus), [Feldman C](http://www.ncbi.nlm.nih.gov/sites/entrez?Db=pubmed&Cmd=Search&Term=%22Feldman%20C%22%5BAuthor%5D&itool=EntrezSystem2.PEntrez.Pubmed.Pubmed_ResultsPanel.Pubmed_DiscoveryPanel.Pubmed_RVAbstractPlus), [O'Brien J](http://www.ncbi.nlm.nih.gov/sites/entrez?Db=pubmed&Cmd=Search&Term=%22O'Brien%20J%22%5BAuthor%5D&itool=EntrezSystem2.PEntrez.Pubmed.Pubmed_ResultsPanel.Pubmed_DiscoveryPanel.Pubmed_RVAbstractPlus), [Plit M](http://www.ncbi.nlm.nih.gov/sites/entrez?Db=pubmed&Cmd=Search&Term=%22Plit%20M%22%5BAuthor%5D&itool=EntrezSystem2.PEntrez.Pubmed.Pubmed_ResultsPanel.Pubmed_DiscoveryPanel.Pubmed_RVAbstractPlus), [Joubert JR](http://www.ncbi.nlm.nih.gov/sites/entrez?Db=pubmed&Cmd=Search&Term=%22Joubert%20JR%22%5BAuthor%5D&itool=EntrezSystem2.PEntrez.Pubmed.Pubmed_ResultsPanel.Pubmed_DiscoveryPanel.Pubmed_RVAbstractPlus); [COPD Guideline Working Group of the South African Thoracic Society](http://www.ncbi.nlm.nih.gov/sites/entrez?Db=pubmed&Cmd=Search&Term=%22COPD%20Guideline%20Working%20Group%20of%20the%20South%20African%20Thoracic%20Society%22%5BCorporate%20Author%5D&itool=EntrezSystem2.PEntrez.Pubmed.Pubmed_ResultsPanel.Pubmed_DiscoveryPanel.Pubmed_RVAbstractPlus). Guideline for the management of chronic obstructive pulmonary disease (COPD): 2004 revision. [*S Afr Med J*.](javascript:AL_get(this,%20'jour',%20'S%20Afr%20Med%20J.');) 2004; **94**(7 Pt 2):559-75.
6. Bheekie A, Buskens I, Allen S, English R, Mayers P, Fairall L, Bateman ED, Zwarenstein M, Bachmann M. The Practical Approach to Lung Health in South Africa (PALSA) intervention: respiratory guideline implementation for nurse trainers. *Int Nurs Rev*. 2006;**53**(4):261-8.
7. Bradshaw D, Schneider M, Norman R, Bourne D. in K Steyn, J Fourie, N Tempel (eds). Chronic Diseases of Lifestyle in South Africa: 1995-2005. Technical Report. Cape Town: South African Medical Council. 2006
8. Bradshaw D, Norman R, Schneider M. A clarion call for action based on refined DALY estimates for South Africa. S Afr Med J 2007;97:438-440.
9. Buist SA, McBurnie MA, Vollmer WM, Gillespie S, Burney P, Mannino DM, Menezes AMB, Sullivan SD, Lee TA, Weiss KB, Gulsviv A, Nizankowska-Mogilnicka E, on behalf of the BOLD Collaborative. International variation in the prevalence of COPD (the BOLD Study): a population-based prevalence study. *Lancet* 2007; **370**:741-50.
10. Byatt, David, Ian Cooper, Chris Price. “40 Years of the Nelder-Mead Algorithm.” Presentation notes from the University of Canterbury, New Zealand. November 5^th^, 2003. Accessed from <http://64.233.169.104/search?q=cache:iTCfhECdKD0J:oldweb.cecm.sfu.ca/AAS/coope.pdf+nelder-mead+use&hl=en&ct=clnk&cd=2&gl=us&client=safar> on October 31, 2008.
11. Charlton K, Steyn K, Levitt NS, Peer N, Jonathan D, Gogela T, Rossouw K, Gwebushe N, Lombard CJ. A food-based dietary strategy lowers blood pressure in a low socio-economic setting: a randomised study in South Africa. Public Health Nutrition; 2008: 11(12), 1397–1406.
12. Cheung YB, Liu KY, Yip PSF. Performance of the CES-D and its Short Forms in Screening Suicidality and Hopelessness in the Community. *Suicide and Life-Threatening Behaviour.* 2007; 37(1): 79.
13. Chronic Diseases of Lifestyle in South Africa:1995-2005. Editors: Krisela Steyn, Jean Fourie, Norman Temple. Technical Report. Cape Town. South African Medical Research Council, 2006.
14. Churchyard GJ, Hnizdo E, White N 2001 Pulmonary Tuberculosis in Relation to Lung Function Loss. Safety in Mines Research Advisory Committee (SIMRAC) Research Report: Health 617. Johannesburg: SIMRAC
15. [Conroy RM, Pyörälä K, Fitzgerald AP, Sans S, Menotti A, De Backer G, De Bacquer D, Ducimetière P, Jousilahti P, Keil U, Njølstad I, Oganov RG, Thomsen T, Tunstall-Pedoe H, Tverdal A, Wedel H, Whincup P, Wilhelmsen L, Graham IM; SCORE project group.](http://www.ncbi.nlm.nih.gov/pubmed/12788299?ordinalpos=3&itool=EntrezSystem2.PEntrez.Pubmed.Pubmed_ResultsPanel.Pubmed_DefaultReportPanel.Pubmed_RVDocSum) Estimation of ten-year risk of fatal cardiovascular disease in Europe: the SCORE project*.* *Eur Heart J*, 2003. **24**(11):987-1003.
16. [D'Agostino RB Sr, Grundy S, Sullivan LM, Wilson P; CHD Risk Prediction Group.](http://www.ncbi.nlm.nih.gov/pubmed/11448281?ordinalpos=14&itool=EntrezSystem2.PEntrez.Pubmed.Pubmed_ResultsPanel.Pubmed_DefaultReportPanel.Pubmed_RVDocSum) Validation of the Framingham coronary heart disease prediction scores: results of a multiple ethnic groups investigation. *JAMA*, 2001. **286**(2):180-7.
17. Ehrlich R, White N, Norman R, Laubscher R, Steyn K, Lombard C, Bradshaw D. Predictors of chronic bronchitis in South African adults. *Int J Tuberc Lung Dis* 2004;8:369-376.
18. [El Arifeen S, Blum LS, Hoque DM, Chowdhury EK, Khan R, Black RE, Victora CG, Bryce J.](http://www.ncbi.nlm.nih.gov/pubmed/15519629?ordinalpos=4&itool=EntrezSystem2.PEntrez.Pubmed.Pubmed_ResultsPanel.Pubmed_DefaultReportPanel.Pubmed_RVDocSum) Integrated Management of Childhood Illness (IMCI) in Bangladesh: early findings from a cluster-randomised study. *Lancet.* 2004;**364**:1595-602.
19. Eltayeb IB, Awad AI, Mohamed-Salih MS, Daffa-Alla MA, Ahmed MB, Ogail MA, Matowe L. Changing the prescribing patterns of sexually transmitted infections in the White Nile Region of Sudan. *Sex Transm Inf.* 2005;**81**:426-427.
20. English RG, Bachmann MO, Bateman ED, Zwarenstein MF, Fairall LR, Bheekie A, Majara BP, Lombard C, Scherpbier R, Ottmani SE. Diagnostic accuracy of an integrated respiratory guideline in identifying patients with respiratory symptoms requiring screening for pulmonary tuberculosis: a cross-sectional study. *BMC Pulm Med.* 2006;**6**:22.
21. English RG, Fairall LR, Bateman ED. Keeping allergy on the agenda: using integrated care guidelines to improve the management of allergic conditions in high infectious disease burden settings. *Allergy.* 2007. **6**(3): 224-9.
22. Executive Summary: The policy implications of Tanzania’s mortality burden: vol 1 A ten year community based perspective: http//research.ncl.ac.uk/ammp/site_files/public_html
23. Fairall L, Zwarenstein M, Bateman ED, Bachmann OM, Lombard C, Majara B, Joubert G, English RG, Bheekie A, van Rensburg HCJ, Mayers P, Peters AC, Chapman RD. Educational outreach to nurses improves tuberculosis case detection and primary care of respiratory illness: a pragmatic cluster randomized controlled trial. *BMJ* 2005; **331**:750-754.
24. Fairall LR, Bachmann MO, Zwarenstein M, Bateman ED, Niessen L, Lombard C, Majara B, Joubert G, English RG, Bheekie A, van Rensburg D, Mayers P, Peters A, Chapman R. Cost effectiveness of educational outreach to primary care nurses to improve respiratory care: economic evaluation alongside a randomised trial. 2008ii. Submitted *Bull World Health Organ*.
25. Fairall LR, Bachmann MO, Zwarenstein MF, Lombard CJ, Uebel K, van Vuuren C, Steyn D, Boulle A, Bateman ED. Streamlining tasks and roles to expand treatment and care for HIV: randomised controlled trial protocol. *Trials.* 2008i;**9**:21.
26. Feder G, Griffiths C, Highton C, Eldridge S, Spence M, Southgate L. Do clinical guidelines introduced with practice based education improve care of asthmatic and diabetic patients? A randomised controlled trial in general practices in east London. *BMJ.* 1995;**311**:1473-78.
27. Gage BF, Cardinalli AB, Owens DK. Cost-effectiveness of preference-based antithrombotic therapy for patients with nonvalvular atrial fibrillation*.* *Stroke,* 1998. **29**(6):1083-91.
28. Gaziano TA, Steyn K, Cohen DJ, Weinstein MC, Opie LH. Cost-Effectiveness Analysis of Hypertension Guidelines in South Africa: Absolute Risk Versus Blood Pressure Level. *Circulation*. 2005;**112**:3569-3576.
29. Gaziano TA, Young CR, Fitzmaurice G, Atwood S, Gaziano JM. Laboratory-based versus non-laboratory-based method for assessment of cardiovascular disease risk: the NHANES I Follow-up Study cohort. *Lancet*. 2008;**371**:923-31
30. Gold MR, Siegel JE, Russell LB, Weinstein MC, ed. *Cost-effectiveness in Health and Medicine*. 1996, Oxford University Press: New York.
31. Gove S. Integrated management of childhood illness by outpatient health workers: technical basis and overview. *Bull World Health Org.*1997; **75**(Suppl): 7-24.
32. Grant R, Adams AS, Trinacty CM et al. Relationship Between Patient Medication Adherence and Subsequent Clinical Inertiain Type 2 Diabetes Glycemic Management. *Diabetes Care* 30:807–812, 2007.
33. Grimshaw J, Shirran L, Thomas R, Mowatt G, Fraser C, Bero L, Grilli R, Harvey E, Oxman A, O’Brien MA. Changing provider behaviour: an overview of systematic reviews of interventions. *Med Care.* 2001;**39** (Suppl 8): II2 – II45.
34. Grimshaw JM, Thomas RE, MacLennan G, Fraser C, Ramsay C, Vale L *et al*. Effectiveness and efficiency of guideline dissemination and implementation strategies. *Health Technol Assess*. 2004;**8**(6).
35. GRLS (Generalized Record Linkage System). Swiss National Cohort: Longitudinal study of people living in witzerland. Available online at: <http://www.ispm.ch/1134.html> [accessed 7 November 2008].
36. Grobbelaar JP, Bateman ED. Hut lung-a domestically acquired pneumoconiosis of mixed aetiology in rural women. *Thorax* 1991; **46**:334-340.
37. Groenewald P, Bradshaw D, Daniels J, et al. Cause of death and premature mortality in Cape Town, 2001-2006. Cape Town: South African Medical Research Council, 2008.
38. Grosskurth H, Mosha F, Todd J, *et al*. Impact of improved treatment of sexually transmitted diseases on HIV infection in rural Tanzania: randomised controlled trial. *Lancet.*1995; **346**: 530–36.
39. Guillemin F, Bombadier C, Beaton D. Cross-cultural adaptation of health-related quality of life measures: literature review and proposed guidelines. *J Clin Epidemiol.* 1993;**46**(12):1417-1432.
40. Haines A, Ashcroft R, Coggon D, *et al*. Personal Information in Medical Research. London: Medical Research Council 2000. [www.mrc.ac.uk](http://www.mrc.ac.uk)
41. Harding TW. Validating a method of psychiatric case identification in Jamaica. [Bull World Health Organ.](javascript:AL_get(this,%20'jour',%20'Bull%20World%20Health%20Organ.');) 1976;54(2):225-31.
42. [Harrison A, Karim SA, Floyd K, Lombard C, Lurie M, Ntuli N, Wilkinson D.](http://www.ncbi.nlm.nih.gov/entrez/query.fcgi?cmd=Retrieve&db=pubmed&dopt=Abstract&list_uids=11125896&query_hl=3&itool=pubmed_DocSum) Syndrome packets and health worker training improve sexually transmitted disease case management in rural South Africa: randomized controlled trial. *AIDS.* 2000; **14**(17):2769-79.
43. Ho PM, Magid DJ, Shetterly SM, Olson KL, Peterson PN, Masoudi FA, Rumsfeld JS. Importance of Therapy Intensification and Medication Nonadherence for Blood Pressure Control in Patients With Coronary Disease. Arch Intern Med. 2008;168(3):271-276.
44. Hnizdo E. Health risks among white South African goldminers - dust, smoking and chronic obstructive pulmonary disease. *S Afr Med J* 1992:**81**:512-517
45. Hunink MG, Goldman L, Tosteson AN, Mittleman MA, Goldman PA, Williams LW, Tsevat J, Weinstein MC. The recent decline in mortality from coronary heart disease, 1980-1990. The effect of secular trends in risk factors and treatment. *JAMA*. 1997;**277**:535-42.
46. Jamison D, Breman J, Measham A, Alleyne G, Cleason M, Evans D, Jha P, Mills A, Musgrove P. Disease Control Priorities in Developing Countries. 2nd ed. New York: Oxford University Press and The World Bank; 2006.
47. Jones PW, Quirk FH, Baveystock CM. The St George’s Respiratory Questionnaire. *Respiratory Medicine.* 1991; 85 (Supplement B): 25-31.
48. [Kim JJ, Kuntz KM, Stout NK, Mahmud S, Villa LL, Franco EL, Goldie SJ.](http://www.ncbi.nlm.nih.gov/pubmed/17526866?ordinalpos=88&itool=EntrezSystem2.PEntrez.Pubmed.Pubmed_ResultsPanel.Pubmed_DefaultReportPanel.Pubmed_RVDocSum) Multiparameter Calibration of a Natural History Model of Cervical Cancer. *Am J Epidemiol*. 2007;**166**(2):137-150.
49. [Kupersmith J](http://www.ncbi.nlm.nih.gov/sites/entrez?Db=pubmed&Cmd=Search&Term=%22Kupersmith%20J%22%5BAuthor%5D&itool=EntrezSystem2.PEntrez.Pubmed.Pubmed_ResultsPanel.Pubmed_DiscoveryPanel.Pubmed_RVAbstractPlus), [Holmes-Rovner M](http://www.ncbi.nlm.nih.gov/sites/entrez?Db=pubmed&Cmd=Search&Term=%22Holmes-Rovner%20M%22%5BAuthor%5D&itool=EntrezSystem2.PEntrez.Pubmed.Pubmed_ResultsPanel.Pubmed_DiscoveryPanel.Pubmed_RVAbstractPlus), [Hogan A](http://www.ncbi.nlm.nih.gov/sites/entrez?Db=pubmed&Cmd=Search&Term=%22Hogan%20A%22%5BAuthor%5D&itool=EntrezSystem2.PEntrez.Pubmed.Pubmed_ResultsPanel.Pubmed_DiscoveryPanel.Pubmed_RVAbstractPlus), [Rovner D](http://www.ncbi.nlm.nih.gov/sites/entrez?Db=pubmed&Cmd=Search&Term=%22Rovner%20D%22%5BAuthor%5D&itool=EntrezSystem2.PEntrez.Pubmed.Pubmed_ResultsPanel.Pubmed_DiscoveryPanel.Pubmed_RVAbstractPlus), [Gardiner J](http://www.ncbi.nlm.nih.gov/sites/entrez?Db=pubmed&Cmd=Search&Term=%22Gardiner%20J%22%5BAuthor%5D&itool=EntrezSystem2.PEntrez.Pubmed.Pubmed_ResultsPanel.Pubmed_DiscoveryPanel.Pubmed_RVAbstractPlus). Cost-effectiveness analysis in heart disease, Part II: Preventive therapies. *Prog Cardiovasc Dis*, 1995. **37**(4):243-71.
50. Leeder S, Raymond S, Greenberg H, Liu H, Esson K. A Race Against Time: The Challenge of Cardiovascular Disease in Developing Countries. New York: Trustees of Columbia University; 2004
51. Levitt, N.S., et al., The prevalence and identification of risk factors for NIDDM in urban Africans in Cape Town, South Africa. Diabetes Care, 1993. **16**(4): p. 601-7.
52. Levitt, N.S., et al., Modifiable risk factors for Type 2 diabetes mellitus in a peri-urban community in South Africa. Diabetic Medicine, 1999. **16**(11): p. 946-50.
53. [Lim SS](http://www.ncbi.nlm.nih.gov/sites/entrez?Db=pubmed&Cmd=Search&Term=%22Lim%20SS%22%5BAuthor%5D&itool=EntrezSystem2.PEntrez.Pubmed.Pubmed_ResultsPanel.Pubmed_DiscoveryPanel.Pubmed_RVAbstractPlus), [Gaziano TA](http://www.ncbi.nlm.nih.gov/sites/entrez?Db=pubmed&Cmd=Search&Term=%22Gaziano%20TA%22%5BAuthor%5D&itool=EntrezSystem2.PEntrez.Pubmed.Pubmed_ResultsPanel.Pubmed_DiscoveryPanel.Pubmed_RVAbstractPlus), [Gakidou E](http://www.ncbi.nlm.nih.gov/sites/entrez?Db=pubmed&Cmd=Search&Term=%22Gakidou%20E%22%5BAuthor%5D&itool=EntrezSystem2.PEntrez.Pubmed.Pubmed_ResultsPanel.Pubmed_DiscoveryPanel.Pubmed_RVAbstractPlus), [Reddy KS](http://www.ncbi.nlm.nih.gov/sites/entrez?Db=pubmed&Cmd=Search&Term=%22Reddy%20KS%22%5BAuthor%5D&itool=EntrezSystem2.PEntrez.Pubmed.Pubmed_ResultsPanel.Pubmed_DiscoveryPanel.Pubmed_RVAbstractPlus), [Farzadfar F](http://www.ncbi.nlm.nih.gov/sites/entrez?Db=pubmed&Cmd=Search&Term=%22Farzadfar%20F%22%5BAuthor%5D&itool=EntrezSystem2.PEntrez.Pubmed.Pubmed_ResultsPanel.Pubmed_DiscoveryPanel.Pubmed_RVAbstractPlus), [Lozano R](http://www.ncbi.nlm.nih.gov/sites/entrez?Db=pubmed&Cmd=Search&Term=%22Lozano%20R%22%5BAuthor%5D&itool=EntrezSystem2.PEntrez.Pubmed.Pubmed_ResultsPanel.Pubmed_DiscoveryPanel.Pubmed_RVAbstractPlus), [Rodgers A](http://www.ncbi.nlm.nih.gov/sites/entrez?Db=pubmed&Cmd=Search&Term=%22Rodgers%20A%22%5BAuthor%5D&itool=EntrezSystem2.PEntrez.Pubmed.Pubmed_ResultsPanel.Pubmed_DiscoveryPanel.Pubmed_RVAbstractPlus). Prevention of cardiovascular disease in high-risk individuals in low-income and middle-income countries: health effects and costs. *Lancet*, 2007. **370**(9604):2054-62.
54. [Liu J](http://www.ncbi.nlm.nih.gov/sites/entrez?Db=pubmed&Cmd=Search&Term=%22Liu%20J%22%5BAuthor%5D&itool=EntrezSystem2.PEntrez.Pubmed.Pubmed_ResultsPanel.Pubmed_DiscoveryPanel.Pubmed_RVAbstractPlus), [Hong Y](http://www.ncbi.nlm.nih.gov/sites/entrez?Db=pubmed&Cmd=Search&Term=%22Hong%20Y%22%5BAuthor%5D&itool=EntrezSystem2.PEntrez.Pubmed.Pubmed_ResultsPanel.Pubmed_DiscoveryPanel.Pubmed_RVAbstractPlus), [D'Agostino RB Sr](http://www.ncbi.nlm.nih.gov/sites/entrez?Db=pubmed&Cmd=Search&Term=%22D'Agostino%20RB%20Sr%22%5BAuthor%5D&itool=EntrezSystem2.PEntrez.Pubmed.Pubmed_ResultsPanel.Pubmed_DiscoveryPanel.Pubmed_RVAbstractPlus), [Wu Z](http://www.ncbi.nlm.nih.gov/sites/entrez?Db=pubmed&Cmd=Search&Term=%22Wu%20Z%22%5BAuthor%5D&itool=EntrezSystem2.PEntrez.Pubmed.Pubmed_ResultsPanel.Pubmed_DiscoveryPanel.Pubmed_RVAbstractPlus), [Wang W](http://www.ncbi.nlm.nih.gov/sites/entrez?Db=pubmed&Cmd=Search&Term=%22Wang%20W%22%5BAuthor%5D&itool=EntrezSystem2.PEntrez.Pubmed.Pubmed_ResultsPanel.Pubmed_DiscoveryPanel.Pubmed_RVAbstractPlus), [Sun J](http://www.ncbi.nlm.nih.gov/sites/entrez?Db=pubmed&Cmd=Search&Term=%22Sun%20J%22%5BAuthor%5D&itool=EntrezSystem2.PEntrez.Pubmed.Pubmed_ResultsPanel.Pubmed_DiscoveryPanel.Pubmed_RVAbstractPlus), [Wilson PW](http://www.ncbi.nlm.nih.gov/sites/entrez?Db=pubmed&Cmd=Search&Term=%22Wilson%20PW%22%5BAuthor%5D&itool=EntrezSystem2.PEntrez.Pubmed.Pubmed_ResultsPanel.Pubmed_DiscoveryPanel.Pubmed_RVAbstractPlus), [Kannel WB](http://www.ncbi.nlm.nih.gov/sites/entrez?Db=pubmed&Cmd=Search&Term=%22Kannel%20WB%22%5BAuthor%5D&itool=EntrezSystem2.PEntrez.Pubmed.Pubmed_ResultsPanel.Pubmed_DiscoveryPanel.Pubmed_RVAbstractPlus), [Zhao D](http://www.ncbi.nlm.nih.gov/sites/entrez?Db=pubmed&Cmd=Search&Term=%22Zhao%20D%22%5BAuthor%5D&itool=EntrezSystem2.PEntrez.Pubmed.Pubmed_ResultsPanel.Pubmed_DiscoveryPanel.Pubmed_RVAbstractPlus). Predictive value for the Chinese population of the Framingham CHD risk assessment tool compared with the Chinese Multi-Provincial Cohort Study*.* *JAMA* 2004. **291**(21):2591-9.
55. Masoli M, Fabian D, Holt S, Beasley R. The global burden of asthma: executive summary of the GINA Dissemination Committee report. *Allergy* 2004; **59**: 469–478.
56. Mayosi BM, Flisher AJ, Lalloo UM et al.The burden of non-communicable diseases in South Africa. Lancet. 2009;published online 25 August 2009. DOI;10.1016/S0140-6736(09)61306-4.
57. McMahon PM, Kong CY, Johnson BE et al. Estimating long-term effectiveness of lung cancer screening in the Mayo CT screening study*. Radiology* 2008 (in press).
58. Mendis S, Lindholm LH, Mancia G, Whitworth J, Alderman M, Lim S, Heagerty T. World Health Organization (WHO) and International Society of Hypertension (ISH) risk prediction charts: assessment of cardiovascular risk for prevention and control of cardiovascular disease in low and middle-income countries. *J Hypertens*, 2007. **25**(8):1578-82.
59. Milne FJ, Pinkney-Atkinson VJ. Southern African Hypertension Society Hypertension Guideline Working Groups, Hypertension guideline 2003 update*.* *S Afr Med J*, 2004. **94**(3 Pt 2): 209-16.
60. Mollentze WF, Moore A, Steyn AF, Joubert G, Steyn K, Oosthuizen GM, Weich DJV. Coronary heart disease risk factors in a rural and urban Orange Free State black population. *S Afr Med J.* 1995; **85(2):** 90-96.
61. Morrell CJ, Slade P, Warner R et al. Clinical effectiveness of health visitor training in psychologically informed approaches for depression in postnatal women: pragmatic cluster randomised trial in primary care. BMJ 2009;338:a3045 doi:10.1136/bmj.a3045.
62. Myer L, Stein DJ, Grimsrud A, Seedat S, Williams DR (2008) Social determinants of psychological distress in a nationally-representative sample of South African adults. Social Science & Medicine 66:1828-1840.
63. National Health Committee, Guidelines for the management of mildly raised blood pressure in New Zealand. 1995, Core Services Committee, Ministry of Health: Wellington, New Zealand.
64. Nau D, Garber MC, Herman WH. The Intensification of Drug Therapy for Diabetes and Its Complications:Evidence From 2 HMOs. Am J Manag Care. 2004;10(part 2):118-123.
65. Nelder JA, Mead R. A Simplex Method for Function Minimization. *The Computer J*. 1965;**7**:308-313.
66. Oelofse A, Jooste P, *Steyn K*, Badenhorst CJ, Bourne LT, J Fourie. The lipid and lipoprotein profile of the urban black South African Community of the Cape Peninsula - the BRISK Study. *S Afr Med J.* 1996; **86:** 166-169.
67. Norman R, Bradshaw D, Schnedier M et al and the SA CRA Collaborating Group. A comparative risk assessment for South Africa in 2000: towards promoting health and preventing disease. S Afr Med J 2007;97:637-641.
68. O’Connor P, Desai J, Solberg LI et al. Randomized Trial of Quality Improvement Intervention to Improve
69. Diabetes Care in Primary Care Settings. *Diabetes Care* 28:1890–1897, 2005.
70. Oxman A, Thomson MA, Davis DA, Haynes RB. No magic bullets: a systematic review of 102 trials of interventions to improve professional practice. *CMAJ.* 1995; **153**:1423–31.
71. Oxman AD, Flottorp S. An overview of strategies to promote implementation of evidence based health care. In Silagy C, Haines A (eds.). *Evidence Based Practice*. 2^nd^ Edition. 2001. London: BMJ Publishers.
72. Pagaiya N, Garner P. Primary care nurses using guidelines in Thailand: a randomised controlled trial. *Trop Med Int Health.* 2005;**10**(5):471-77.
73. [Pattinson RC](http://www.ncbi.nlm.nih.gov/entrez/query.fcgi?db=pubmed&cmd=Search&itool=pubmed_Abstract&term=%22Pattinson+RC%22%5BAuthor%5D), [Arsalo I](http://www.ncbi.nlm.nih.gov/entrez/query.fcgi?db=pubmed&cmd=Search&itool=pubmed_Abstract&term=%22Arsalo+I%22%5BAuthor%5D), [Bergh AM](http://www.ncbi.nlm.nih.gov/entrez/query.fcgi?db=pubmed&cmd=Search&itool=pubmed_Abstract&term=%22Bergh+AM%22%5BAuthor%5D), [Malan AF](http://www.ncbi.nlm.nih.gov/entrez/query.fcgi?db=pubmed&cmd=Search&itool=pubmed_Abstract&term=%22Malan+AF%22%5BAuthor%5D), [Patrick M](http://www.ncbi.nlm.nih.gov/entrez/query.fcgi?db=pubmed&cmd=Search&itool=pubmed_Abstract&term=%22Patrick+M%22%5BAuthor%5D), [Phillips N](http://www.ncbi.nlm.nih.gov/entrez/query.fcgi?db=pubmed&cmd=Search&itool=pubmed_Abstract&term=%22Phillips+N%22%5BAuthor%5D). Implementation of kangaroo mother care: a randomized trial of two outreach strategies. [*Acta Paediatr.*](javascript:AL_get(this,%20'jour',%20'Acta%20Paediatr.');) 2005;**94**(7):924-7.
74. Pickering T, Hall JE, Appel LJ, Falkner BE, Graves J, Hill M, Jones DW, Kurtz T, Sheldon G, Roccella EJ.Recommendations for Blood Pressure Measurement in Humans and Experimental Animals: Part 1: Blood Pressure Measurement in Humans. *Hypertension* 2005; 45;142-161.
75. Peer N, Steyn K, Dennison C, Nel H, Hi l MN. Determinants of target organ damage in black hypertensives attending primary care in Cape Town; the Hi-Hi study*. American* *J Hyperten* (Epub 2008 Jun )
76. Pestana JA, Steyn K, Leiman A, Hartzenberg GM. The direct and indirect costs of cardiovascular disease in South Africa in 1991. *S Afr Med J*. 1996 ;**86**:679-84.
77. Posl M, Cieza A, Stucki G. psychometric properties of the WHODASII in rehabilitation patients. *Qual Life Res.* 2007; 16: 1521-1531.
78. Poyser MA, Nelson H, Ehrlich RI, Bateman ED, Parnell S, Puterman A, Weinberg E. Socioeconomic deprivation and asthma prevalence and severity in young adolescents. *Eur Resp J*. 2002; **19**:892-8.
79. Prince M, Patel V, Saxena S, et al. No health without mental health. Lancet 2007; 370:859-877.
80. Prochaska JO, DiClemente CC. Towards a comprehensive model of change. In: Miller WR, Heather N, eds. *Treating addictive behaviours: Processes of change.* New York: Plenum Press. 1986.
81. Rodgers A, Lawes C, Gaziano T, Vos T. The Growing Burden of Risk from High Blood Pressure, Cholesterol, and Bodyweight, in Disease Control Priorities in Developing Countries, D. Jamison, Editor. 2006, Oxford University Press and the World Bank: New York.
82. Radloff LS. The CES-D Scale: A Self-Report Depression Scale for Research in the General Population. *Applied Psychological Measurement.*  1977; 1 (3): 385-401.
83. Rahman A, Malik A, Sikander S, Roberts C, Creed F. Cognitive behaviour therapy-based intervention by community health workers for mothers with depression and their infants in rural Pakistan: a cluster-randomised controlled trial. *Lancet*. 2008 September 13; 372(9642): 902–909.
84. Rodondi N, Peng T, Karter AJ, Bauer DC, Vittinghoff E, Tang S, Pettitt D, Kerr EA, Selby JV. Therapy Modifications in Response to Poorly Controlled Hypertension, Dyslipidemia, and Diabetes Mellitus. *Ann Intern Med.* 2006;144:475-484.
85. Rosman KD., The epidemiology of stroke in an urban black population*.* *Stroke,* 1986;**17**(4):667-9.
86. Ross-Degnan D, Soumerai SB, Goel PK, Bates J, Makhulo J, Dondi N, Sutoto, Adi D, Ferraz-Tabor L, Hogan R. The impact of face-to-face educational outreach on diarrhoea treatment in pharmacies. *Health Policy Plan.* 1996; **11**(3): 308-18.
87. Santoso B. Small group intervention vs. formal seminar for improving appropriate drug use. *Soc Sci Med*. 1996; **42**(8): 1163-1168.
88. Schwartz D, Lellouch J. Explanatory and pragmatic attitudes in therapeutic trials. *J Chronic Dis.* 1967;**20**(8):637-48.
89. Seebregts C, Zwarenstein M, Seebregts C, Young P, Sheck S, Fairall L, Klepp KI, Flisher A, Mukoma W, Ahmed N, Jansen S and Mathews C. Handheld Computers Improve Survey and Trial Data Collection in Resource-Poor Settings: Development and Evaluation of P-HAPI, a Palm™ Pilot Interviewing System. *Int J Med Inform*. 2008. In press.
90. [Seedat YK, Croasdale MA, Milne FJ, Opie LH, Pinkney-Atkinson VJ, Rayner BL, Veriava Y; Guideline Committee, Southern African Hypertension Society; Directorate: Chronic Diseases, Disabilities and Geriatrics, National Department of Health.](http://www.ncbi.nlm.nih.gov/pubmed/16670808?ordinalpos=6&itool=EntrezSystem2.PEntrez.Pubmed.Pubmed_ResultsPanel.Pubmed_DefaultReportPanel.Pubmed_RVDocSum) South African hypertension guideline 2006. *S Afr Med J*. 2006. **96**(4 Pt 2):337-62.
91. Statistics South Africa. Mortality and causes of death in South Africa, 1997-2003. Findings from death notification. Statistical release P0309.3. Pretoria: Statistics South Africa 2005.
92. Stein J, Lewin S, Fairall L, Mayers P, English R, Bheekie A, Bateman E, Zwarenstein M. Building capacity for antiretroviral delivery in South Africa: A qualitative evaluation of the Practical Approach to Lung Health and HIV/AIDS (PALSA Plus) nurse training. *BMC Health Services Research*. 2008. In press.
93. Stein J, Lewin S, Fairall L. Hope is the pillar of the universe: Health-care providers’ experiences of delivering anti-retroviral therapy in primary health-care clinics in the Free State province of South Africa. *Soc Sci & Med.* 2007;**64**: 954–964.
94. Steyn K, Bradshaw D, Norman R, Laubscher R. Determinants and Treatment of Hypertension in South Africans during 1998. The first Demographic and Health Survey. *S Afr Med J* 2008;98:376-380.(ii)
95. Steyn K, Fourie J, Benadé AJS, Rossouw JE, Langenhoven ML, Joubert G, Charlton DO. Factors associated with high density lipoprotein cholesterol in a population with high HDL-cholesterol levels. *Arteriosclerosis.* 1989; **9:** 390-397.
96. Steyn, K., et al., Hypertension in South African adults: results from the Demographic and Health Survey, 1998. J Hypertens, 2001. **19**(10): p. 1717-25.
97. Steyn K, Levitt NS, Patel M, Fourie JM, Gwebushe N, Lombard C, Everett K. Hypertension and diabetes: Poor care for patients at community health centres. *S Afr Med J* 2008; 98(8): 618-622 (i)
98. Steyn K, Levitt NS. Health Services Research in South Africa for Chronic Diseases of Lifestyle. In ‘*Chronic Diseases of Lifestyle in South Africa:1995-2005.* Editors: Krisela Steyn, Jean Fourie, Norman Temple. Technical Report. Cape Town. South African Medical Research Council, 2006
99. Steyn K, Sliwa-Hahnle K, Hawkens S, Commerford P, Onin C, Ounpuu S, Yusuf S. Risk factors associated with Myocardial Infarction in Africa. The INTERHEART Africa Study. *Circulation* 2005;112(23):3554-3561.
100. Taylor DCA, Kruzikas DT, Pandya A, Iskandar R, Gilmore KE, Weinstein MC. “Methods of Model Calibration: A Comparative Approach”, podium presentation at International Society for Pharmacoeconomics and Outcomes Research (ISPOR) 12th Annual International Meeting, Arlington, May 2007, Presentation MC1
101. Thomson MA, Oxman AD, Davis DA et al. Outreach visits to improve health professional practice and health care outcomes (Cochrane Review). In: *The Cochrane Library*, Issue 1. Oxford: Update software. 1999.
102. Tunis SR, Stryer DB, Clancy CM. Practical clinical trials: increasing the value of clinical research for decision-making in clinical and health policy. *JAMA*. 2004;**291**(4):425-6.
103. Turchin A, Shubina M, Chodos AH, Einbinder JS, Pendergrass ML. Effect of Board Certification on Antihypertensive Treatment Intensification in Patients With Diabetes Mellitus. *Circulation* 2008;117;623-628.
104. Unwin N, Setel P, Serf R, Mungusi F, Mbanya JC, Kitanga H et.al. Noncommunicable diseases in Sub-Saharan Africa: where do they feature in the health research agenda? *Bull World Health Org*.2001;79(10)):947-953.
105. van Bruggen, Gorter K, Stolk R, Klungel O, Rutten G. Clinical inertia in general practice: widespread and related to the outcome of diabetes care. Family Practice 2009; 26: 428–436.
106. Victora CG, Hanson K, Bryce J, Vaughan JP. Achieving universal coverage with health interventions. *Lancet.* 2004;**364**:1541-1548.
107. Wang R, Alexander GC, Stafford RS. Outpatient Hypertension Treatment,Treatment Intensification, and Control in Western Europe and the United States Arch Intern Med. 2007;167:141-147.
108. Wawer MJ, Sewankambo N, Serwadda D, et al. Control of sexually transmitted diseases for AIDS prevention in Uganda: a randomized community trial. *Lancet.* 1999;**353**: 525–35.
109. [Weinstein MC](http://www.ncbi.nlm.nih.gov/sites/entrez?Db=pubmed&Cmd=Search&Term=%22Weinstein%20MC%22%5BAuthor%5D&itool=EntrezSystem2.PEntrez.Pubmed.Pubmed_ResultsPanel.Pubmed_DiscoveryPanel.Pubmed_RVAbstractPlus), [Coxson PG](http://www.ncbi.nlm.nih.gov/sites/entrez?Db=pubmed&Cmd=Search&Term=%22Coxson%20PG%22%5BAuthor%5D&itool=EntrezSystem2.PEntrez.Pubmed.Pubmed_ResultsPanel.Pubmed_DiscoveryPanel.Pubmed_RVAbstractPlus), [Williams LW](http://www.ncbi.nlm.nih.gov/sites/entrez?Db=pubmed&Cmd=Search&Term=%22Williams%20LW%22%5BAuthor%5D&itool=EntrezSystem2.PEntrez.Pubmed.Pubmed_ResultsPanel.Pubmed_DiscoveryPanel.Pubmed_RVAbstractPlus), [Pass TM](http://www.ncbi.nlm.nih.gov/sites/entrez?Db=pubmed&Cmd=Search&Term=%22Pass%20TM%22%5BAuthor%5D&itool=EntrezSystem2.PEntrez.Pubmed.Pubmed_ResultsPanel.Pubmed_DiscoveryPanel.Pubmed_RVAbstractPlus), [Stason WB](http://www.ncbi.nlm.nih.gov/sites/entrez?Db=pubmed&Cmd=Search&Term=%22Stason%20WB%22%5BAuthor%5D&itool=EntrezSystem2.PEntrez.Pubmed.Pubmed_ResultsPanel.Pubmed_DiscoveryPanel.Pubmed_RVAbstractPlus), [Goldman L](http://www.ncbi.nlm.nih.gov/sites/entrez?Db=pubmed&Cmd=Search&Term=%22Goldman%20L%22%5BAuthor%5D&itool=EntrezSystem2.PEntrez.Pubmed.Pubmed_ResultsPanel.Pubmed_DiscoveryPanel.Pubmed_RVAbstractPlus). Forecasting Coronary Heart Disease Incidence, Mortality, and Cost: The Coronary Heart Disease Policy Model*. Am J Public Health* 1987; 77:1417-1426.
110. [Weinstein MC](http://www.ncbi.nlm.nih.gov/sites/entrez?Db=pubmed&Cmd=Search&Term=%22Weinstein%20MC%22%5BAuthor%5D&itool=EntrezSystem2.PEntrez.Pubmed.Pubmed_ResultsPanel.Pubmed_DiscoveryPanel.Pubmed_RVAbstractPlus), [O'Brien B](http://www.ncbi.nlm.nih.gov/sites/entrez?Db=pubmed&Cmd=Search&Term=%22O'Brien%20B%22%5BAuthor%5D&itool=EntrezSystem2.PEntrez.Pubmed.Pubmed_ResultsPanel.Pubmed_DiscoveryPanel.Pubmed_RVAbstractPlus), [Hornberger J](http://www.ncbi.nlm.nih.gov/sites/entrez?Db=pubmed&Cmd=Search&Term=%22Hornberger%20J%22%5BAuthor%5D&itool=EntrezSystem2.PEntrez.Pubmed.Pubmed_ResultsPanel.Pubmed_DiscoveryPanel.Pubmed_RVAbstractPlus), [Jackson J](http://www.ncbi.nlm.nih.gov/sites/entrez?Db=pubmed&Cmd=Search&Term=%22Jackson%20J%22%5BAuthor%5D&itool=EntrezSystem2.PEntrez.Pubmed.Pubmed_ResultsPanel.Pubmed_DiscoveryPanel.Pubmed_RVAbstractPlus), [Johannesson M](http://www.ncbi.nlm.nih.gov/sites/entrez?Db=pubmed&Cmd=Search&Term=%22Johannesson%20M%22%5BAuthor%5D&itool=EntrezSystem2.PEntrez.Pubmed.Pubmed_ResultsPanel.Pubmed_DiscoveryPanel.Pubmed_RVAbstractPlus), [McCabe C](http://www.ncbi.nlm.nih.gov/sites/entrez?Db=pubmed&Cmd=Search&Term=%22McCabe%20C%22%5BAuthor%5D&itool=EntrezSystem2.PEntrez.Pubmed.Pubmed_ResultsPanel.Pubmed_DiscoveryPanel.Pubmed_RVAbstractPlus), [Luce BR](http://www.ncbi.nlm.nih.gov/sites/entrez?Db=pubmed&Cmd=Search&Term=%22Luce%20BR%22%5BAuthor%5D&itool=EntrezSystem2.PEntrez.Pubmed.Pubmed_ResultsPanel.Pubmed_DiscoveryPanel.Pubmed_RVAbstractPlus) Principles of Good Practice for Decision Analytic Modeling in Health-Care Evaluation: Report of the ISPOR Task Force on Good Research Practices—Modeling Studies. *Value in Health*. January 2003;6(1):9-17.
111. Williams Dr, Herman A, Stein DJ et al. Twelve-month mental disorders in South Africa: prevalence, service use, and demographic correlates in the population-based South African stress and health study. Psychol Med. 2008;38;211-220.
112. WHO. Macroeconomics and Health: Investing in Health for Economic Development-Report of the Commission on Macroeconomics and Health. In. Geneva:: World Health Organization; 2001.
113. WHO. The global burden of disease: 2004 update. Geneva: World Health Organisation, 2006.
114. WHO, Prevention of Cardiovascular Disease. Guidelines for the assessment and management of total cardiovascular risk*.*. 2007, World Health Organization: Geneva.
115. Working Group South African Thoracic Society. South African Thoracic Society 2007 Guidelines for the management of chronic asthma in adolescents and adults. *S Afr Fam Prac.* 2007; **49**(5): 19-31.
116. World Bank, *World Development Indicators*. 2002, Washington, DC: The World Bank.
117. World Health Organization, *Preventing Chronic Diseases: a vital investment. WHO Global Report.* 2005 WHO: Geneva.
118. World Health Organization. *IMAI (Integrated management of Adolescent and Adult Illness) Fact Sheet.* 2003; Geneva: World Health Organization. Available from: <http://www.who.int/3by5/capacity/fs/en/index.html> [cited 15 April 2006].
119. Williamson JD, Miller ME, Bryan RN, Lazar RM, Coker LH, Johnson J, Cukierman T, Horowitz KR, Murray A, Launer LJ; ACCORD Study Group. [The Action to Control Cardiovascular Risk in Diabetes Memory in Diabetes Study (ACCORD-MIND): rationale, design, and methods.](http://www.ncbi.nlm.nih.gov/pubmed/17599421)Am J Cardiol. 2007; 99(12A):112i-122i.
120. World Health Organization. Innovative Care for Chronic Conditions: Building Blocks for action. Geneva: World Health Organisation, 2002.
121. World Health Organization. 2001b. Practical Approach to Lung Health – Guidelines for First-Level Facility Health Workers. Draft 14. Geneva: WHO/ CDS Stop TB Programme.
122. World Health Organization. Global Strategy on Diet, Physical Activity and Health. Geneva : World Health Organization, 2004.
123. World Health Organization. Integrated management of Childhood Illness: IMCI Adaptation Guide*.* 2002; Geneva: World Health Organization. Available from: <http://www.who.int/child-adolescent-health/New_Publications/IMCI/IMCI_Adaptation_Guige/sectiona.pdf> [cited 15 April 2006].
124. World Health Organization. Management of patients with sexually transmitted diseases. Report of a WHO Study Group. (WHO Technical Report Series, No. 810). 1991;Geneva: World Health Organization.
125. World Health Organization. Practical Approach to Lung Health (PAL): a primary healthcare strategy for the integrated management of respiratory conditions in people five years of age and over. WHO/HTM/TB/2005.351. 2005; Geneva: World Health Organization. Available from: <http://whqlibdoc.who.int/hq/2005/WHO_HTM_TB_2005.351.pdf> [cited 15 April 2006].
126. World Health Organization. Prevention and control of noncommunicable diseases: implementation of the global strategy. Report by the Secretariat. 18 April 2008: Geneva.
127. World Health Organization. Prevention of cardiovascular disease: pocket guidelines for assessment and management of cardiovascular risk: (WHO/ISH cardiovascular risk prediction charts for the African Region). Geneva, 2007.
128. [Yeh JM, Kuntz KM, Ezzati M, Hur C, Kong CY, Goldie SJ.](http://www.ncbi.nlm.nih.gov/pubmed/18483340?ordinalpos=5&itool=EntrezSystem2.PEntrez.Pubmed.Pubmed_ResultsPanel.Pubmed_DefaultReportPanel.Pubmed_RVDocSum) Development of an Empirically Calibrated Model of Gastric Cancer in Two High-Risk Countries. *Cancer Epidemiol Biomarkers Prev* 2008;17(5):1179-87.
129. [Yusuf S](http://www.ncbi.nlm.nih.gov/sites/entrez?Db=pubmed&Cmd=Search&Term=%22Yusuf%20S%22%5BAuthor%5D&itool=EntrezSystem2.PEntrez.Pubmed.Pubmed_ResultsPanel.Pubmed_DiscoveryPanel.Pubmed_RVAbstractPlus), [Hawken S](http://www.ncbi.nlm.nih.gov/sites/entrez?Db=pubmed&Cmd=Search&Term=%22Hawken%20S%22%5BAuthor%5D&itool=EntrezSystem2.PEntrez.Pubmed.Pubmed_ResultsPanel.Pubmed_DiscoveryPanel.Pubmed_RVAbstractPlus), [Ounpuu S](http://www.ncbi.nlm.nih.gov/sites/entrez?Db=pubmed&Cmd=Search&Term=%22Ounpuu%20S%22%5BAuthor%5D&itool=EntrezSystem2.PEntrez.Pubmed.Pubmed_ResultsPanel.Pubmed_DiscoveryPanel.Pubmed_RVAbstractPlus), [Dans T](http://www.ncbi.nlm.nih.gov/sites/entrez?Db=pubmed&Cmd=Search&Term=%22Dans%20T%22%5BAuthor%5D&itool=EntrezSystem2.PEntrez.Pubmed.Pubmed_ResultsPanel.Pubmed_DiscoveryPanel.Pubmed_RVAbstractPlus), [Avezum A](http://www.ncbi.nlm.nih.gov/sites/entrez?Db=pubmed&Cmd=Search&Term=%22Avezum%20A%22%5BAuthor%5D&itool=EntrezSystem2.PEntrez.Pubmed.Pubmed_ResultsPanel.Pubmed_DiscoveryPanel.Pubmed_RVAbstractPlus), [Lanas F](http://www.ncbi.nlm.nih.gov/sites/entrez?Db=pubmed&Cmd=Search&Term=%22Lanas%20F%22%5BAuthor%5D&itool=EntrezSystem2.PEntrez.Pubmed.Pubmed_ResultsPanel.Pubmed_DiscoveryPanel.Pubmed_RVAbstractPlus), [McQueen M](http://www.ncbi.nlm.nih.gov/sites/entrez?Db=pubmed&Cmd=Search&Term=%22McQueen%20M%22%5BAuthor%5D&itool=EntrezSystem2.PEntrez.Pubmed.Pubmed_ResultsPanel.Pubmed_DiscoveryPanel.Pubmed_RVAbstractPlus), [Budaj A](http://www.ncbi.nlm.nih.gov/sites/entrez?Db=pubmed&Cmd=Search&Term=%22Budaj%20A%22%5BAuthor%5D&itool=EntrezSystem2.PEntrez.Pubmed.Pubmed_ResultsPanel.Pubmed_DiscoveryPanel.Pubmed_RVAbstractPlus), [Pais P](http://www.ncbi.nlm.nih.gov/sites/entrez?Db=pubmed&Cmd=Search&Term=%22Pais%20P%22%5BAuthor%5D&itool=EntrezSystem2.PEntrez.Pubmed.Pubmed_ResultsPanel.Pubmed_DiscoveryPanel.Pubmed_RVAbstractPlus), [Varigos J](http://www.ncbi.nlm.nih.gov/sites/entrez?Db=pubmed&Cmd=Search&Term=%22Varigos%20J%22%5BAuthor%5D&itool=EntrezSystem2.PEntrez.Pubmed.Pubmed_ResultsPanel.Pubmed_DiscoveryPanel.Pubmed_RVAbstractPlus), [Lisheng L](http://www.ncbi.nlm.nih.gov/sites/entrez?Db=pubmed&Cmd=Search&Term=%22Lisheng%20L%22%5BAuthor%5D&itool=EntrezSystem2.PEntrez.Pubmed.Pubmed_ResultsPanel.Pubmed_DiscoveryPanel.Pubmed_RVAbstractPlus); [INTERHEART Study Investigators](http://www.ncbi.nlm.nih.gov/sites/entrez?Db=pubmed&Cmd=Search&Term=%22INTERHEART%20Study%20Investigators%22%5BCorporate%20Author%5D&itool=EntrezSystem2.PEntrez.Pubmed.Pubmed_ResultsPanel.Pubmed_DiscoveryPanel.Pubmed_RVAbstractPlus). Effect of potentially modifiable risk factors associated with myocardial infarction in 52 countries (the INTERHEART study): case-control study*.* *Lancet*, 2004. **364**(9438):937-52.
130. Zar HJ, Stickells D, Toerien A, Wilson D, Klein M, Bateman ED. Changes in fatal and near-fatal asthma in an urban area of South Africa from 1980 – 1997. *Eur Resp J* 2001; 18:33-37.
131. Zwarenstein M, Fairall LR and Lombard C for the PALSA PLUS STUDY group: Mayers P, Bheekie A, English RG, Stein J, Lewin S, Tsibolane Y, Shai-Mahtu P, Bachmann M, Bateman ED. Integration through outreach education improves adult HIV/AIDS and respiratory primary care: the PALSA PLUS pragmatic cluster randomised trial. 2008i. Submitted *NEJM.*
132. Zwarenstein M, Treweek S, Altman D, Gagnier J, Tunis S, Haynes BR, Oxman AD, Moher D. Improving the reporting of pragmatic trials: an extension of the CONSORT. *BMJ*. 2008ii. *In press.*

## Appendix 1: Amended List of participating facilities

| **No.** | **Subdistrict** | **Clinic** |
| --- | --- | --- |
| 1 | Bitou | Crags Clinic |
| 2 | Bitou | Kranshoek Clinic |
| 3 | Bitou | Kwanokathula Clinic |
| 4 | Bitou | New Horizon Clinic |
| 5 | Bitou | Plettenberg Bay CDC |
| 6 | EDA | Haarlem Clinic |
| 7 | EDA | Uniondale (Lyonsville) Clinic |
| 8 | George | Thembalethu CDC |
| 9 | George | Blanco Clinic |
| 10 | George | Conville CDC |
| 11 | George | George Civic Centre |
| 12 | Overberg | Barrydale Clinic |
| 13 | George | Pacaltsdorp Clinic |
| 14 | Overberg | Bredasdorp Clinic |
| 15 | Overberg | Napier Clinic |
| 16 | Hessequ | Riversdale Clinic |
| 17 | Hessequ | Albertinia Clinic |
| 18 | Hessequ | Heidelberg Clinic |
| 19 | Kannalan | Calitzdorp (Bergsig) |
| 20 | Kannalan | Ladismith (Nissenville) Clinic |
| 21 | Kannalan | Zoar Clinic |
| 22 | Knysna | Hornlee Clinic |
| 23 | Knysna | Keurhoek Clinic |
| 24 | Knysna | Khayelethu Clinic |
| 25 | Knysna | Knysna Town Clinic |
| 26 | Knysna | Sedgefield Clinic |
| 27 | Knysna | Wit Lokasie Clinic |
| 28 | MosselBay | Alma Clinic |
| 29 | MosselBay | D'Almeida Clinic |
| 30 | MosselBay | Eyethu Clinic |
| 31 | MosselBay | Great Brak River Clinic |
| 32 | Oudtshoorn | Bongolethu Clinic |
| 33 | Oudtshoorn | Bridgeton CDC |
| 34 | Oudtshoorn | De Rust (Blommenek) Clinic |
| 35 | Oudtshoorn | Dysselsdorp Clinic |
| 36 | Oudtshoorn | Oudtshoorn Clinic |
| 37 | Overberg | Swellendam Clinic |
| 38 | Overberg | Railton Clinic |

Appendix 2: Referral letter for very high blood pressure (revised title of letter)

**URGENT REFERRAL LETTER TO NURSE/ DOCTOR FOR**

**VERY HIGH BLOOD PRESSURE**


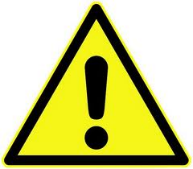


To Whom It May Concern

Re: …………………………………………………….. (Patient’s name and age)

This patient participated in a study that we are conducting on chronic disease care.

He/she was found to have a raised blood pressure ……….mm/Hg

Would you kindly review their blood pressure treatment

………………………………………………………… …………………………………….

Signature of Principal Investigator or delegate Date

………………………………………………………….……………………………………………………………

Printed name of Principal Investigator or delegate

Appendix 3: Referral letter for undiagnosed hypertension (new referral letter)

**REFERRAL LETTER TO NURSE/ DOCTOR FOR**

**UNDIAGNOSED BLOOD PRESSURE**

To Whom It May Concern

Re: …………………………………………………….. (Patient’s name and age)

This patient participated in a study that we are conducting on chronic disease care.

He/she was found to have a raised blood pressure of ……………….mm/Hg but has not reported being on medication for hypertension.

Would you kindly review their blood pressure.

………………………………………………………… …………………….

Signature of Principal Investigator or delegate Date

………………………………………………………….…………………………………

Printed name of Principal Investigator or delegate

Appendix 4: Referral letter for participants visibly distressed emotionally and/or with suicidal ideation (this combined letter replaces the baseline letter for distressed participants)

**URGENT REFERRAL LETTER TO NURSE/ DOCTOR**

To Whom It May Concern

Re: …………………………………………………….. (Patient’s name and age)

I interviewed the above patient today for a study that we are conducting on chronic disease care.

The interview contained questions about emotional wellbeing. During the interview, he/she became visibly distressed and/or answered ‘yes’ to the following question: ‘Has the thought of ending your life been on your mind’.

I am concerned about his/her mental well being and would be grateful if you could assess his/her mental state and manage accordingly.

Many thanks for your assistance in this matter.

Yours sincerely

………………………………………………………… ………………………

Signature of Principal Investigator or delegate Date

………………………………………………………………………………………………………

Printed name of Principal Investigator or delegate

**URGENT REFERRAL LETTER TO NURSE/ DOCTOR**

***(replaced at follow up with the combined letter above)***

To Whom It May Concern

Re: …………………………………………………….. (Patient’s name and age)

I interviewed the above patient today for a study that we are conducting on chronic disease care.

The interview contained questions about emotional wellbeing. During the interview, he/she became visibly distressed. I am concerned about his/her mental well being and would be grateful if you could assess his/her mental state and manage accordingly.

Many thanks for your assistance in this matter.

Yours sincerely

………………………………………………………… …………………………………….

Signature of Principal Investigator or delegate Date

………………………………………………………….……………………………………………………………

Printed name of Principal Investigator or delegate

Appendix 5: Referral letter for high triglyceride

**REFERRAL LETTER TO NURSE/ DOCTOR**

To Whom It May Concern

Re: …………………………………………………….. (Patient’s name and age)

This patient participated in a study that we are conducting to validate a non-laboratory based screening tool for cardiovascular disease risk prediction in South Africa and on chronic disease care.

He/she has a serum triglyceride level ≥15mmol/l which suggests that he/she is at risk of pancreatitis.

Would you kindly recall the patient and refer to hospital ***this week***. The patient must restrict fat intake to a maximum of 25 grams of fat per day. This means the patient should try to avoid as much fat in the diet as possible and use only skim milk and fat-free dairy products until he/she is seen at the hospital. We would be grateful if you could emphasise to the patient the importance of adhering to this advice in order to reduce the chance of developing pancreatitis.

Many thanks for your assistance in this matter.

Yours sincerely

………………………………………………………… …………………………………….

Signature of Principal Investigator or delegate Date

………………………………………………………….……………………………………………………………

Printed name of Principal Investigator or delegate

## Appendix 6: Patient information sheet/ consent form

**DEVELOPMENT AND EVALUATION OF TOOLS TO MANAGE CHRONIC NON-COMMUNICABLE DISEASE**

**INFORMATION SHEET AND CONSENT FORM FOR PARTICIPANTS**

Rec no IRB 00001938

Version Number: Revised 28 March 2011

We invite you to participate in a study. Before you agree to take part you need to understand what it involves.

**Purpose of study**

The purpose of the study is to evaluate a nurse training programme. Some clinics in the Eden district will receive the programme, which includes providing the nurses with, and training them in the use of a new guideline. Other clinics will continue with the usual care. We want to evaluate whether the new programme improves the treatment patients receive compared with usual care. We will also be looking at a new way to predict someone’s risk of developing a heart attack or stroke over the next 10 years.

**What are the possible benefits of participating in this study?**

The information that we obtain from the study will help us understand whether changing the way training is delivered results in improvements of care for people with chronic diseases and what costs are involved for patients (e.g. transport, GP visits etc.)

**What are the possible drawbacks or discomforts in participating in this study?**

We may ask you to have a blood sample taken. This will be the only discomfort in this study. Risk of infection will be minimized by using sterile procedures, and all blood samples will be taken by suitably qualified persons.

We estimate that the questionnaire will take approximately 20 to 40 minutes. We may want to interview you once more in about 14 months time. The second interview should be quicker than the first.

**Do I have to participate in this study?**

Your participation in this study is voluntary. Should you agree to participate, we will ask you to sign the attached form. You are free to withdraw from the study at any stage and this will in no way affect the care you receive at the clinic.

**What will happen to me if I participate?**

We will ask you some questions using a structured questionnaire and may record any medication you might be taking. We will then measure your height, weight, and the width around your waist and hip using a tape measure with your clothes on. We will also measure your blood pressure and may take a blood sample from your arm. We will take 15 ml of blood (3 teaspoons). The blood will be used to measure the level of fat in the blood and a test to see how high your blood sugar level is. The needle may cause you a little discomfort, but it will be taken in the way blood is usually taken from you when you attend the clinic. If any serious abnormal findings are identified we will inform the staff at the clinic who can then treat you appropriately.

We may want to see you again in about 14 months time. Then we will ask you some more questions like we will today and may also ask you for another blood sample to repeat the same tests. After the second time we see you we will provide you with a gift voucher to the value of R100 that you will be able to use in a shop near you, as a token of our appreciation in this important study.

We are also asking your permission to review your hospitalisation records, should you be hospitalised during the course of the study. We will also ask you for your South African identity number if available. This will allow linkage with a research copy of the Department of Home Affair’s databases to track your vital status. This research copy is securely stored by the Medical Research Council, and is used to complete research on the burden of diseases in South Africa. No identifiable information concerning your person will be made available to persons outside of the study, and even the researchers who will analyse the data will use datasets from which your identifiers will be removed.

**Will the information remain confidential?**

Should you agree to take part in the study all your records will be seen by the researchers only. Your information will not be seen by any other persons or parties not involved in this study.

**Contact details of the study staff:**

If you have any questions you can contact the following study staff members:

Name : Serena Van Haght Telephone number: 0795157481

If you have any complaints regarding this research study you may contact the University of Cape Town, Faculty of Health Science, Human Research Ethics Committee at (021 406-6492).

**Consent:**

I,……………………………………………………………………………………………………………………

(Name of participant in block letters)

have read and understood all the information given to me about my participation in this study and I have been given the opportunity to discuss it and ask questions. I voluntarily agree to take part in this study and understand that I will receive a copy of this consent form.

………………… ………..……………………… ……………………………………

Signature of Participant Date

I have explained the nature and purpose of the study to the participant named above.

………………………………………………………… …………………………………….

Signature of Principal Investigator or delegate Date

………………………………………………………….…………………………………………………………

Printed name of Principal Investigator or delegate

***Patient Information Leaflet***

**CHRONIC DISEASE STUDY**

**What is the study:**

We want to see whether a new guideline training programme for nurses improves the treatment patients receive. We will also be looking at a new way to predict someone’s risk of developing a heart attack or stroke over the next 10 years.

**Who can take part:**

We are looking for people who are 18 years or older and have certain criteria to take part in the study. If you are interested, a fieldworker will ask you some questions to see if you qualify to take part.

Your participation in this study is voluntary and the care you receive at the clinic will in no way be affected if you do not take part.

If you choose to take part, then you will be asked some questions about certain illnesses and any medication you might be taking. We will take some measurements (such as blood pressure, height and weight) and we may want to take a blood sample. We may also want to interview you again in about 14 months time.

**What to do if you are interested:**

If you are interested in taking part, please let the study fieldworker know and he/she will discuss it with you in more detail.
