# Supplementary material for: Educational Outreach with an Integrated Clinical Tool for Nurse-Led Non-communicable Chronic Disease Management in Primary Care in South Africa: A Pragmatic Cluster Randomised Controlled Trial
Source: PLoS Med. 2016 Nov 22;13(11):e1002178. doi: 10.1371/journal.pmed.1002178 (PMC5119726; doi:10.1371/journal.pmed.1002178)
Supplement: S4 Text — (PDF) [file pmed.1002178.s006.pdf]

Eligibility Screen

1	What is your home language	English	Afrikaans	IsiXhosa

2	Date of interview:
3	Clinic:
4	Interviewer Code:

We are conducting a study to evaluate care and risk factors for common chronic diseases, including diabetes, hypertension, chest conditions and depression, and we are looking for people with certain criteria to take part. I would like to start by asking you a few questions to see whether you qualify to take part in our study.

5	What is your name	First name	Surname
6	What is your date of birth	*if ≥18 goto 7*	*if <18 goto 25*
7	Enter sex	Male	Female
8	Are you planning to stay in the area for the next year	Yes*: goto 9*	No*: goto 18*
9	Are you taking medicine for high blood pressure (hypertension)	*Yes: goto 10*	*No: goto 10*
10	Are you taking medicine for diabetes (‘sugar’)	*Yes: goto 11*	*No: goto 11*
11	Are you taking medicine for asthma or chronic bronchitis or emphysema	*Yes: goto 13*	*No: goto 12A*
12A	Do you have cough or difficult breathing which has lasted for more than 2 weeks	*Yes: goto 12B*	*No goto 13*
12B	Have you been on treatment for TB in the past 3 months *[Yes to 12A and No to 12B = yes for question 12]*	*Yes: goto 13*	*No: goto 13*

13 We would like to know how your general well-being has been over the past week.
I am going to read a list of some of the ways you may have felt or behaved during the last week. Using the showcard, please indicate how often you have felt this way during the **past week**.

Interviewer mark one option on each line

**During the past week...**	Rarely or none of the time (less than 1 day)	Some or little of the time (1-2 days)	Occasionally or a moderate amount of time (3-4 days)	All of the time (5-7 days)
I was bothered by things that usually don’t bother me	0	1	2	3
I had trouble keeping my mind on what I was doing	0	1	2	3
I felt depressed	0	1	2	3
I felt that everything I did was an effort	0	1	2	3
I felt hopeful about the future	0	1	2	3
I felt fearful	0	1	2	3
My sleep was restless	0	1	2	3
I was happy	0	1	2	3
I felt lonely	0	1	2	3
I could not “get going”	0	1	2	3

TOTAL SCORE:

Eligible for trial if one or more of the following:<br><br>• ***YES to question 9 (hypertension cohort)***<br>• ***YES to question 10 (diabetes cohort)***<br>• ***YES to question 11 (respiratory cohort)***<br>• ***YES to question 12A AND NO to question 12B (respiratory cohort)***<br>• ***Score of 10 or more for question 13 (depression cohort)***	***Yes: eligible for trial: goto 14***	***No: not eligible for trial: goto 18***
*Interviewer to fill in **enrolment log** (paper chart) to keep a record of how many patients in each of the 4 cohorts (hypertension, diabetes, chronic respiratory disease and depression risk score ≥10) the clinic has recruited.*		

14	***[eligible for trial, now see if eligible for validation study as well with questions 14-17]***<br>*Computer to draw age from previous question*	*If ≥35 goto 15.*	*If <35 goto 22*
15	Has a doctor or nurse ever told you that you have or have had any of the following:<br>*15A* a heart attack *yes* *no*<br>*15B* a stroke *yes* *no*<br>*15C* angina (chest pains with exertion/activity) *yes* *no*	*If YES to any of the three (15A, 15B or 15C) then eligible for trial only: goto 22*	*If NO to all three (15A, 15B and 15C) then goto 16*
16	Do you have a South African identity (ID) number	*Yes: goto 17*	*No: eligible for trial only: goto 22*
17	Would you be able to give me your ID number today	*Yes: eligible for trial and validation: do **not** enter ID number at this stage: goto 23*	*No: eligible for trial only: goto 22*

18	***[not eligible for trial, now assess whether eligible for validation study with questions 18-21]***<br>*Computer to draw age from previous question*	*If ≥35 goto 19*	*If <35 goto 25*
19	Has a doctor or nurse ever told you that you have or have had any of the following:<br><br>19A a heart attack yes no<br>19B a stroke yes no<br>19C angina (chest pains with exertion/activity) yes no	*If YES to any of the three (19A, 19B or 19C) then not eligible for trial or study: goto 25*	*If NO to all three (19A, 19B and 19C) goto 20*
20	Do you have a South African identity (ID) number	*Yes: goto 21*	*No: not eligible for trial or validation study: goto 25*
21	Would you be able to give me your ID number today	*Yes: eligible for validation study only: do **not** enter ID number at this stage: goto 24*	*No: not eligible for trial or validation study: goto 25*

22	***[Eligible for trial only]**:*<br><br>You qualify for our study and we would like you to take part.<br><br>I will go through the written information with you and if you would like to take part then we will ask you to sign the form saying you are willing to participate.<br><br>I will then go through the questionnaire with you, check your measurements (blood pressure, weight, height, waist circumference and hip measurement), and we may want to take a blood sample*. [HbA1c]*<br><br>*[Interviewer to go through the patient information and consent form. A study number will then be allocated]*
23	***[Eligible for trial and validation]:***<br><br>You qualify for our study and we would like you to take part.<br><br>I will go through the written information with you and if you would like to take part then we will ask you to sign the form saying you are willing to participate.<br><br>I will then go through the questionnaire with you, check your measurements (blood pressure, weight, height, waist circumference and hip measurement), and we may want to take a blood sample [HbA1c, lipids]<br><br>*[Interviewer to go through the patient information and consent form. A study number will then be allocated]*
24	*[**Eligible for validation only]**:*<br><br>You qualify for our shorter questionnaire and we would like you to take part.<br><br>I will go through the written information with you and if you would like to take part then we will ask you to sign the form saying you are willing to participate.<br><br>I will then go through the questionnaire with you, check your measurements (blood pressure, weight, height, waist circumference and hip measurement), and we may want to take a blood sample *[HbA1c, lipids]*<br><br>*[Interviewer to go through the patient information and consent form. A study number will then be allocated]*
25	***[Not eligible for trial or validation study]***<br><br>We are looking for people with certain criteria or illnesses to take part in the study. You do not have the criteria we are looking for so we will not be able to include you in the study. Your usual care will not be affected by not taking part in our study. We would like to thank you very much for answering the questions and for your time today and we wish you well.<br><br>*[Interviewer to document that the eligibility screen was done and that the patient is **not eligible for trial or validation** study. No further action required.]*

Questionnaire 1:

[Conducted immediately after the eligibility screen for patients eligible to take part in the trial and validation study, or just the trial.]

I am now going to go through the questionnaire with you. We will need to take three blood pressure readings during the course of the interview, at least two minutes apart. *[interviewer to take first blood pressure reading now].*

I would like to start by asking you a few questions about your past illnesses:

1	Has a doctor or nurse ever told you that you have or have had any of the following		
1A	high blood pressure (hypertension)	*Yes*	*No*
1B	heart attack	*Yes*	*No*
1C	stroke	*Yes*	*No*
1D	angina (chest pains with exertion/activity)	*Yes*	*No*
1E	depression	*Yes*	*No*
1F	TB	*Yes*	*No*
1G	diabetes (‘sugar’)	*Yes*	*No*

2	I am now going to ask you some questions about smoking		
2A	Do you currently smoke cigarettes daily	*Yes: goto 2B*	*No: goto 2E*
2B	How old were you when you first started smoking daily	Age in years	Don’t remember/not sure
2C	On average, how many cigarettes do you smoke each day	*Enter number*	
2D	Have you had advice from a health worker to stop smoking in the past year	*Yes: goto 2J*	*No: goto 2J*

2E	In the past, did you ever smoke daily	*Yes: goto 2F*	*No: goto 2J*
2F	How old were you when you first started smoking daily	Age in years	Don’t remember/not sure
2G	How old were you when you stopped smoking daily	Age in years	Don’t remember/not sure
2H	On average, how many cigarettes did you smoke each day	*Enter number and goto 2J*	

2J	Are you currently a smoker. Choose one of the following options	Yes, I currently smoke *Goto 2K*	No, I quit within the last 6 months *Goto 3*	No, I quit more than 6 months ago *Goto 3*	No, I have never smoked *Goto 3*
2K	In the last year, how many times have you quit smoking for at least 24 hours	*Enter number: Goto 2L*			
2L	Are you seriously thinking of quitting smoking. Choose one of the following options	Yes, within the next 30 days	Yes, within the next 6 months	No, not thinking of quitting	

3	I would like to ask about your current state of health. Please indicate which of the following statements best describe your health state TODAY (choose one option per group)

Mobility:
I have no problems in walking about ❑
I have some problems in walking about ❑
I am confined to bed ❑

Self-Care
I have no problems with self-care ❑
I have some problems washing or dressing myself ❑
I am unable to wash or dress myself ❑

Usual Activities (e.g. work, study, housework, family or leisure activities)
I have no problems with performing my usual activities ❑
I have some problems with performing my usual activities ❑
I am unable to perform my usual activities ❑

Pain/Discomfort
I have no pain or discomfort ❑
I have moderate pain or discomfort ❑
I have extreme pain or discomfort ❑

Anxiety/Depression
I am not anxious or depressed ❑
I am moderately anxious or depressed ❑
I am extremely anxious or depressed ❑

To help people say how good or bad their state of health is, we have drawn a scale (rather like a thermometer) on which the best state you can imagine is marked 100 and the worst state you can imagine is marked 0.

We would like you to indicate on this scale, in your opinion, how good or bad your own health is today. Please do this by drawing a line from the box below to whichever point on the scale indicates how good or bad your state of health is today.

Your own state of health today

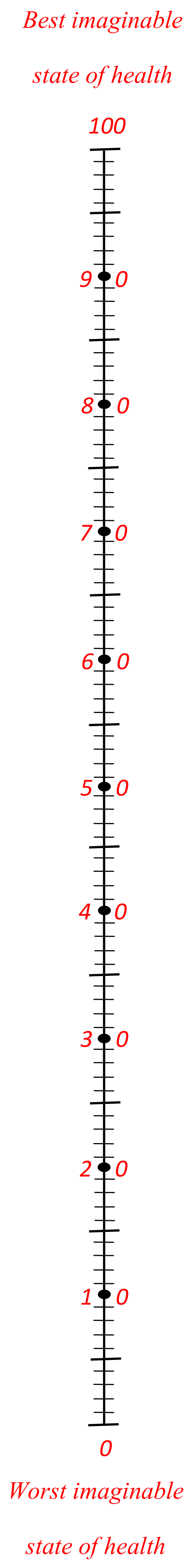

4	*[Modified St Georges Questionnaire for chronic respiratory disease patients only]*<br>I would now like to ask you some more detailed questions about your cough or breathing problem

**Questions about how much chest trouble you have had over the past 3 months.** Please tick one box for each question:					
Over the past 3 months, I have coughed:	Most days a week	Several days a week	A few days a month	Only with chest infections	Not at all
Over the past 3 months, I have brought up phlegm (sputum):	Most days a week	Several days a week	A few days a month	Only with chest infections	Not at all
Over the past 3 months, I have had shortness of breath:	Most days a week	Several days a week	A few days a month	Only with chest infections	Not at all
Over the past 3 months, I have had attacks of wheezing:	Most days a week	Several days a week	A few days a month	Only with chest infections	Not at all
During the past 3 months how many severe or very unpleasant attacks of chest trouble have you had?<br>Please tick one:	More than 3 attacks	3 attacks	2 attacks	1 attack	No attacks

How long did the worst attack of chest trouble last?<br>(Go to next question if you had no severe attacks) please tick one:	A week or more	3 or more days	1 or 2 days	Less than a day

Over the past 3 months, in an average week, how many good days (with little chest trouble) have you had?<br>Please tick one:	No good days	1 or 2 good days	3 or 4 good days	Nearly every day is good	Every day is good

If you have a wheeze, is it worse in the morning? Please tick one:	yes	no

**Questions about what activities usually make you feel breathless <u>these days</u>.**<br>Please tick in each box that applies to you these days:		
	True	False
Sitting or lying still		
Getting washed or dressed		
Walking around the home		
Walking outside on the level		
Walking up a flight of stairs		
Walking up hills		
Playing sports or games		

**These are questions about how your activities might be affected by your breathing.**<br>Please tick in each box that applies to you **because of your breathing**:		
	True	False
I take a long time to get washed or dressed		
I cannot take a bath or shower, or I take a long time		
I walk slower than other people, or I stop for rests		
Jobs such as housework take a long time, or I have to stop for rests		
If I walk up one flight of stairs, I have to go slowly or stop		
If I hurry or walk fast, I have to stop or slow down		
My breathing makes it difficult to do things such as walk up hills, carrying things up stairs, light gardening such as weeding, dance, play bowls or play golf		
My breathing makes it difficult to do things such as carry heavy loads, dig the garden, jog or walk at 8 kilometers per hour, play tennis or swim		
My breathing makes it difficult to do things such as very heavy manual work, run, cycle, swim fast or play competitive sports		

5	I am now going to ask you some questions about the health care you have received and the medicines you were given in the last year		
5A	Has a health worker examined the back of your eyes in the last year *[show picture of ophthalmoscope eye exam]*	*yes*	*no*
5B	Has a health worker examined your feet in the last year with any of the following *[showcard picture of foot, tuning fork, pin, cotton wool]*	*yes*	*no*
5C	Have you received counselling from any of the following people in the last year (counseling is not just receiving advice on how to take medication. It means talking with someone in a way that helps you to find solutions to your problems, or receive emotional support)		

Doctor at a clinic/outpatients	*Yes*	*No*
Doctor at a general practice	*Yes*	*No*
Nurse	*Yes*	*No*
Mental health nurse	*Yes*	*No*
Clinic counsellor	*Yes*	*No*
Social worker	*Yes*	*No*
Psychiatrist or psychologist	*Yes*	*No*
Religious counsellor, traditional healer or faith healer	*Yes*	*No*

5G	Have you had a ‘flu vaccine in the last year	*Yes*	*No*

5H	Has a health worker given you advice in the last year about weight management			*Yes*	*No*		
5I	How often do you exercise vigorously enough to work up a sweat. Choose one of the following options:	Rarely/ never	1 to 3 times per month	1 time per week	2 to 4 times per week	5 to 6 times per week	daily
5J	Are you taking any medicine regularly that was prescribed by a doctor or nurse			*Yes: goto 5K*	*No: goto 5L*		
5K	I am now going to look at your file to see what medicines have been prescribed over the last year						
5L	Are you currently taking any medicine for TB			*yes*	*no*		
5M	Are you currently taking any ARVs (antiretrovirals)			*yes*	*no*		
5N	How often do you miss your medication, either forgetting to take it or deciding not to			Never/very rarely	sometimes	often	
5O	I am now going to take your second blood pressure reading						

6 We would like to understand how much time and money your illness costs you. In order to do this we need to ask some questions about your use of health care services, your employment status and income. We would like to remind you that all the information you give us is confidential.						
6A Have you had a chest x-ray over the last 3 months	*Yes goto 6B*					*No goto 6C*
6B Please state how many chest x-rays you have had over the last 3 months	*Enter number*					
6C Have you had blood tests over the last 3 months	*Yes*			*No*		
6D How do you usually travel to this clinic (choose one of the following options)	Walk *goto 6H*	Taxi: *goto 6F*	Private motor vehicle (such as car) *Goto 6E*	Bus *goto 6F*	Patient transport/ ambulance *goto 6F*	Other (specify) *goto 6F*
6E What was the distance travelled to the clinic (in km one way) then goto 6H	*Enter distance in km one way*					
6F Do you usually pay a transport fare	*Yes:Goto 6G*			*No: goto 6H*		
6G How much do you pay for a return fare	*Enter amount in rands*					
6H Please detail other costs usually associated with your visit	Accommodation *(enter amount in rands)*			Food *(enter amount in rands)*		
6I Aside from today, have you visited this clinic in the last 3 months	*Yes: goto 6J*			*No goto 6K*		
6J How many times have you visited this clinic in the last 3 months (excluding today)	*Enter number of visits*					
6K Have you visited any other health care provider in the last 3 months	*Yes: goto 6L*			*No: goto 6M*		

6I	Please indicate which of the following health care providers you have visited in the last 3 months. I will also ask you some more details about those visits.

i) Another clinic
ii) Hospital (outpatient visits only)
iii) General Practitioner
iv) Private Pharmacy
v) Traditional healer/ herbalist
vi) Other (please state)

For each option selected, the following details will be asked:

a)	Number of visits in the last 3 months						
b)	Did you pay a fee on your last visit				Yes		no
c)	*If yes:* What was the fee in rands				R		
d)	What transport did you use to get to your last visit	i)walk	Ii)taxi	iii)car	iv)bus	v) patient transport/ ambulance	vi)other
e)	Did you pay a transport fare on your last visit				Yes		no
f)	*If yes:* What was the return fare in rands				R		
g)	If you used your own car, what was the distance travelled (in kilometers one way)						
h)	Please detail other costs associated with your last visit				i)accommodation		ii)food

6M Have you been admitted to hospital in the last 3 months	*Yes: goto 6N*	*No: goto 6U*
6N How many times have you been admitted to hospital in the last 3 months	*Enter number:*	
6O How many nights in total have you spent in hospital over the last 3 months:	*Enter number:*	
6P What is the total amount you have had to pay for your admissions over the last 3 months (your out-of-pocket expenses)	*Enter amount in rands:*	

6Q For the most recent admission to hospital please provide the following details:

How did you travel to the hospital (choose one of the following options):	Walk: *goto 6U*	Taxi: *goto 6S*	Private motor vehicle (such as car) *Goto 6R*	Bus: *goto 6S*	Patient transport/ ambulance *goto 6S*	Other (specify): *goto 6S*

6R What was the distance travelled to the hospital (in km one way) *then goto 6U*	*Enter distance in km one way:*	
6S Did you pay a transport fare	*Yes:Goto 6T*	*No: goto 6U*
6T How much did you pay for a return fare	*Enter amount in rands*	

6U Which of the following best describes your employment status? Choose *one* :	
Employed	How much did you earn last month (excluding grant income)
	How many days were you unable to work because of illness in the last 3 months (including health care visits)

	How much income have you lost in the last 3 months as a result of not being able to work because of any illness
Self-employed	How much did you earn last month (excluding grant income)
	How many days were you unable to work because of illness in the last 3 months (including health care visits)
	How much income have you lost in the last 3 months as a result of not being able to work because of any illness
Student/learner	How many days have you been unable to attend school/college because of illness in the last 3 months (including health care visits)
Unemployed and looking for work	
Unemployed and not looking for work	

6V Are you getting a pension or grant (eg disability or child care grant)	*Yes: goto 6W*	*No: goto 6X*
6W What was your total grant income in the last month	*Enter amount in rands*	
6X Have you lost your job or resigned because of illness during the past year	*Yes: goto 6Y*	*No: goto 7*
6Y Before you lost your job or resigned, how much did you earn in the last month you worked (excluding grant income)	*Enter amount in rands*	
6Z Since losing your job or resigning, have you got another job	*Yes: goto 7*	*No: goto 7*
8 I am now going to take your third blood pressure reading, height, weight, waist circumference and hip measurement.		
9 If a blood test is required: When did you last have anything to eat or drink, other than water	Date:	Time:
10 It is important for our study that we interview you once more, in 14 months time. I will schedule that appointment for you now and we will send you SMS reminders from 3 months before the appointment. I will provide you with a contact number. Please let us know if your cell phone number changes or if you need to reschedule the appointment. In order to make it as easy as possible to contact you if necessary, I would be grateful if you could provide as many of the following contact details as possible:		

Home address	
Telephone number at home	
Cell phone number	
Work address	
Work telephone number	
Alternative number (friend, relative, neighbour)	
Clinic folder number	
Re-enter clinic folder number	
Name of hospital patient attends	
Hospital folder number if available	
ID number	
Re-enter ID number	

We would like to thank you very much for your time today. We look forward to seeing you next year.

Questionnaire 2: for patients eligible for Validation Study only:

I am now going to go through the questionnaire with you. We will need to take three blood pressure readings during the course of the interview, at least two minutes apart. *[interviewer to take first blood pressure reading now].*

1. Has a doctor or nurse ever told you that you have or have had diabetes ('sugar')	*Yes*	*No*

2.: Are you taking any medicine regularly that was prescribed by a doctor or nurse for high blood pressure (hypertension) *fieldworker to confirm from prescription charts*	*Yes*	*No*

3. Are you taking any medicine regularly that was prescribed by a doctor or nurse for cholesterol *fieldworker to confirm from prescription charts*	*Yes*	*No*

4. I am now going to look at your file to see what medicines have been prescribed for hypertension and cholesterol

[interviewer to take second blood pressure reading if ≥2 minutes since first reading]

5. Do you currently smoke cigarettes daily	Yes:	No:

6. How often do you exercise vigorously enough to work up a sweat. Choose one of the following options:	Rarely/ never	1 to 3 times per month	1 time per week	2 to 4 times per week	5 to 6 times per week	daily

7. In case we need to contact you, I would be grateful if you could provide as many of the following contact details as possible:	
Home address	
Telephone number at home	
Cell phone number	
Work address	
Work telephone number	
Alternative number (friend, relative, neighbour)	
Clinic folder number	
Re-enter clinic folder number	
Name of hospital patient attends	
Hospital folder number if available	
ID number	
Re-enter ID number	

8. I am now going to take your third blood pressure reading, height, weight, waist circumference and hip measurement. You will require a blood test. You will not require a blood test		
9 .If a blood test is required: When did you last have anything to eat or drink, other than water	Date:	Time:

We would like to thank you very much for your time today. We will not need to interview you again and we wish you well.
