# Supplementary material for: Educational Outreach with an Integrated Clinical Tool for Nurse-Led Non-communicable Chronic Disease Management in Primary Care in South Africa: A Pragmatic Cluster Randomised Controlled Trial
Source: PLoS Med. 2016 Nov 22;13(11):e1002178. doi: 10.1371/journal.pmed.1002178 (PMC5119726; doi:10.1371/journal.pmed.1002178)
Supplement: S5 Text — (PDF) [file pmed.1002178.s007.pdf]

Patient information sheet

Rec no IRB 00001938
Version Number: Revised 28 March 2011

We invite you to participate in a study. Before you agree to take part you need to understand what it involves.

Purpose of study

The purpose of the study is to evaluate a nurse training programme. Some clinics in the Eden district will receive the programme, which includes providing the nurses with, and training them in the use of a new guideline. Other clinics will continue with the usual care. We want to evaluate whether the new programme improves the treatment patients receive compared with usual care. We will also be looking at a new way to predict someone's risk of developing a heart attack or stroke over the next 10 years.

What are the possible benefits of participating in this study?

The information that we obtain from the study will help us understand whether changing the way training is delivered results in improvements of care for people with chronic diseases and what costs are involved for patients (e.g. transport, GP visits etc.)

What are the possible drawbacks or discomforts in participating in this study?

We may ask you to have a blood sample taken. This will be the only discomfort in this study. Risk of infection will be minimized by using sterile procedures, and all blood samples will be taken by suitably qualified persons.

We estimate that the questionnaire will take approximately 20 to 40 minutes. We may want to interview you once more in about 14 months time. The second interview should be quicker than the first.

Do I have to participate in this study?

Your participation in this study is voluntary. Should you agree to participate, we will ask you to sign the attached form. You are free to withdraw from the study at any stage and this will in no way affect the care you receive at the clinic.

What will happen to me if I participate?

We will ask you some questions using a structured questionnaire and may record any medication you might be taking. We will then measure your height, weight, and the width around your waist and hip using a tape measure with your clothes on. We will also measure your blood pressure and may take a blood sample from your arm. We will take 15 ml of blood (3 teaspoons). The blood will be used to measure the level of fat in the blood and a test to see how high your blood sugar level is. The needle may cause you a little discomfort, but it will be taken in the way blood is usually taken from you when you attend the clinic. If any serious abnormal findings are identified we will inform the staff at the clinic who can then treat you appropriately.

We may want to see you again in about 14 months time. Then we will ask you some more questions like we will today and may also ask you for another blood sample to repeat the same tests. After the second time we see you we will provide you with a gift voucher to the value of R100 that you will be able to use in a shop near you, as a token of our appreciation in this important study.

We are also asking your permission to review your hospitalisation records, should you be hospitalised during the course of the study. We will also ask you for your South African identity number if available. This will allow linkage with a research copy of the Department of Home Affair's databases to track your vital status. This research copy is securely stored by the Medical Research Council, and is used to complete research on the burden of diseases in South Africa. No identifiable information concerning your person will be made available to persons outside of the

study, and even the researchers who will analyse the data will use datasets from which your identifiers will be removed.

Will the information remain confidential?

Should you agree to take part in the study all your records will be seen by the researchers only. Your information will not be seen by any other persons or parties not involved in this study.

Contact details of the study staff:

If you have any questions you can contact the following study staff members:

Name : Serena Van Haght Telephone number:

If you have any complaints regarding this research study you may contact the University of Cape Town, Faculty of Health Science, Human Research Ethics Committee at (021 406-6492).

Consent:

I,………

(Name of participant in block letters)

have read and understood all the information given to me about my participation in this study and I have been given the opportunity to discuss it and ask questions. I voluntarily agree to take part in this study and understand that I will receive a copy of this consent form.

………………… ………..……………………… ……………………………………

Signature of Participant Date

I have explained the nature and purpose of the study to the participant named above.

………………………………………………………… …………………………………….

Signature of Principal Investigator or delegate Date

…………………………………………………………..…………………………………………………

Printed name of Principal Investigator or delegate
